# Three enigmatic BioH isoenzymes are programmed in the early stage of mycobacterial biotin synthesis, an attractive anti-TB drug target

**DOI:** 10.1371/journal.ppat.1010615

**Published:** 2022-07-11

**Authors:** Yongchang Xu, Jie Yang, Weihui Li, Shuaijie Song, Yu Shi, Lihan Wu, Jingdu Sun, Mengyun Hou, Jinzi Wang, Xu Jia, Huimin Zhang, Man Huang, Ting Lu, Jianhua Gan, Youjun Feng

**Affiliations:** 1 Departments of Microbiology, and General Intensive Care Unit of the Second Affiliated Hospital, Zhejiang University School of Medicine, Hangzhou, Zhejiang, The People’s Republic of China; 2 Shanghai Public Health Clinical Center, State Key Laboratory of Genetic Engineering, Collaborative Innovation Center of Genetics and Development, Department of Biochemistry and Biophysics, School of Life Science, Fudan University, Shanghai, The People’s Republic of China; 3 State Key Laboratory for Conservation and Utilization of Subtropical Agro-bioresources, College of Life Science and Technology, Guangxi University, Nanning, Guangxi, The People’s Republic of China; 4 College of Animal Sciences, Zhejiang University, Hangzhou, Zhejiang, The People’s Republic of China; 5 Guangxi Key Laboratory of Utilization of Microbial and Botanical Resources & Guangxi Key Laboratory for Polysaccharide Materials and Modifications, School of Marine Sciences and Biotechnology, Guangxi Minzu University, Nanning, Guangxi, The People’s Republic of China; 6 Non-coding RNA and Drug Discovery Key Laboratory of Sichuan Province, Chengdu Medical College, Chengdu, Sichuan, The People’s Republic of China; 7 Cancer Center at Illinois, University of Illinois at Urbana-Champaign, Urbana, Illinois, United States of America; 8 Department of Bioengineering, University of Illinois at Urbana-Champaign, Urbana, Illinois, United States of America; National Institutes of Health, UNITED STATES

## Abstract

Tuberculosis (TB) is one of the leading infectious diseases of global concern, and one quarter of the world’s population are TB carriers. Biotin metabolism appears to be an attractive anti-TB drug target. However, the first-stage of mycobacterial biotin synthesis is fragmentarily understood. Here we report that three evolutionarily-distinct BioH isoenzymes (BioH1 to BioH3) are programmed in biotin synthesis of *Mycobacterium smegmatis*. Expression of an individual *bioH* isoform is sufficient to allow the growth of an *Escherichia coli* Δ*bioH* mutant on the non-permissive condition lacking biotin. The enzymatic activity *in vitro* combined with biotin bioassay *in vivo* reveals that BioH2 and BioH3 are capable of removing methyl moiety from pimeloyl-ACP methyl ester to give pimeloyl-ACP, a cognate precursor for biotin synthesis. In particular, we determine the crystal structure of dimeric BioH3 at 2.27Å, featuring a unique lid domain. Apart from its catalytic triad, we also dissect the substrate recognition of BioH3 by pimeloyl-ACP methyl ester. The removal of triple *bioH* isoforms (Δ*bioH1*/*2*/*3*) renders *M*. *smegmatis* biotin auxotrophic. Along with the newly-identified Tam/BioC, the discovery of three unusual BioH isoforms defines an atypical ‘BioC-BioH(3)’ paradigm for the first-stage of mycobacterial biotin synthesis. This study solves a long-standing puzzle in mycobacterial nutritional immunity, providing an alternative anti-TB drug target.

## Author summary

Biotin (vitamin B7) is essential for nutritional immunity in TB-causing bacterium, *M*. *tuberculosis*. Here, we show that three distinct BioH isoforms (BioH1 to BioH3) are programmed in the first-stage of mycobacterial biotin synthesis, a validated anti-TB drug target. BioH2 and BioH3 are active in the hydrolysis of M-C7-ACP to give C7-ACP, a *bona fide* precursor of the late step of biotin synthesis. The 2.27Å x-ray structure of BioH3 dimer allows us to illustrate its catalysis mechanism. The triple mutant of Δ*bioH1*/*2*/*3* is biotin auxotrophic. The two biochemically-amenable enzymes BioH2 and BioH3 enables the *in vitro* reconstitution of biotin synthesis. Combined with the recently-identified Tam/BioC, characterization of three mysterious BioH isoenzymes defines an atypical paradigm of ‘BioC-BioH(3)’ for the early step of mycobacterial biotin synthesis.

## Introduction

Tuberculosis (TB) is a chronic disease caused by the bacillus *Mycobacterium tuberculosis* (*M*. *tuberculosis*) of global concern [1,2]. Globally in 2020, TB incidence is estimated by the World Health Organization (WHO) to be about 9.9 million, of which TB deaths is above 1.5 million [3]. As a representative of facultative/intracellular pathogens [4], *M*. *tuberculosis* displays certain metabolic versatility to survive in harsh host environment [5,6]. An obstacle to control this TB-causing pathogen, partially stems from its transition between active stage and latent stage [7–9]. As a result, this evades antibiotic killing, and bypasses host immunity [5,6,8]. Currently, it is estimated by the WHO that one quarter of the world’s population are involved in latent infections with *M*. *tuberculosis* [2,3,10]. More worrisomely, multidrug-resistant TB (MDR-TB) disseminates worldwide, and constitutes a public health crisis [1,3]. This is because it compromises isoniazid and rifampicin, the two first-line anti-TB drugs [1,3,11]. To prevent global spread of MDR-TB [3,12], it is necessary to call for the development of next-generation anti-TB drugs with novel targets [1,3]. Recently, biotin biosynthetic pathway has been validated as an attractive drug target against active TB [13,14], which is evidenced by the discovery of a number of lead compounds/inhibitors against enzymes involved in mycobacterial biotin synthesis [10,13,15] and its subsequent utilization [11,16]. However, the early stage of mycobacterial biotin synthesis is poorly understood [17].

The covalently-linked coenzyme, biotin (also called vitamin B7), is a ubiquitous micronutrient throughout the three domains of life [18,19]. This is because it plays inevitable roles in CO_2_ fixation of certain intermediate metabolic pathways, namely lipid synthesis, amino acids catabolism and glucogenesis [20,21]. The vitamin cofactor, biotin consists of two fused heterocyclic rings decorated with a valeric acid side chain [22,23]. In general, *de novo* synthesis pathway of biotin is divided into two stages (early stage and late stage) [18,21,24]. Namely, the first-stage is dedicated to the formation of biotin precursor, pimelate (a 7-carbon α, ω-dicarboxylic acid) [23], and the late stage is engaged in the assembly of two fused rings of biotin [21]. It is long settled that the assembly of two biotin rings is a conserved ‘four-step’ pathway successively catalyzed by BioF (8-amino-7-oxononanoate synthase, AON synthase) [25], BioA (7,8-diaminononanoate synthase, DAN synthase) [26], BioD (dethiobiotin synthase, DTB synthase) [27,28] and BioB (biotin synthase) [27,29]. It was noted that the newly-identified dehydrogenase BioU from cyanobacteria replaces BioA [22], and behaves as a suicide enzyme losing the lysine 124 (K124) residue after a complete round of reactions for biotin ring formation [30]. Unlike the largely-conserved late step, the route of pimeloyl moiety formation differs markedly in diversified biotin-producing microorganisms [22]. So far, three types of distinct mechanisms have been discovered, which provides 7 of the 10 biotin carbon atoms [22]. Namely, these include i) ‘BioC-BioH’ pathway [23,31,32], ii) ‘BioI-BioW’ route [33–35], and iii) BioZ machinery [36,37]. First, the paradigm ‘BioC-BioH’ model produces pimeloyl-ACP by hijacking a modified type II fatty acid synthetic (FAS II) pathway [23,32]. The BioC-aided methylation enables the resultant malonyl-ACP (CoA) methyl ester to undergo two reiterations of FAS II cycles [32], generating methyl pimeloyl-ACP thioester that is subsequently hydrolyzed by the promiscuous esterase BioH to give pimeloyl-ACP (pim-ACP or C7-ACP), a precursor for biotin synthesis [23,31]. To the best of our knowledge, six isoenzymes of BioH have been detected in various bacterial species, namely BioG [38,39], BioK [38], BioJ [40,41], BioV [42], BtsA [43], and BioUh [22,44]. As for ‘BioI-BioW’ model of *Bacillus subtilis*, the cytochrome P450 protein BioI presumably cleaves long-chain fatty acids (and/or acyl-ACP) to give pimeloyl-ACP [35,45], whereas the *bona fide* acyl-CoA synthetase BioW scavenges exogenous pimelate to yield pimeloyl-CoA thioester [33,34,46], a substrate specifically recognized by the *Bacillus* BioF [25,45]. In the phylum of α-proteobacteria (consisting of the plant pathogen *Agrobacterium* [47,48], the zoonotic agent *Brucella* [49], and the symbiotic *Mesorhizobium* [50,51]), an atypical β-ketoacyl-ACP synthase BioZ is dedicated to the condensation of glutaryl-CoA (ACP) with malonyl-ACP to produce C7-ACP thioester [36,37].

*M*. *smegmatis* is a fast-growing surrogate for studying the human pathogen *M*. *tuberculosis* of slow growth. Presumably, the BioC is annotated as a methyltransferase, and the BioH demethylase belongs to the family of α/β-hydrolase. Genomic analysis suggests that Mycobacteria probably employ the ‘BioC-BioH’ strategy to begin biotin synthetic pathway [9]. However, a long-standing puzzle in this field is the lack of functional assignment of ‘BioC-BioH’ pair. *M*. *smegmatis* is unusual because that its chromosome encodes 72 BioC-like genes and 90 BioH homologs (**S1 and S2 Tables**) [52]. The unexpected complexity does appear as a conundrum to close in on mycobacterial biotin synthesis. Very recently, Hu and Cronan reported that *M*. *smegmatis* BioC is Tam, which essentially catalyzes the methylation of trans-aconitate to give cis-aconitate [17]. Intriguingly, expression cloning allowed us to delineate three distinct BioH isoforms from *M*. *smegmatis*, namely MSMEG_2036 (BioH1), MSMEG_1352 (BioH2), and MSMEG_6710 (BioH3). The removal of all the three *bioH* loci (*bioH1* to *bioH3*) renders *M*. *smegmatis* biotin auxotrophic. Continued efforts enable us to report a high-resolution structure of dimeric BioH3, of which lid domain is far different from those of all the monomeric BioH/J/G gatekeeper enzymes with known structures. This poses the possibility that BioH cousins are less domesticated during the mycobacterial evolution [4]. Together with the proposal of BioC by Hu and Cronan [17], these findings offer an unusual ‘BioC-BioH(3)’ paradigm for the first-stage of mycobacterial biotin synthesis, in which three mysterious BioH cousins are programmed.

## Results and discussion

### The ‘BioC-BioH(3)’ pathway in *M*. *smegmatis*

**Fig 1:**
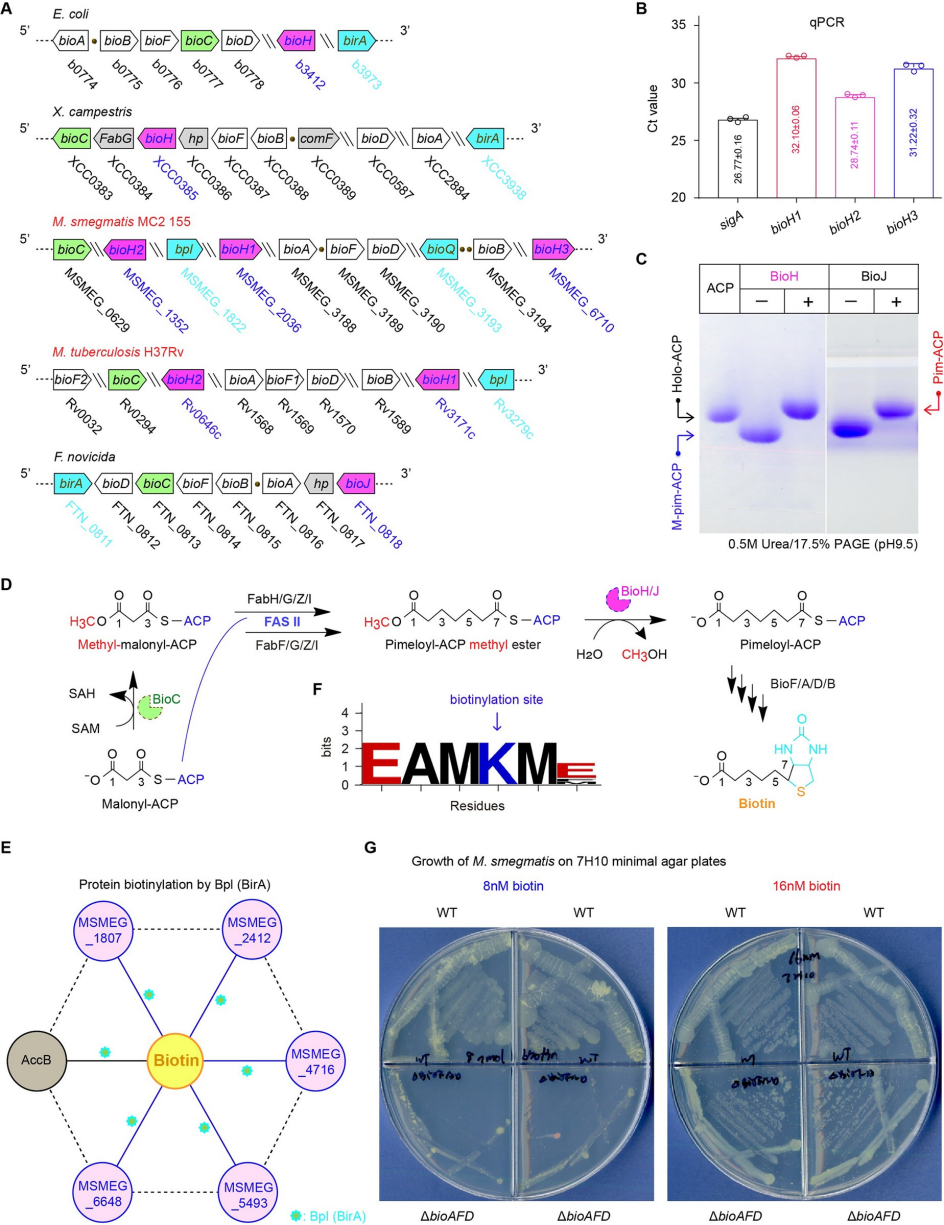
Scheme for BioH action in mycobacterial biotin biosynthetic pathway. **A.** Genetic environment of *bioH* homologs in different microorganisms. The dots denote the conserved sequence motifs recognized by the regulatory protein (e.g., BirA and/or BioQ, shown in cyan background). The two discontinued loci are separated with the double slash. The *bioC* is colored green, and the *bioH* homolog is highlighted in purple. **B.** Real-time quantitative PCR (qPCR) analysis for expression of three putative BioH paralogs (*bioH1* to *bioH3*) Namely, the three *bioH* isoforms included *bioH1* (MSMEG_2036), *bioH2* (MSMEG_1352), and *bioH3* (MSMEG_6710). *sigA* encoding sigma A functions as an interference gene. Ct (cycle threshold) is used to measure the amplification cycles of target genes during the qPCR. **C.** Use of conformationally-sensitive 0.5M urea/PAGE (17.5%, pH9.5) to separate reactant M-pim-ACP and its hydrolytic product pim-ACP The minus “—” denotes no addition of either BioH or BioJ enzyme. **D.** The schematic representative of the “BioC-BioH” pathway of biotin synthesis. **E.** A scheme for multi-target biotinylation by Bpl in *M*. *smegmatis*. Unlike that in *E*. *coli* AccB (colored gray) is the only biotinylated enzyme, *M*. *smegmatis* is proposed to contain no less than five target proteins (colored pink) modified by Bpl. **F.** Sequence signature of the protein biotinylation by Bpl. **G.** Bacterial viability-based determination for minimum physiological demand for biotin in *M*. *smegmatis* MC^2^ 155. Designations: BioA, 7,8-diaminononanoate synthase (DANS); BioB, biotin synthase; BioF, 8-amino-7-oxononanoate synthase (AONS); BioC, O-methyl transferase; BioD, dethiobiotin synthetase (DTBS); BioH, methyl pimeloyl-ACP ester carboxyl-esterase; BirA, biotin protein ligase/repressor; Bpl, biotin protein lgase; HP, hypothetical protein; ComF, a protein encoded by the late competence operon; BioQ, a TetR-type transcription factor regulating biotin operon; BioJ, an isoenzyme of BioH demethylase. FabH, β-ketoacyl-ACP synthase III; FabG, β-ketoacyl-ACP reductase; FabZ, β-hydroxyacyl-ACP dehydratase; FabI, enoyl-ACP reductase; FAS II, type II fatty acid synthesis pathway. ACP, acyl carrier protein; M-pim-ACP, methyl pimeloyl-ACP; pim-ACP, pimeloyl-ACP; DTB, dethiobiotin.

**Fig 2:**
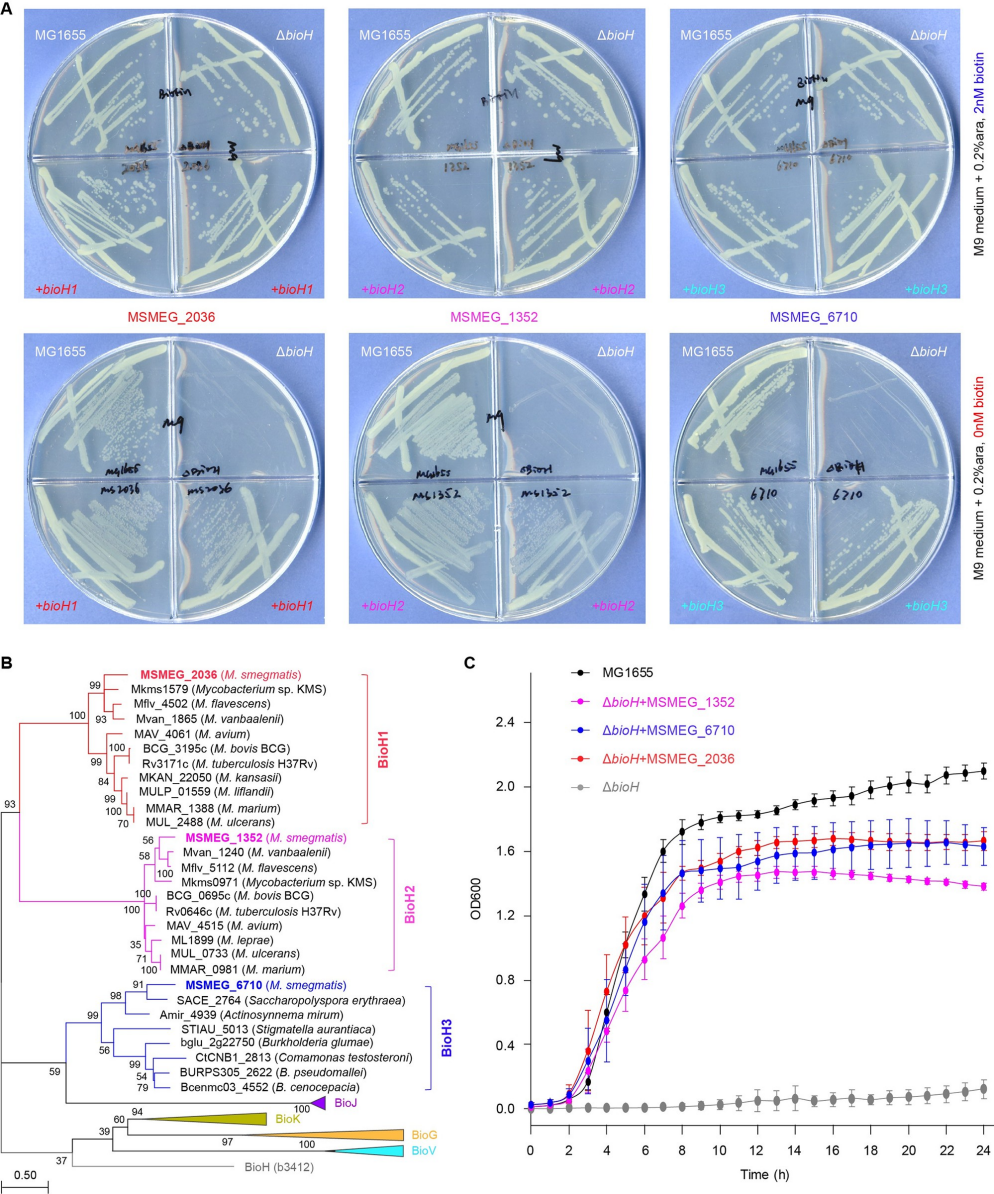
Expression cloning discovered three distinct *M*. *smegmatis* genes displaying BioH-like activities. **A.** *M*. *smegmatis* possesses three distinct genes whose expression consistently enables the recipient host of the *E*. *coli* Δ*bioH* biotin auxotrophic strain to appear on the non-permissive condition lacking biotin. A representative result of three independent experiments was given. Namely, the three BioH-like genes (designated BioH1 to BioH3) of *M*. *smegmatis* MC2 155 include MSMEG_2036, MSMEG_1352, and MSMEG_6710. **B.** Phylogeny of the three BioH isoenzymes (BioH1 to BioH3) from *M*. *smegmatis*. Except for BioH enzyme-including subclades, all the other four subtrees were compressed with triangles, which correspond to the previously-identified isoforms (BioJ, BioK, BioG and BioV). The majority of BioH phylogeny consists of three clusters. Namely, they include i) Subclade I, termed ‘BioH1’ (exemplified with MSMEG_2036); ii) Subclade II, labeled ‘BioH2’ (featured with MSMEG_1352); and iii) Subclade III, designated ‘BioH3’ (presented with MSMEG_6710). Number on the node denotes the bootstrap replicate. It seemed true that a large population of *Mycobacteria* contains both BioH1 and BioH2. In spite of its absence in the mycobacterial species other than *M*. *smegmatis*, BioH3 appears in other closely-relative cousins, like *Saccharopolyspora erythraea*, an erythromycin-producing actinomycete. The software of MEGA7 was applied in the generation of Maximum Likelihood (ML) tree. Jones-Taylor-Thornton (JTT) model was used, and the number of bootstrap replications is 1000. **C.** Use of growth curves to evaluate of the *E*. *coli* Δ*bioH* mutant expressing each of the three putative *bioH* genes (*bioH1* to *bioH3*). It was expressed in an average ± standard deviation (SD) from three independent experiments.

**Fig 3:**
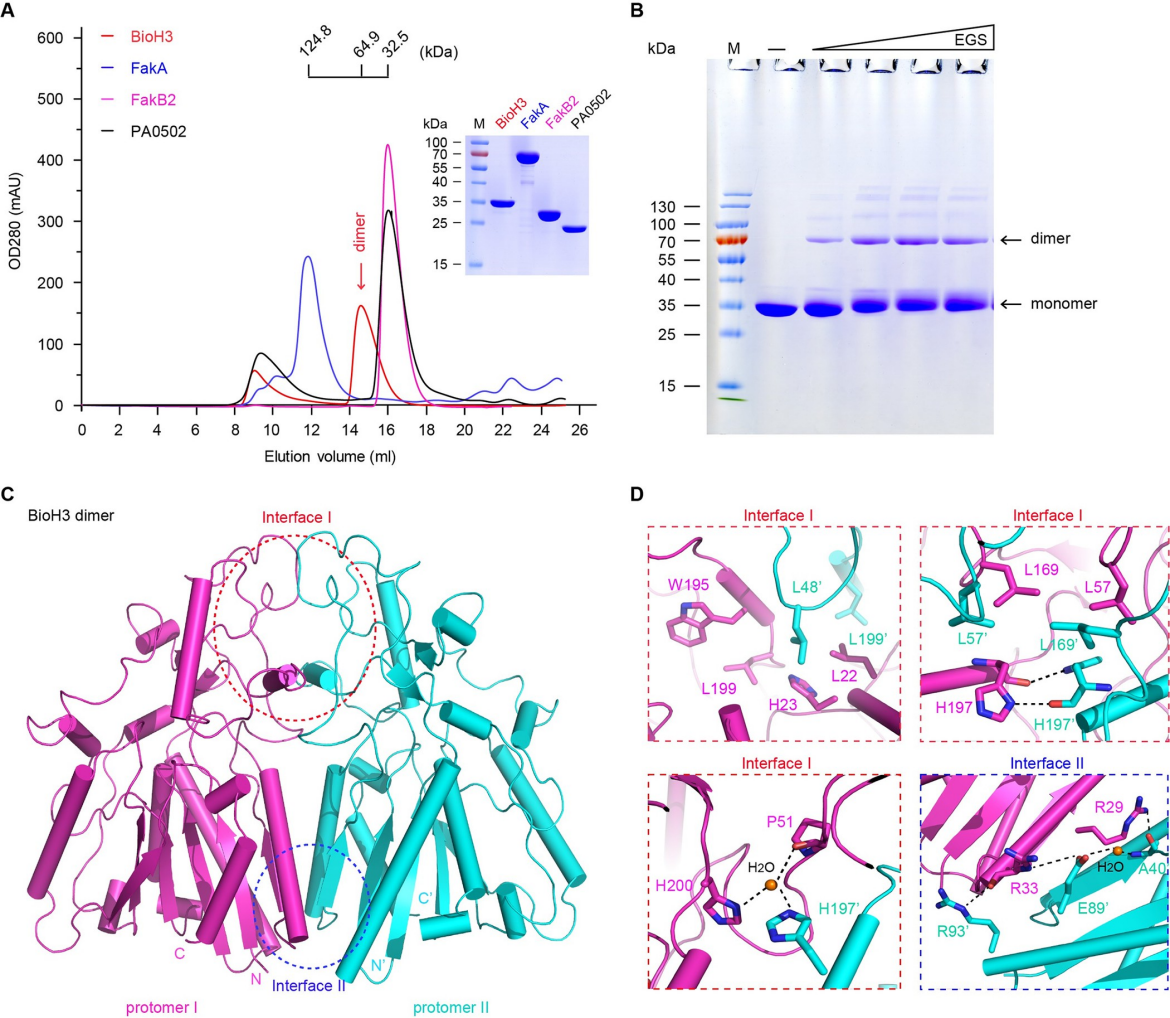
Integrated evidence that BioH3 is a dimer. **A.** Gel filtration profile suggests that solution structure of the purified BioH3 is a dimer. **B.** Chemical cross-linking analysis of the BioH3 protein. In addition to the monomeric band, dimeric band is given in the chemical cross-linking assay with the EGS cross-linker. **C.** Overall structure of dimeric BioH3. Architectural analysis of BioH3 suggests two binding-interfaces between protomer I and protomer II, namely Interface I and Interface II. **D.** Enlarged views of Interface I and Interface II. The critical residues involved in interface formation are labeled. The residues from protomer I are shown in magenta, and those from protomer II are given in cyan. Designations: mAU, milli absorbance units; kDa, kilo Dalton; EGS, ethylene glycol bis (succinimidyl succinate); FakA, fatty acid kinase A; FakB2, fatty acid kinase subunit B2; PA0502, a putative BioH enzyme from *Pseudomonas aeruginosa*; N, N-terminus; C, C-terminus.

**Fig 4:**
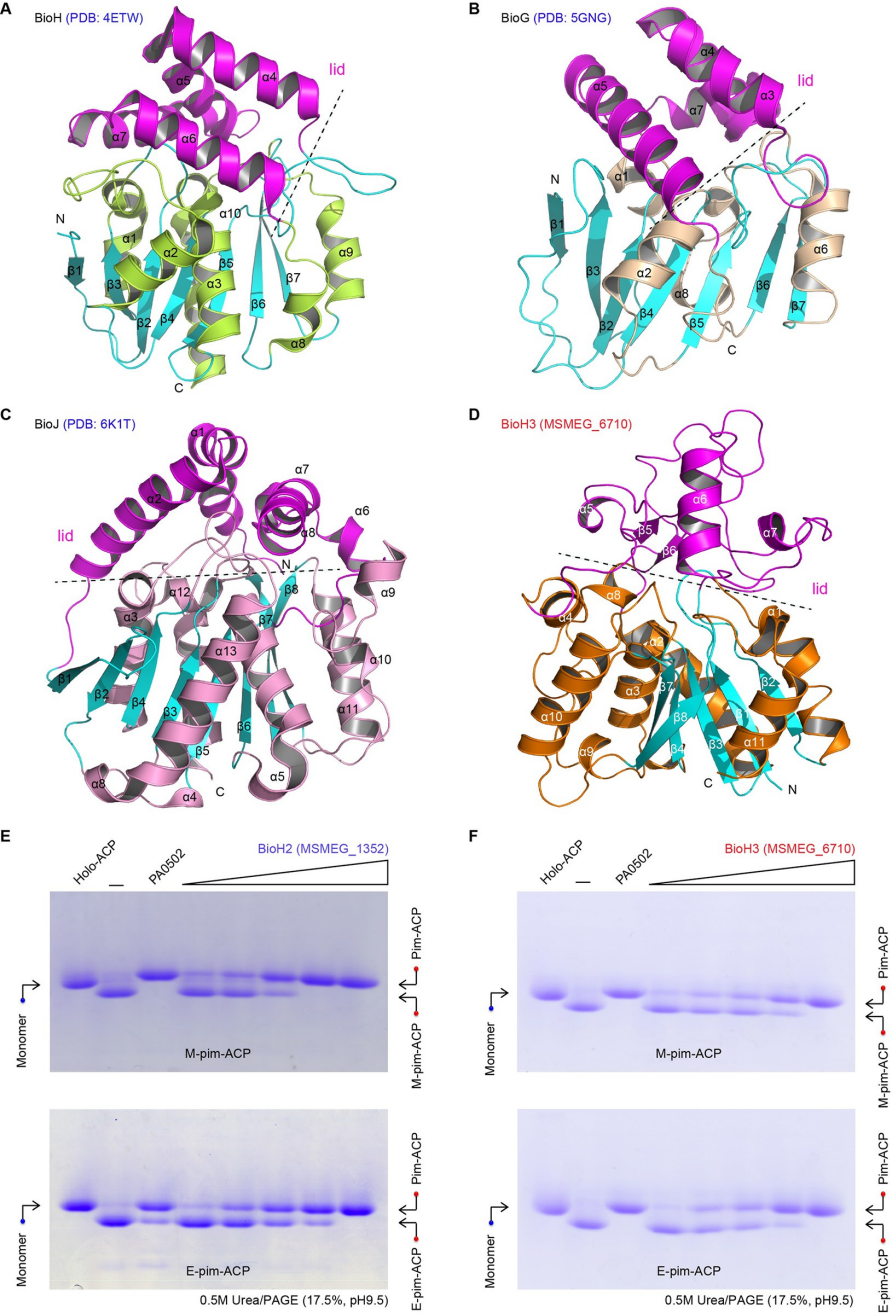
Functional and structural analysis of the two biochemically-amenable demethylases BioH2 and BioH3. Ribbon structures of three known biotin gatekeeper enzymes BioH (**A**), BioG (**B**), and BioJ (**C**). **D.** Ribbon presentation of the monomeric structure of BioH3 (MSMEG_6710). BioH3 seemed unusual in that its lid domain displays a unique folding mode compared with the other three known enzymes. **E.** BioH2 (MSMEG_1352) is enzymatically active, albeit of its aggregation in solution. **F.** BioH3 (MSMEG_6710) displays enzymatic activity of removing the methyl (ethyl) moiety from M-pim-ACP (E-pim-ACP) to give pim-ACP product. The enzymatic mechanism of BioH2 (and/or BioH3) action was examined *in vitro*, using its physiological substrate M-pim-ACP, as well as an alternative one E-pim-ACP. The reactant and product from BioH2 (BioH3) reaction were separated using conformationally-sensitive gel [0.5M urea/17.5% PAGE (pH9.5)]. In general, the reactant of M-pim-ACP (and/or E-pim-ACP) migrates faster than the product pim-ACP in such PAGE gel containing 0.5M urea. Designations: α, α-helices; β, β-sheet; N, N-terminus; C, C-terminus; ACP, Acyl carrier protein; M-pim-ACP, Methyl-pimeloyl-ACP; E-pim-ACP, Ethyl-pimeloyl-ACP; Pim-ACP, pimeloyl-ACP. The minus symbol “—” denotes no addition of any enzyme.

**Fig 5:**
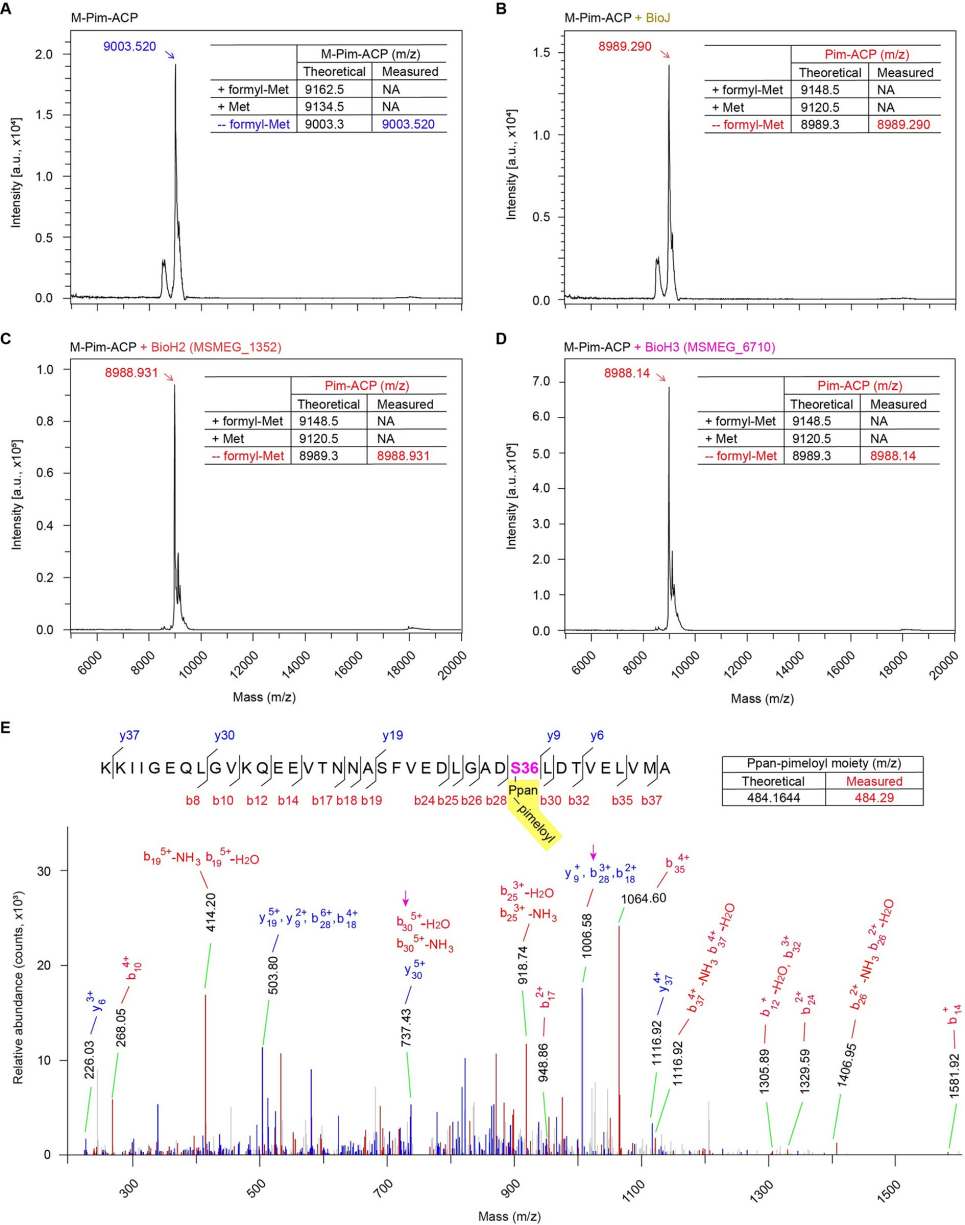
MS evidence for the enzymatic cleavage of the physiological substrate of pimeloyl ACP methyl ester by BioH2 and BioH3. **A.** MALDI-TOF profile for the substrate of pimeloyl-ACP methyl ester. **B.** MALDI-TOF identification of the pimeloyl-ACP product arising from BioJ hydrolysis of pimeloyl-ACP methyl ester. MALDI-TOF evidence that BioH2 (**C**) and BioH3 (**D**) both demethylate the reactant pimeloyl-ACP methyl thioester to give pimeloyl-ACP product. **E.** Use of high-resolution mass spectrometry to detect a pool of ACP peptide fragments of which the serine 36 (S36) features with a single pimeloyl modification. Designations: ACP, Acyl carrier protein; Pim-ACP, pimeloyl-ACP; M-pim-ACP, Methyl-pimeloyl-ACP; BioJ, *Fransicella* pimeloyl-ACP methyl ester carboxyl-esterase; NA, not available; Ppan, Phosphopantetheine.

The recent proposal of Tam (Trans-aconitate 2-methyltransferase) as BioC, the O-methyltransferase for malonyl-ACP, closes in on the first-stage of mycobacterial biotin synthesis [17]. Whereas its other partner demethylase, having BioH-like activity remains largely enigmatic. Similar to the paradigm organism *E*. *coli* and the plant pathogen *Xanthomonas* having a single pair of ‘*bioC-bioH*’, the human pathogen *Francisella* is also equipped with a *bioC* locus paired with *bioJ* alone, encoding the BioH isoenzyme (**Fig 1A**). Combined with expression cloning (**S1 Fig**), genomic mining suggested that three genetic loci (namely MSMEG_2036, MSMEG_1352, and MSMEG_6710) scattered on *M*. *smegmatis* MC^2^ 155 chromosome might produce BioH-like activities (**Fig 1A**). Compared to the prototype BioH of *E*. *coli* (EcBioH), they only exhibited the poor identity of 21.78–22.73% (**S2 Fig**). Thereafter, the three distinct isoenzymes were provisionally termed as BioH1, BioH2, and BioH3 (**Fig 1A**), of which genetic roles (**Fig 2**) and biochemical mechanisms (**Figs 3–5**) are discussed later. Among them, only two homologs (Rv3171c for BioH1 and Rv0646c for BioH2) are retained in the tuberculosis-causing cousin, *M*. *tuberculosis* (**Figs 1A** and **S3**). This is generally agrees with the proposal that bacterial genome is reduced from non-pathogenic species to pathogenic species, featuring with the loss of redundant genes [53]. To test if the three redundant *bioH* genes are actively transcribed, we performed the analysis of real-time quantitative PCR. As expected, all the three *bioH* isoforms (*bioH1* to *bioH3*) were transcribed at appreciable level (**Fig 1B**). Consistent with the well-described BioJ [40,41], the representative form of *M*. *smegmatis* BioH isoenzymes displayed the enzymatic activity of hydrolyzing the substrate of pimeloyl ACP methyl ester (M-pim-ACP) to give the product of pim-ACP *in vitro* (**Fig 1C**).

The discovery of varied BioH isoenzymes combined with BioC described by Hu and Cronan [17], enabled us to formulate the ‘BioC-BioH(n)’ pathway that essentially constitutes the earlier step for mycobacterial biotin synthesis (**Fig 1D**). Of note, the letter ‘n’ denotes the varied number of *bioH* genes (i.e., 2 for *M*. *tuberculosis*, and 3 for *M*. *smegmatis*). Unlike that AccB (Acetyl-CoA carboxylase subunit B) is a sole biotin-requiring protein (**Fig 1E**), *M*. *smegmatis* presumably encodes multiple protein subunits with the broadly-conserved motif (EAMKME), of which the lysine residue is modified with biotin by the biotin protein ligase (Bpl/BirA) (**Fig 1F**). This is generally consistent with streptavidin blot-based observations by Feng *et al*. [47] and Wei *et al*. [41]. It was noted that the physiological demand for biotin in *M*. *smegmatis* is around 16nM, much higher than that (~1nM) of *E*. *coli* (**Fig 1G**). Thus, it is possible that the unusual BioC-BioH(3) paradigm reflects certain evolutionary advantage in *M*. *smegmatis*, and guarantees the high efficiency in the *de novo* biotin production to satisfy its unique physiological requirement. However, this requires further experimental evidence.

### Three distinct BioH isoforms from *M*. *smegmatis*

In total, *M*. *smegmatis* MC^2^ 155 harbors 90 genes that are annotated as α/β-hydrolase members. They were collected to give an atlas of mycobacterial *bioH* candidates (**S2 Table**). The PCR products of all the 90 *bioH*-like genes were cloned into a low-copy expression vector pBAD322, giving a pool of expression clones (**S1 Fig**). Following the phenotype-to-function screen, three of them were found to enable the occurrence of the Δ*bioH* biotin auxotrophic strain on the non-permissive condition of M9 minimal agar plates lacking biotin (**Fig 2A**). This indicated that BioH-like activity of *M*. *smegmatis* can be attributed to three distinct genes sharing poor similarity (**S2 Fig**). For clarity and consistency, the three BioH isoenzymes were provisionally named BioH1 (MSMEG_2036), BioH2 (MSMEG_1352), and BioH3 (MSMEG_6710), respectively. In fact, GS11_3319 of *M*. *bovis* BCG, equivalent to BioH1 (MSMEG_2036) of *M*. *smegmatis*, was tested positive in supporting the Δ*bioH* mutant to appear on the non-permissive, biotin-deficient M9 minimal agar plates (**S3C** and **S3D Fig**). Not surprisingly, maximum-likelihood phylogeny showed that they are exactly positioned into three distinct subclades (**Fig 2B**). This somewhat verified the evolutionary diversity amongst BioH isoenzymes. Similar to those seen with M9 minimal medium agar plates (**Fig 2A**), the measurement of growth curves also demonstrated physiological roles of all the three BioH isoforms (**Fig 2C**). Of being noteworthy, unlike most mycobacterial species that only carry BioH1 and BioH2, the non-tuberculosis bacterium, *M*. *smegmatis* retains an additional one, BioH3 (**Fig 2B**). The observation agrees with the loss of redundant genes associated with bacterial transition from non-pathogen to pathogen [53]. Hence, this hints the possibility that BioH3 might be an ancestorial version in mycobacterial species.

### Characterization of three BioH isoenzymes

To characterize their biochemical properties, we overexpressed the three BioH isoforms (BioH1 to BioH3). Among them, BioH1 consistently behaves as inclusion body despite of different prokaryotic expression systems. The refolding approach also failed to recover soluble form of BioH1. This somewhat hampered us to test its enzymatic activity *in vitro*. In contrast, the recombinant forms of both BioH2 and BioH3 were purified to homogeneity. Different from BioH2 behaving as oligomer/soluble aggregates (**S4 Fig**), the purified BioH3 (~30kDa) was eluted at the position of around 60kDa, inferred as a dimer in our size exclusion chromatography with a Superdex 200 Increase column (**Fig 3A**). Chemical cross-linking analysis of BioH3 also revealed that the dimeric band is intensified with an increment of EGS cross-linker (**Fig 3B**). This constituted a biochemical proof that BioH3 appears as a dimer. Prior to this study, to the best of our knowledge, all the known BioH homologs and isoenzymes present monomeric structures, namely i) BioH of *E*. *coli* [54] and *Shigella* [31], ii) BioG of *Haemophilus* [39], and iii) BioJ from *Francisella* [40, 41]. However, the three BioH isoforms of *M*. *smegmatis* MC^2^ 155 described here, markedly differ in their solution structures, ranging from inclusion body (BioH1), oligomer (BioH2), to dimer (BioH3). Somewhat it is largely relevant to the fact that they are phylogenetically distributed in three distinct subclades (**Fig 2B**), despite that we are unaware of what the driving force underlying is thus far. Along with their genetic roles in complementing Δ*bioH* biotin auxotroph (**Fig 2A** and **2C**), the accumulated data benefited the anticipation that the BioH isoenzymes (BioH1 to BioH3) are evolutionarily diversified, but functionally unified. To further distinguish them, we attempted to perform a structure-to-function study of BioH isoforms.

### Crystal structure of BioH3 dimer

BioH3 (282aa) shares poor sequence similarity to its isoenzymes with known structures. The crystals of BioH3 were grown using the sitting-drop vapor diffusion method and diffracted up to 2.27-Å resolution (**Table 1**). The structure was determined using single-wavelength anomalous diffraction (SAD) method, in which the final R-work and R-free values are 0.19 and 0.22, respectively. The crystal belongs to the *P*2_1_ space group, and contains six protein molecules per asymmetric unit (ASU). Structurally, six copies of BioH3 are assembled into three homo-dimer, i.e., dimers AB, CD, and EF (PDB: 7WWF). The two BioH3 monomers contact each other via two interfaces (Interface I & Interface II), giving a 2-fold axis-centering dimeric architecture (**Fig 3C** and **3D**). To maintain the dimerization, three kinds of interactions are involved, namely i) hydrophobic interactions, ii) hydrogen bond (H-bond) networks, and iii) H_2_O-mediated H-bond interplay (**Fig 3D**). Apart from the side chain atoms, the main chain atoms of certain residues also participate in BioH3 dimerization. The fact that total buried surface area of the two interfaces is over 1760 Å^2^ underlined the stable existence of BioH3 dimer. It largely agrees with those of our gel filtration and chemical cross-linking (**Fig 3A** and **3B**).

**Table 1:** Data collection and refinement statistics.

Structure	Se-BioH3
PDB ID	7WWF
Data collection ^a^	
Space group	P2_1_
Cell parameter:	
*a*, *b*, *c* (Å)	91.7, 65.8, 172.8
α, β, γ (°)	90.0, 97.8, 90.0
Wavelength (Å)	0.9793
Resolution (Å)	90.8–2.27
Last shell (Å)	2.39–2.27
Completeness (%)	98.5 (100)
Redundancy	3.4 (3.6)
R_merge_ (%)	6.5 (47.9)
I/ σ(I)	11.9 (2.3)
Refinement	
Resolution (Å)	45.6–2.27
No. of reflections	93298
R_work_ (%) **/** R_free_ (%)	18.9/22.2
No. of atoms	
Protein	12372
PEG	28
Water	266
R.m.s. deviations	
Bond length (Å)	0.002
Bond angle (°)	0.579
Ramachandran plot (%)	
Most favorable	95.9
Additional allowed	4.1
Outlier	0.00

a: Values in parentheses are for the last resolution shell.

As a member of the α/β-hydrolase (i.e., esterase) family, they adopt an identical pattern of domain organization, i.e., N-terminal lid domain connected with a core domain at C-terminus (**Fig 4A–4C**). The paradigm BioH (256aa) of *E*. *coli* consists of an α-helical lid domain (α4–7) and a core catalytic domain [54], which denotes a central seven-stranded β-sheet surrounded with six α-helices (α1–3, α7 and α9–10) on both sides (**Fig 4A**). In contrast to BioH, the counterpart BioG (215aa) of *Haemophilus* markedly differs in its catalytic domain [39]. This is because the core β-sheets are neighbored with several long loops, rather than α-helices alone (**Fig 4B**). Different from BioH and BioG, the BioJ (306aa) restricted to *Francisella* adopted an atypical folding mode. In addition to its distinct lid/cap domain containing an extra α-helices, the BioJ core domain is featuring with a ‘sandwich’-like fold, i.e., a central eight (not seven) β-sheet flanked with three long α-helices on each side (**Fig 4C**). Not surprisingly, crystal structure of BioH3 (PDB: 7WWF) defines a unique member of α/β-hydrolase family. First, its core domain is of α/β-fold in nature, comprising of six parallel β-strands (β1–4 and β7–8), five α-helices (α1–3 and α10–11) and a short α9 turn (**Fig 4D**). In comparison with those of BioH, BioG and BioJ, the core domain of BioH3 were consistently scored to be around 2.0Å in RMSD (root mean square deviations) values. Structural superposition showed that the four core domains display a relatively-similar folding pattern. In contrast to its core domain, the lid domain of BioH3 is pretty unique. Apart from α-helices, the lid domain of BioH3 also contains two β-strands (β5–6) and several connecting linkers (**Fig 4D**). Except α6-helix, all other α-helices of BioH3 lid domain are very short. However, the lid domains of BioH, BioG, and BioJ are all formed by long α-helices and short loops (**Fig 4A–4C**). Therefore, we believed that the differentiated configurations of lids might explain in part, if not all, the varied placement of BioH isoforms in phylogeny (**Fig 2C**). Next, we integrated biochemical approaches to examine their activities *in vitro* and *in vivo*.

### Biochemical analyses of three BioH isoforms

Earlier works informed us that the prototype BioH of *E*. *coli* is a promiscuous demethylase capable of eliminating the methyl moiety from the substrate of M-pim-ACP (also called M-C7-ACP) to give pim-ACP (C7-ACP) product [23, 31]. Despite of the commercial unavailability of M-pim-ACP and ethyl pimeloyl-ACP (E-pim-ACP), the characterization of the versatile acyl-ACP synthetase (AasS) of *Vibrio harveyi* [55,56] allowed us to enzymatically synthesize them *in vitro*, as earlier described by Lin and coworkers (**S5A Fig**) [23]. Indeed, the promiscuous AasS enzyme transfers the non-natural fatty acid M-C7 to the acceptor holo-ACP, giving M-C7-ACP product that migrates faster than its reactant in conformationally-sensitive gel of 0.5M urea/ 17.5% PAGE (pH9.5) (**S5B Fig**). The identity of M-C7-ACP was essentially confirmed with mass spectrometry because that an ACP-originated peptide (DLGAD**S**LDTVELVMALEEEFDT) was detected to carry M-C7 modification at the residue S36 (**S5C Fig**). Then we established the *in vitro* enzymatic assays for various BioH isoforms to gain a biochemical proof of the principle (**Fig 1D**). As described by different groups with BioH [31] and BioJ [40,41], a conformationally-sensitive gel of 0.5M urea/17.5% PAGE (pH 9.5) was applied in the separation of C7-ACP product from its reactant M-C7-ACP (and/or its surrogate E-C7-ACP). This is because that such gel-based electrophoresis renders M-C7-ACP (E-C7-ACP) to migrate faster than its product C7-ACP (**Fig 1C**). Except for BioH1, an inclusion body protein, we are fortune to obtain the soluble BioH2 and BioH3 enabling biochemical amenability. Given the fact that i) FAS II path-relevant genomic context (**S6A** and **S6B Fig**), ii) sequence and structural similarity (**S6C–S6E Fig**), and iii) functional replacement/exchange of the paradigm AcpP of *E*. *coli* with AcpM of *Mycobacteria* in both AasS reaction (**S6F Fig**) and BioH action (**S6G Fig**), we preferentially adopted the EcACP cargo to load the unnatural fatty acid of M-pimelate (and E-pimelate) to make M-C7-ACP (E-C7-ACP), a substrate for BioH isoenzymes.

As expected, our enzymatic system reproduced that BioH (PA0502) of *Pseudomonas* as the positive control, can hydrolyze its physiological substrate M-pim-ACP, as well as its surrogate E-pim-ACP (**Fig 4E**). The oligomeric form of *M*. *smegmatis* BioH2 (MSMEG_1352) was found to cleave the methyl (or ethyl) moiety from M-pim-ACP (E-pim-ACP), producing pimeloyl-ACP in a dose-dependent manner (**Fig 4E**). Similarly, the *M*. *smegmatis* BioH3 dimer was also active in the liberation of methyl (ethyl) group from the M-C7-ACP (or its surrogate E-C7-ACP) substrate at comparable level (**Fig 4F**). To identify the product C7-ACP, we performed intensive analyses with two approaches namely i) Matrix-Assisted Laser Desorption Ionization (MALDI) Time of Flight (TOF), MALDI-TOF; and ii) Liquid Chromatography (LC) Mass Spectrometry, LC/MS. As shown in our MALDI-TOF profile, the reactant M-pim-ACP give a peak of 9003.520 m/z, almost identical to its theoretical mass of 9003.3 m/z (**Fig 5A**). By contrast, the positive control, BioJ-including reaction system displays a unique spectrum carrying a unique peak of 8989.290 m/z, corresponding to the pim-ACP product with theoretical mass of 8989.3 m/z (**Fig 5B**). Similar to a scenario with BioJ (**Figs 1C** and **5B**), the C7-ACP product-specific peaks were detected in the mixture from BioH reactions, namely i) the peak of 8988.931 m/z for BioH2 (**Fig 5C**), and ii) the one of 8988.14 m/z for BioH3 (**Fig 5D**). The LC/MS was used to analyze a pool of ACP-derived peptides of which the residue serine 36(S36) is modified with a pimeloyl moiety. Among them, an ACP peptide of interest enabled us to assign the calculated mass of 484.29 m/z to the prosthetic group of phosphopantetheine (Ppan) with C7 acylation (rather than M-C7 modification), of which the theoretical mass is 484.1644 (**Fig 5E**). The accumulated data confirmed that both BioH2 and BioH3 behave as active M-C7-ACP demethylases. Considering that i) they share similar genetic roles in *E*. *coli* (**Fig 2**) and ii) differentiation occurs in both solution structures (**Figs 3** and **S4**) and phylogeny (**Fig 2B**), integrated biochemical evidence allowed us to conclude that the three BioH isoforms (BioH1 to BioH3) play gatekeeping roles in the first-stage of mycobacterial biotin synthesis (**Fig 1D**).

### Parallels to catalytic triads of BioH isoforms

**Fig 6:**
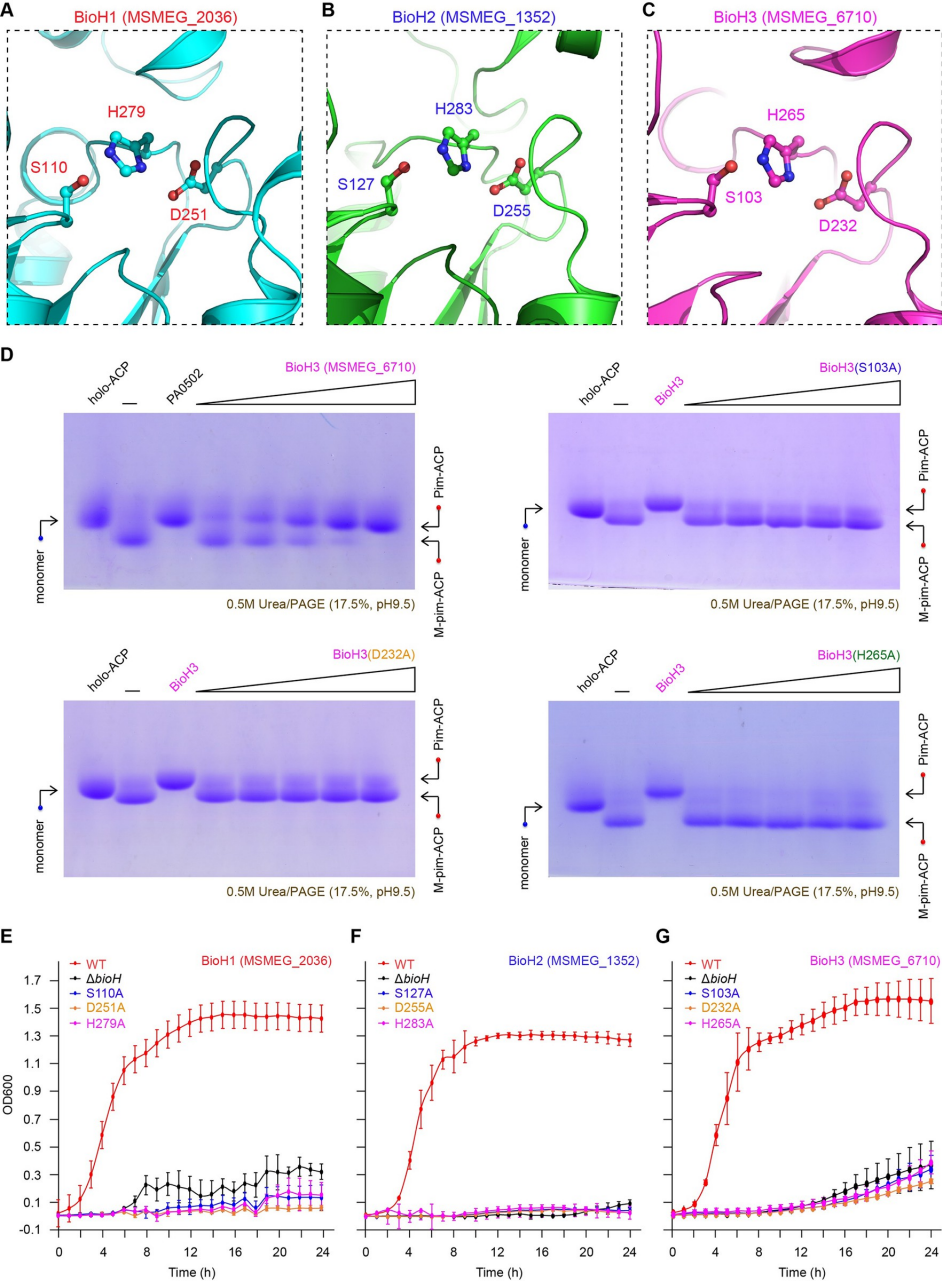
Structure-guided functional analysis for the conserved catalytic triad amongst the three BioH enzymes (BioH1 to BioH3). Structural snapshots of catalytic triads from *M*. *smegmatis* BioH1 (**A**) and BioH2 (**B**). Structures of both BioH1 and BioH2 were predicted with Alpha-fold. **C.** X-ray crystal structure-based visualization for the BioH3 (MSMEG_6710) catalytic triad. The three conserved residues from the catalytic triad correspond to i) S110, D251 & H279 (BioH1); ii) S127, D255 & H283 (BioH2); and iii) S103, D232 & H265 (BioH3). **D.** Use of enzymatic assays to evaluate the roles of catalytic triad (S103, D232, and H265) in BioH3 (MSMEG_6710) activity. Three alanine-substituted mutants of BioH3 created here, included S103A, D232A, and H265A, respectively. The *in vitro* enzymatic actions of BioH3 mutants were evaluated using conformationally-sensitive gel as described in **Fig 4**. **E.** The BioH1 mutant defective in its catalytic triad cannot restore bacterial growth of the *E*. *coli* Δ*bioH* biotin auxotrophic strain on the non-permissive condition lacking biotin. **F.** None of BioH2 mutants with the defection of catalytic triad enables the *E*. *coli* Δ*bioH* strain to appear on the biotin-deficient growth condition. **G.** The certain mutation of catalytic triad causes functional loss of BioH3. In total, 9 mutants of *M*. *smegmatis* BioH (BioH1 to BioH3) were engineered into the Δ*bioH* biotin auxotrophic strain. On the basis of biotin-lacking cultivation condition, growth curves were plotted to address the *in vivo* role of BioH (BioH1 to BioH3) catalytic triad. Three independent experiments were conducted, and final output was given in an average ± SD.

The activity of the paradigm BioH and its counterpart BioJ is well-known to rely on an evolutionarily-conserved catalytic triad. Like the counterpart (S82, D207, and H235) of *E*. *coli* BioH (**S2 Fig**), the catalytic center of BioJ is composed S151, D248, and H278, respectively [40, 41]. The sequence analysis of three BioH isoenzymes suggested the presence of three invariant catalytic triad-forming residues. Namely, they include i) S110, D251, and H279 for BioH1; ii) S127, D255, and H283 for BioH2; and iii) S103, D248, and H265 for BioH3 (**S2 Fig**). Also, AlphaFold2 prediction returned us two architecturally-similar catalytic triad of BioH1 and BioH2 (**Fig 6A** and **6B**) [57, 58], verifying the aforementioned sequence-based proposal (**S2 Fig**). However, crystal structure of BioH3 illustrated that only two predicted residues (S103 and H265) are localized in the catalytic triad. Instead of D248 predicted by sequence alignment, the residue D232 participates in the formation of catalytic triad (**Figs 6C** and **S2**). However, it cannot rule out the possibility that the ubiquitous residue D248 plays an alternative role in BioH3 action.

To functionally characterize these catalytic triads, we created a panel of single mutants of BioH isoenzymes (BioH1 to BioH3, **S7–S10 Figs**). We tested whether or not these mutants retain the abilities to complement the Δ*bioH* biotin auxotroph of *E*. *coli*. As predicted, none of the three BioH1 mutants with catalytic triad disrupted (S110A, D251A, and H279A), can restore the growth of the Δ*bioH* mutant on the biotin-deficient condition (**S8A Fig**). Also, the alanine substitution of catalytic triad largely impaired the activity of *bioH2* in the Δ*bioH* mutant of *E*. *coli* (**S8B Fig**). A similar result was obtained with BioH3 upon the catalytic triad was inactivated (**S8C Fig**). Apart from the wild-type of dimeric BioH3 (**S7A Fig**), we also over-expressed and purified its three mutants with defects in the catalytic triad, including S103A, D232A, and H265A, respectively (**S7B Fig**). The *in vitro* enzymatic assays confirmed that the wild-type of BioH3 is active in the hydrolysis of M-pim-ACP substrate, whereas no activity is detected in anyone of the three mutated versions, namely S103A, D232A, and H265A (**Fig 6D**). As expected, the use of growth curves facilitated us to validate the requirement of catalytic triads for all the three BioH isoenzymes (BioH1 to BioH3, **Fig 6E–6G**). In addition, we functionally defined the catalytic triad (S122, D263, and H291) of GS11_3319/BCG_3195c, a BioH1 homolog (57.4% identity) from the pathogenic bacterium, *M*. *bovis* BCG (**S9 Fig**). Despite that it is not involved in its catalytic triad, the D248 residue was examined to play a critical role in BioH3 action (**S10 Fig**). Consistent with those of BioH of *E*. *coli* [31] and BioJ of *Francisella* [40,41], these observations suggested parallels to catalytic triads amongst all the three BioH isoforms (**Figs 6** and **S7–S10**).

### Substrate recognition of M-C7-ACP by BioH3

**Fig 7:**
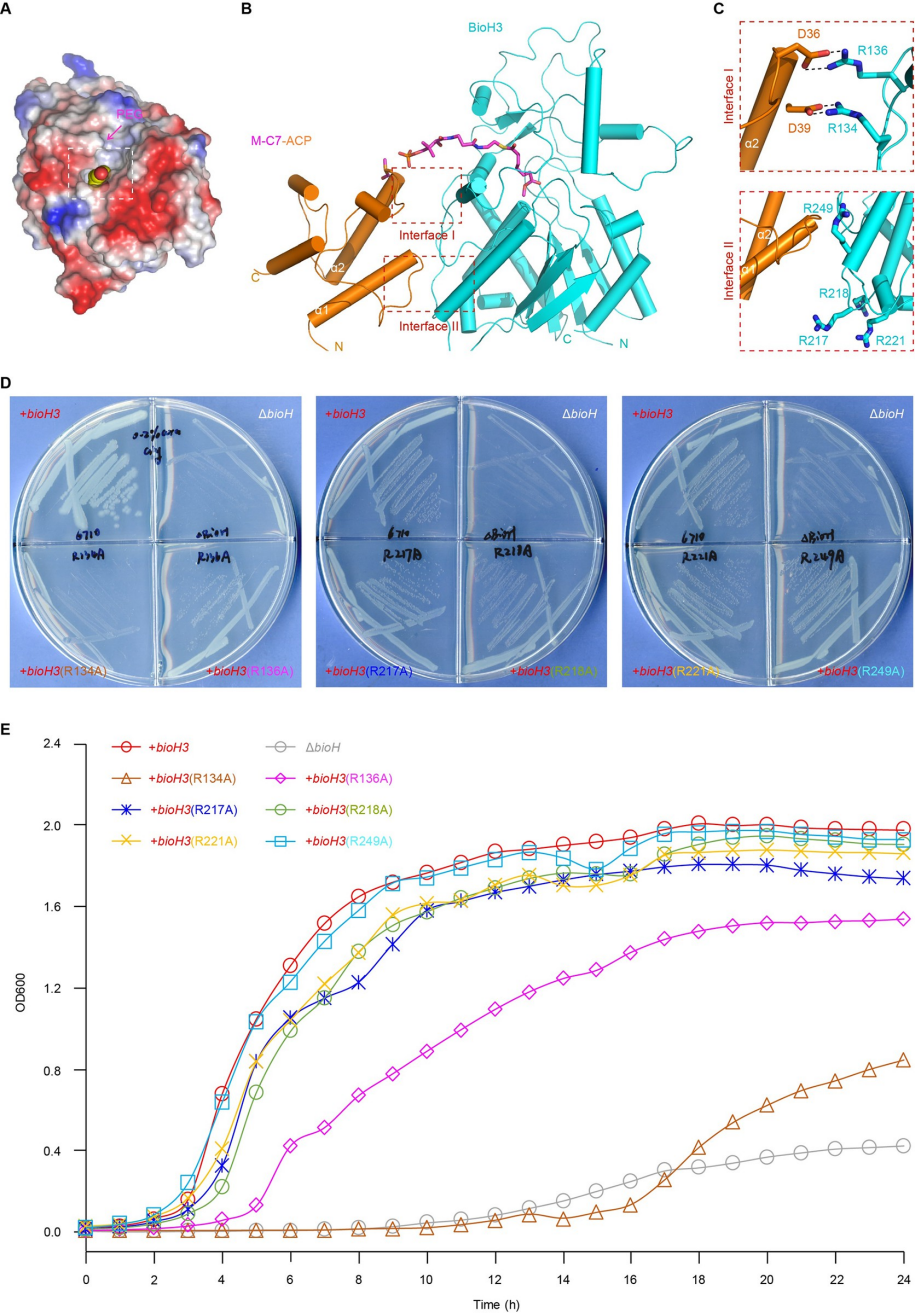
Structure-aided functional insights into recognition of the M-pim-ACP substrate by BioH3. **A.** Electrostatic surface illustration for BioH3 structure. A PEG-bound tunnel is detected, and highlighted with a dashed line rectangle. The 2F_o_-F_c_ electron density maps of PEG were contoured at 1.2δ level. It probably mimics the entry of physiological substrate M-pim-ACP into BioH3 enzyme. **B.** Structural presentation of BioH3 docked with the methyl pimeloyl moiety of M-C7-ACP. Presumably, the interplay of BioH3 with ACP comprises two interaction interfaces (highlighted with dashed line square), namely Interface I and Interface II. Both BioH3 and ACP are shown with cylinders. BioH3 is colored cyan, and ACP is displayed orange. Ppan-linked methyl pimeloyl moiety is given in sticks. **C.** Enlarged views of Interface I and Interface II engaged in BioH3-ACP interaction. As for Interface I, two electrostatic interactions are proposed. In brief, the two negatively-charged residues (D36 and D39) of ACP α2-helices pair with the two positively-charged amino acids (R136 and R134). Four positively-charged residues are suggested to involve in Interface II formation, namely R217, R218, R221, and R249. **D.** Use of site-directed mutagenesis to determine the contribution of interface I & II to BioH3 activity *in vivo1* **E.** Growth curves of the *E*. *coli* Δ*bioH* derivatives carrying an array of plasmid-borne *bioH3* mutants on the biotin-deficient, non-permissive condition. A panel of *bioH3* mutants are cloned into pBAD322, transformed into the *E*. *coli* Δ*bioH* biotin auxotrophic strain, and functionally assayed on the basis of bacterial growth on the non-permissive M9 medium lacking biotin. 0.1% arabinose (0.1% ara) was supplemented to induce expression of *bioH3* (its mutants). Namely, six single mutants of *bioH3* included R134A, R136A, R217, R218, R221, and R249. Two of six residues (R134 and R136) are found to play major roles in BioH3 activity. A representative result from three independent experiments was given. Designations: α, α-helices; N, N-terminus; C, C-terminus; ACP, Acyl carrier protein; M-C7-ACP, Methyl-pimeloyl-ACP.

The loading and delivery of the unusual M-C7 fatty acid by ACP cargo is prerequisite for the action of BioH3 demethylase. Folding of the core catalytic domains of BioH3 and the homologs is quite similar (**S11A Fig**). To seek how BioH3 communicates with its partner M-C7-ACP, we aimed to crystalize BioH3 in complex with the substrate of M-C7-ACP. Despite that our continued efforts failed to obtain the crystal of BioH3/M-C7-ACP complex, one well-defined PEG molecule was observed in the apo-form of BioH3 containing (**Figs 7A** and **S11B**). Similar to the *Shigella* BioH with known complex structure (**S12 Fig**) [31], the PEG-loading cavity of BioH3 are mainly formed by the side chains of hydrophobic residues (e.g., W18, F137, and F183) from its core domain (**S11B Fig**). Moreover, structural superposition revealed that four of the conserved cavity residues exhibit markedly-different conformation between BioH3 and the paradigm BioH (**S11C Fig**). Given that PEG mimics fatty acyl chain of the M-C7-ACP substrate, this PEG-occupied cavity probably resembles the physiological substrate-loading tunnel (**Fig 7A**). Despite that their substrate cavity main bodies are orientationally paralleled (**Figs 7B** and **S12B**), the composition and conformation of the M-C7-ACP cavity gates differ dramatically between BioH3 and its prototype of *E*. *coli* [54] and *Shigella* (**S13 Fig**) [31]. As illustrated by Agarwal and coworkers [31], the cavity gate of BioH is majorly formed by the α4 and α5 helices (**S13A Fig**). Whereas, the equivalent gate in BioH3 might be constituted of α4 and the α4-α5 linker (**S13B** and **S13C Fig**). In addition to hydrophobic residues, the BioH3 cavity gate also contains several Proline residues, which are rigid in conformation (**S13B Fig**). The substrate cavity gates are well defined in all the six BioH3 molecules in the structure, supported by their clear 2F_o_-F_c_ electron density maps. Also, the large buried surface area (>1200Å^2^) between the gate and other residues of BioH3 verified the conformational stability of this gate.

Structural superposition showed that the α4-α5 linker of BioH3 resides in the middle of BioH cavity gate, suggesting that the substrate cannot enter the cavity from the same location (**S13C Fig**). It seemed likely that the M-C7-ACP substrate might enter the cavity from the middle of the gate in the BioH3 structure. As we knew with the paradigm BioH for years, the carrier protein ACP mainly interacts with BioH via electrostatic interactions (**S12A–S1C Fig**) [31]. Presumably, the four positively-charged Arginine residues (R138, R142, R155, and R159) of BioH form ionic bonds with the six negatively-charged residues (Q14, D35, D38, E47, I54, and D56) of ACP α2-helices (**S12C Fig**), posing a synergistic role in the substrate binding and catalysis of BioH [31]. BioH3 also contains two Arg residues, R180 and R181, near the ACP binding site in the BioH/M-C7-ACP structure, but mutation of either R180 or R181 has no strong impacts on the function of BioH3. To identify the residues important for substrate binding by BioH3, we performed intensive structural analyses, and found one additional arginine-rich region close to the cavity gate, of which the basic residues include R134, R136, R217, R218, R221, and R249 (**Figs 7C** and **S13D**). These positively-charged residues are supposed to interact with certain acidic residues (e.g.: D34 and D36) of ACP α2-helices (**Fig 7C**). To investigate whether these Arg residues contribute to substrate binding and catalysis, we constructed all the six *bioH3* mutants and carried out the *in vivo* assays (**Fig 7D** and **7E**). As a result, two of the six single mutants (R134A and R136A) were showed to largely lose the ability of allowing the growth of the *E*. *coli* Δ*bioH* biotin auxotroph on the non-permissive, chemically-defined M9 media lacking biotin (**Fig 7D**). A similar scenario was also reproduced when we measured growth curves in the context of the *E*. *coli* Δ*bioH* mutants (**Fig 7E**). Probably, the remaining four arginine residues (R217, R218, R221, and R249) act in a synergistic manner, not alone (**Fig 7B**). Collectively, the structure-guided functional study enabled the proposal for a plausible explanation of how the cargo ACP recognizes BioH3 enzyme for the delivery of M-C7 fatty acids into the substrate cavity.

### Physiological roles of three BioH isoforms

**Fig 8:**
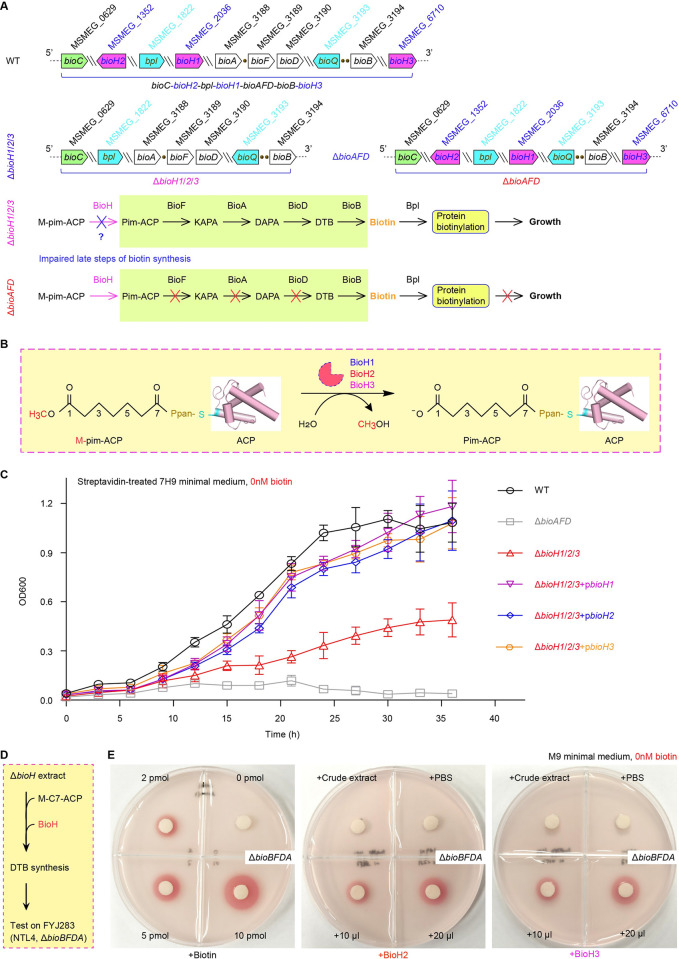
Genetic and biochemical evidence that three BioH isoenzymes are dedicated to biotin synthesis. **A.** Schematic illustration for genetic context of the triple *bioH* mutant (Δ*bioH1*/*2*/*3*) of *M*. *smegmatis*, a biotin auxotrophic strain. **B.** Chemical reaction for the three BioH isoforms that cleave the M-pim-ACP to produce pim-ACP. **C.** An individual *bioH* gene (*bioH1* to *bioH3*) can significantly restore the viability of the triple mutant of Δ*bioH1*/*2*/*3* on the biotin-deficient growth condition. The MC2 155 strain (WT) is the positive control, and the biotin auxotrophic strain of *M*. *smegmatis* (Δ*bioAFD*) lacking the late step of biotin synthesis, is the negative control. The triple mutant of *bioH* is denoted with Δ*bioH1*/*2*/*3*, and its complementary strain carrying plasmid-borne *bioH* is labeled with Δ*bioH1*/*2*/*3+*p*bioH* (H1 to H3). **D.** Schematic representation of the *in vitro* reconstitution of DTB synthesis. **E.** The purified form of BioH2 (and/or BioH3) protein reconstitutes DTB/biotin synthesis *in vitro*. The biotin auxotrophic strain FYJ283 of *A*. *tumefaciens* NTL4 (Δ*bioBFDA*) we earlier developed [47], acted as an indicator bacterium as described recently by Zhang *et al*. [37]. Designations: M-pim-ACP, Methyl pimeloyl-ACP ester; Bpl, Biotin protein ligase; M-C7-ACP, Pimeloyl-ACP methyl ester.

Since mycobacterial biotin metabolism is an attractive anti-TB drug target [11,13,14], it is reasonable to ask the question of whether and how the individual *bioH* gene gatekeeps the formation of pimeloyl-ACP, the *bona fide* precursor for the biotin cofactor, which in turn determines bacterial viability in *M*. *smegmatis* (**Fig 8A** and **8B**). Using the method of homologous recombination, we constructed an array of *bioH* mutants of *M*. *smegmatis*, as well as the genetically-complemented strains (**S3 Table**). Namely, these mutants include 3 single mutants (Δ*bioH1*, Δ*bioH2*, and Δ*bioH3*), 3 double mutants (Δ*bioH1*/*2*, Δ*bioH2*/*3*, and Δ*bioH1*/*3*), and 1 triple mutant (**S14A–S14C Fig**). Each of the three *bioH* genes (*bioH1* to *bioH3*) was re-introduced into Δ*bioH1*/2/*3*, the biotin auxotroph, yielding three complementary strains, namely i) Δ*bioH1*/2/*3*+p*bioH1*, ii) Δ*bioH1*/2/*3*+p*bioH2*, and iii) Δ*bioH1*/2/*3*+p*bioH3* (**Fig 8C**). Additionally, we generated a control strain of Δ*bioAFD*, in which most of the late step of biotin synthesis is eliminated (**Fig 8A**). Three BioH isoforms are programed into an early stage of biotin synthesis, arguing the redundancy of lipid metabolism (**Fig 8B**). This is unusual, but not without any precedent, because that i) two annotated *bioF* isoforms (Rv1569 for *bioF1*, and Rv0036 for *bioF2*) occur in *M*. *tuberculosis* H37Rv (**Fig 1A**), and ii) two *bpl*/*birA* homologs (FTN_0568 for *birA*, and FTN_0811 for *bplA*) also appear in *Francisella* [59]. Probably, it arises as an adaptation to the fluctuation of scarce nutrient at its ancestry stage.

Bacterial viability assays with the 7H9 (and/or 7H10) chemically-defined media confirmed that the control strain (i.e., the Δ*bioAFD* mutant of *M*. *smegmatis*) cannot appear unless the addition of 16nM biotin (**S14 Fig**). This validated that the non-permissive growth condition we established lacks the detective level of biotin. Not surprisingly, a single mutant of *M*. *smegmatis* (Δ*bioH1* to Δ*bioH3*) retained an ability to grow robustly on 7H10 agar plates regardless of biotin (**S14A** and **S14B Fig**). Similar scenarios were consistently seen with all the double mutants, namely Δ*bioH1*/*2*, Δ*bioH2*/*3*, and Δ*bioH1*/*3* (**S14B Fig**). Of note, the MC^2^ 155 strain of *M*. *smegmatis* exhibited poor growth upon the removal of all the three *bioH* isoforms, and such growth defect of the triple mutant (Δ*bioH1*/*2*/*3*) was largely restored, when complemented with certain *bioH* gene (*bioH1* to *bioH3*) (**Fig 8C**) or supplemented with 16nM biotin (**S14C Fig**). The genetic manipulations revealed that the three *bioH* isoforms (*bioH1*, *bioH2*, and *bioH3*) are involved in mycobacterial biotin synthesis, prerequisite for bacterial viability. However, we lacked evidence to figure out which *bioH* locus is physiologically dominant thus far.

Next, we examined a role of BioH in the *in vitro* reconstituted biotin synthesis system (**Fig 8D**). As established by Zhang *et al*. [37] with BioZ/BioJ action, the biotin auxotroph of *A*. *tumefaciens* (Δ*bioBFDA*) was mixed appropriately into agar plates, and functioned as an indicator strain in the presence of 2,3,5-triphenyl tetrazolium chloride (TTC, 0.001%). In principle, i) the indicator strain displays growth circle in red, supposing biotin is supplied or produced *in vitro* in our reconstituted system; and ii) bacterial respiration, a trait of viability, reduces TTC to release an insoluble red pigment precipitated around viable cells [40]. Thus, the supply of biotin here is judged according to the appearance of red pigment-decorating growth circle. First, as for the positive control, biotin added up to 2pmol, can support the growth of the indicator strain (**Fig 8E**). Second, neither the crude extract of *E*. *coli* Δ*bioH* mutant nor 1x PBS buffer (the components used for this *in vitro* reconstituted biotin system) allows the occurrence of growth circle in red, indicting the absence of contaminated biotin (**Fig 8E**). However, upon the supplementation of either BioH2 or BioH3 isoenzymes, the mixture (10–20μl) of the *in vitro* reaction system spotted on the paper disc was sufficient to enable the robust growth of the Δ*bioBFDA* biotin auxotroph on the non-permissive, biotin-deficient 7H10 agar plates, in parallels to that of 2-5pmol biotin (**Fig 8E**). This biotin bioassay provided biochemical evidence that BioH2 and BioH3 (except BioH1 biochemically intractable, due to inclusion body) are programed into the first-stage of biotin synthesis, essential for mycobacterial viability.

## Conclusions

As part of the group B vitamins (B7), biotin functions as a prevalent covalently-attached enzyme cofactor widely distributed across the three domains of life [22,29]. Although it has been discovered for almost 100 years [60], the diversity in *de novo* biotin biosynthesis pathway remains known incompletely. Apart from its extensive roles in central metabolism of carbohydrates, amino acids, and fatty acids [22,29], biotin metabolism is implicated in successful infections of certain pathogens [14,61]. In addition to two notorious agents, *Francisella* [40,59,62] and *Mycobacteria* [11,16,63], these examples already been extended to all the gram-negative members of ESKAPE (*E**nterococcus faecium*, *S**taphylococcus aureus*, *K**lebsiella pneumoniae*, *A**cinetobacter baumannii*, *P**seudomonas aeruginosa*, and *E**nterobacter* species) pathogens [61]. It underlined the importance of biotin as a limited/nutritional virulence factor. Thus, it is not surprising that biotin metabolism can be developed into a promising anti-TB drug target [13, 64]. However, there is an obstacle to expanding the arsenal of lead compounds/inhibitors against biotin paths because of the limited understanding the first-stage of mycobacterial biotin synthesis. The data reported here closes in on mycobacterial biotin synthesis. To the best of our knowledge, the discovery of three enigmatic BioH isoforms, combined with BioC renamed from Tam by Zhu and Cronan [17], represents an unusual paradigm ‘BioC-BioH(3)’ for the early stage of biotin synthesis in *M*. *smegmatis* (**Fig 1**). In this case, three distinct BioH isoenzymes are programmed into the removal of methyl moiety from M-C7-ACP, giving C7-ACP, the cognate precursor for biotin biosynthesis of *M*. *smegmatis*. Of note, its paralleled model denote ‘BioC-BioH(2)’ path in the TB causative, *M*. *tuberculosis* having two functional BioH isoforms (Rv3171c for BioH1 and Rv0646 for BioH2). Of note, this work corrected inappropriate assignment of MMAR_1997/Rv2715 as BioH earlier by different groups [9,13,65], into MMAR_1388 (Rv3171c) for BioH1 and MMAR_0981 (Rv0646) for BioH2 (**Fig 2B**). The reduced number of BioH from 3 to 2, is probably related with genome reduction along with the transition of bacterial virulence from the non-TB agent, *M*. *smegmatis*, to TB-causing bacterium, *M*. *tuberculosis* (**Fig 1**) [53]. Somewhat it also agrees in part, if not all, with the situation that the loss of BioQ, a TetR-type transcription factor functioning in *M*. *smegmatis*, occurs in *M*. *tuberculosis* [66,67].

As one of facultative/intracellular pathogens, *M*. *tuberculosis* is dependent on the impermeable architecture of its unique cell wall consisting of mycolic acids, very long-chain fatty acids [68]. Apart from the ACP cargo-dependent FAS II system comprising a set of discrete monofunctional enzymes, complexity in mycolic acid synthesis is also attributed to the requirement of a eukaryotic-like FAS I multifunctional enzyme [68]. As a result, mycobacterial cell wall contains up to 60% of lipids, whose abundance unusually accounts for up to 40% of dry weight of mycobacterial cells. A large number of auxiliary genes which are borrowed and even domesticated, are necessary for mycobacterial physiology. That might constitute an explanation for the unexpected redundancy in O-methyltransferases (72) and α/β-hydrolases (90) (**S1** and **S2 Tables**). Different from the newly-proposed Tam/BioC having relatively-low activity, the three BioH isoenzymes possess appreciable levels of catalysis *in vitro* and *in vivo*, albeit with their evolutionary divergence (**Figs 2** and **4–6**). Unlike the *bioAFD* cluster, three of the 4 late step-encoding genes, all the three *bioH* isoforms, namely *bioH1* (MSMEG_2036), *bioH2* (MSMEG_1352), and *bioH3* (MSMEG_6710), are free-standing loci (**Fig 1A**). It should be noted that MSMEG_2036 has been predicted by Wei *et al*. [66], but lacks experimental proof. A similar scenario was observed in the Tam/BioC (MSMEG_0629) scattered on the chromosome. In spite that both *bioFD* and *bioB* are modestly regulated by the BioQ repressor [66,67], neither the *bioH* isoform nor *bioC* has the canonical BioQ-binding sites (**Fig 1A**). Not only does the advantage of uncontrolled/loose expression of genes ‘*bioC*-*bioH*(3)’ probably assure sufficient enzymes engaged in the first-stage of mycobacterial biotin synthesis, but also provides promiscuous activities other than biotin metabolism. Indeed, such regulation by BioQ is present in most of the non-TB mycobacterial species, whereas not in the TB-causing bacterium, *M*. *tuberculosis* [67]. It seems most likely that the absence of BioQ regulation guarantees efficient production of biotin in the pathogenic *M*. *tuberculosis* with more biotin requirement, which in turn benefits its phagosomal escape and survival within harsh host environment.

We are aware that *B*. *subtilis* employs two redundant members, BioI [69,70] and BioW [46,70], to produce pimeloyl thioester destinated to biotin synthesis. In contrast to BioI, the cytochrome P450-like fatty acid scissor, the pimeloyl-CoA synthetase BioW is essential for bacterial viability [45]. This hints that BioW rather than BioI dominates in biotin synthesis. However, none of single or double mutants of *bioH* isoforms (3 in total) are biotin auxotrophic, suggesting indistinguishable gatekeeping roles in formation of biotin precursor, pimeloyl group (**Fig 8**). So far, phenotypic screening combined with fragment-based drug discovery returns numbers of hits in the context of mycobacterial biotin metabolism [13]. They include BioA [71–73], BioF [13,14], and Bpl [11,16], respectively. Among them, most of the anti-TB inhibitors arise from extensive studies with BioA of *Mycobacteria* [13]. It was noted that mycobacterial Bpl-aided biotin modification is becoming a prime frontline anti-TB drug target, even though it lacks a regulatory role in comparison with the bifunctional Bpl/BirA regulator [11,16]. This is because that genetic silencing of Bpl efficiently eliminates acute and chronic TB infections in mice [11]. By contrast, lead compounds targeting the first-stage of biotin synthesis are scarcely selected at all. This is explained in part by the lack of understanding early stage of mycobacterial biotin synthesis. In light that genetic inactivation results in the loss of bacterial viability reported in this study (**Figs 8** and **S14**), three BioH isoforms probably contribute to mechanism-based anti-TB drug discovery. Prior to this study, high-resolution structures were restricted to BioA [74,75] and Bpl [76], in the context of mycobacterial biotin metabolism. The atypical folding of BioH3 as a dimer, not a monomer as the prototype BioH of *E*. *coli* does (**Fig 3**), is probably due to the requirement of protein stability. Presumably, dimeric structure of BioH3 covers and/or disguises its vulnerable parts to bypass the degradation by certain proteasome. The inability of harvesting its co-crystal with M-C7-ACP, is probably due to the transient interaction of BioH3 with its substrate. This is prevalent in that most of the FAS II enzymes, like FabA [77] and FabD [78], are crosslinked with ACP cargo, prior to protein crystallization screen. Despite that BioH1 in the form of inclusion body compromises biochemical efforts, the oligomeric form of BioH2 deserves further exploration using the Cryo-EM technology (**S4 Fig**). The availability of BioH3 structure furthers mechanistic understanding mycobacterial biotin biosynthesis pathway.

In summary, this study defined three distinct BioH isoenzymes programmed in the early stage of mycobacterial biotin biosynthesis, an attractive anti-TB drug target. Unlike that its late step is long settled, the first-stage of mycobacterial biotin synthesis is a long-standing puzzle. Together with Tam/BioC assignment of Cronan’s group [17], characterization of BioH isoforms established a paradigm of ‘BioC-BioH(n)’ path for an early stage of mycobacterial biotin metabolism (n, 2 for TB bug and 3 for non-TB cousin). This finding closes in on complete biotin synthesis pathways, providing biochemical basis for the next-generation of anti-TB drug discovery targeting mycobacterial biotin nutritional immunity.

## Materials and methods

### Bacterial strains and growth conditions

The bacterial strains used in this study included *Escherichia coli* (*E*. *coli*), *Agrobacterium tumefaciens* (*A*. *tumefaciens*), and *Mycobacterium smegmatis* (*M*. *smegmatis*). All the *E*. *coli* strains arising from K-12 MG1655, were kept in either Luria-Bertani (LB) or M9 minimal medium (**S3 Table**). Both LB and M9 minimal media can supported the growth of *A*. *tumefaciens*. Apart from the maintenance with LB medium, the derivatives of *M*. *smegmatis* MC^2^ 155 were grown in 7H9 minimal medium (Becton, Dickinson and Company; USA) containing 0.2% glycerol and 0.05% Tween 80, as earlier described [66, 67]. Appropriate level of biotin was required for the viability of several biotin auxotrophic strains, namely i) ER90, the Δ*bioFCD* mutant of *E*. *coli* [23]; ii) FYJ283, the Δ*bioBFDA* mutant of *A*. *tumefaciens* [47]; and iii) FYJ5333, the Δ*bioAFD* mutant of *M*. *smegmatis* (**S3 Table**). It was noted that the Δ*bioAFD* mutant of *M*. *smegmatis* was given via an introduction of a pMind-based knockout plasmid carrying two homologous regions (~1kb) amplified with a pair of specific primers (**S4 Table**). Unlike *A*. *tumefaciens* that routinely appeared at 28°C [47], both *E*. *coli* and *M*. *smegmatis* were cultivated at 37°C [79]. DH5α was used for gene cloning, and BL21(DE3) was applied in protein expression. Strain STL24, the Δ*bioH* mutant of *E*. *coli* [23], acted as a recipient host for functional evaluation of *bioH* isoforms and the mutants on the non-permissive condition of M9 minimal medium lacking biotin [37,40].

### Plasmids and molecular manipulations

The genome-wide mining of *M*. *smegmatis* MC^2^ 155 was conducted, giving two atlases of *bioC*-like O methyl-transferases (**S1 Table**) and *bioH* candidates, carboxylesterases (**S2 Table**). First, all the 90 putative *bioH* genes were PCR amplified with 90 pairs of specific primers containing homologous arms (**S4 Table**), and gel purified prior to the PCR cloning. In the presence of NovoRec plus recombinase provided by NovoRec plus One step PCR cloning kit (NovoProtein, Shanghai, China), the resultant PCR product (~60ng) was mixed at the ratio of 3:1 with the linear form of pBAD322 vector (~20ng) in the 20μl reaction system, and kept at 50°C for 15min. The 5μl of reaction mixture was transformed into DH5α cells, yielding a pool of expression clones (**S3 Table**). The positive hits were determined via genetic complementation of the host strain STL24 (*E*. *coli* Δ*bioH* mutant, **S3 Table**) on the non-permissive condition of M9 minimal agar media without any biotin. Following overnight maintenance of the *E*. *coli* Δ*bioH* transformants at 37°C, three *bioH* candidates (named *bioH1* to *bioH3*) were screened, corresponding to MSMEG_2036, MSMEG_1352, and MSMEG_6710, respectively. Whereas only two *bioH* homologs were detected in the BCG strain of *M*. *tuberculosis* (**S2 Table**), namely BCG_3195c (54.7% identity with MSMEG_2036, *bioH1*), and BCG_0695c (64.6% identity with MSMEG_1352, *bioH2*). In contrast, the most similar one, BCG_2728 displayed only 14.9% identity with MSMEG_6710 (*bioH3*). Thus, they also were genetically amenable to functional assays. To extend biochemical assays, a panel of engineering *E*. *coli* strains were developed for recombinant protein production, which harbor various pET28-borne *bioH* expression plasmids (**S3** and **S4 Tables**). Site-directed mutagenesis was applied to create all the mutants of *bioH* catalytic triads, as well as the ACP-interacting sites (**S3 Table**). All the resultant recombinant plasmids were confirmed by Sanger sequencing.

### RNA isolation, RT-PCR and real-time qPCR

The strain MC^2^ 155 of *M*. *smegmatis* was grown in 7H9 minimal medium supplemented with 0.2% glycerol and 0.05% Tween 80, and the mid-log phase cultures at an OD_600_ of 1.2, were harvested for total RNA isolation. The RNApure kit (ZOMANBIO, China) was used to extract the pool of bacterial RNA species as recommended by the manufacturer. The residual DNA was digested with DNase I (TakaRa, Japan). As described earlier with FadH, the 2,4-dienoyl reductase involved in β-oxidation of bacterial unsaturated fatty acids [80], bacterial RNA quality was judged by electrophoresis on 1.0% agarose gel, and the possible contamination of genomic DNA in the RNA samples was examined by the PCR detection using the pair of *16S rRNA*-specific primers (**S4 Table**), along with the total RNA as the template. Then, the qualified RNA preparations were subjected to synthesis of the first-strand complementary DNA (cDNA) by reverse transcription (RT). To check if the three *bioH* isoforms (*bioH1* to *bioH3*) we proposed, are actively transcribed or not, the routine PCR were conducted using three pairs of unique primers (**S4 Table**), together with the above cDNA as the template. In brief, the PCR reaction mixture (20μl) included the ingredients as follows: 0.2μl Taq DNA polymerase (TakaRa), 2μl 10×Taq buffer, 200nM specific primer pairs, and 2μl of the cDNA template. The resultant PCR amplicons were detected by the electrophoresis on 1.0% agarose gel. Real-time quantitative PCR (qPCR) were also carried out using a 20μl reaction mixture consisted of the following components: i) 10μl 2×SYBR qPCR Mix kit (Aidlab, China); ii) 200nM specific primer pairs (e.g.: MSMEG_2036-F2/R2); and iii) 2μl cDNA product of the RT-PCR. Each reaction was performed in triplicate. Amplification and detection of the products were conducted with QuantStudio applied biosystems (Thermo Fisher Scientific, America), of which the program is an initial denaturation cycle at 95°C for 5 min, followed by 40 cycles of denaturation at 95°C for 15s, annealing at 60°C for 15s, and extension at 72°C for 45s. Data collection on the green channel proceeded at the extension step. High-resolution melting curve of PCR amplicons were plotted with varied temperatures (ranging from 55°C to 99°C with an increase of 0.5°C/10s), which was followed by a final step at 25°C for 5min. The data were analyzed with QuantStudio Design & Analysis Software. Amplification specificity was assessed via melting curve analysis. The sigma A (*sigA*) acted as a reference gene.

### In-frame deletion and knock-in of mycobacterial *bioH* isoforms

To remove the *bioH* isoforms (*bioH1* to *bioH3*) from *M*. *smegmatis* MC^2^ 155, an approach of homologous recombination was utilized. In brief, the upstream and downstream regions (~1kb) flanking certain *bioH* gene, were amplified, and fused with overlapping PCR as earlier described [66, 79]. The resultant fusion PCR products were inserted into the pMind suicide vector [81], giving the knockout plasmids for various *bioH* variants (**S3 Table**). Because that the *sacB*-*lacZ* cassette acted as a selective marker, the transformants of mycobacterial cells electroporated with pMind-derivatives, were screened on 7H10 agar plates having 100μg/ml x-gal and 50μg/ml kanamycin. As for those colonies positive in initial screens for single-crossover events, they were further plated on the selective 7H10 agar plates containing 100μg/ml x-gal and 10% sucrose to screen the double-exchange *bioH* mutant candidates [66, 67]. In addition to the positive control of Δ*bioAFD*, seven mutants of *bioH* isoforms were produced here, including 3 single mutants (Δ*bioH1*, Δ*bioH2*, and Δ*bioH3*), 3 double mutants (Δ*bioH1*/*2*, Δ*bioH2*/*3*, and Δ*bioH1*/*3*), and a triple mutant of Δ*bioH1*/*2*/*3* (**S3 Table**). To generate knock-in strains, the pMV261-*bioH* series (H1 to H3) were complemented into the Δ*bioH1*/*2*/*3* mutant. Namely, they included i) Δ*bioH1*/*2*/*3*+p*bioH1*; ii) Δ*bioH1*/*2*/*3*+p*bioH2*; and iii) Δ*bioH1*/*2*/*3*+p*bioH3* (**S3 Table**). All the resultant mycobacterial knock-out mutants and the knock-in strains were subjected to multiplex PCR analysis combined with direct DNA sequencing.

### Assays for bacterial viabilities

To address function of *bioH* isoforms (*bioH1* to *bioH3*), bacterial viabilities were assayed using two groups of genetically modified strains. In brief, one group denotes the derivatives of *E*. *coli* Δ*bioH* mutant harboring certain *bioH* isoform or its point mutants; the other referred to a collection of *M*. *smegmatis bioH* mutants (from single mutants to triple mutant) plus the complementary strains (**S3 Table**). All the strains of interest were stripped on the non-permissive, biotin-deficient growth conditions (i.e., M9 minimal agar plates for *E*. *coli* derivatives, and 7H10 minimal agar plates for the mutants of *M*. *smegmatis*). In addition, a number of *E*. *coli* strains that carry certain *bioH* mutant defective in either catalytic triad (e.g., S110A, D251A, and H279A for BioH1) or substrate-binding cavity (like R134A and R136A for BioH3), were prepared to plot growth curves. As described with BioJ [40,41], they were sub-cultured (~1:200) into biotin-free M9 liquid minimal media (200μl per well in a given 96-well plate), and maintained at 37°C for overnight. The Spectrophotometer (Spectrum lab S32A) with shaking at 200rpm, was applied to record the OD600 value, of which the regular interval was 1h for *E*. *coli* during the 24h period (and/or 3h for *M*. *smegmatis* within the whole monitoring period of 36h). Three independent experiments were carried out, of which each point was set in triplicate.

### Protein expression, purification, and identification

In total, 11 proteins were involved here. First, the AcpM cargo protein of *Mycobacteria* was cloned and purified, which was almost identical to that of the paradigm holo-ACP (AcpP) of *E*. *coli* [82]. Given that two forms of apo-ACP (inactive) and holo-ACP (active) can co-occur, the purified AcpM and AcpP were judged with the conformationally-sensitive gel of 0.1M urea/17.5% PAGE (pH9.5). Second, to synthesize pimeloyl-ACP methyl ester (M-C7-ACP), a cognate substrate of BioH/BioJ carboxylesterase, the tool enzyme, Acyl-ACP synthetase (AasS) of *V*. *harveyi* was also overexpressed, and purified to homogeneity as recommended by Jiang *et al*. [55]. Third, the observation that somewhat AcpM can be replaced with the prototype AcpP of *E*. *coli* enabled the possibility of the *in vitro* large preparation of pimeloyl-ACP methyl (ethyl) ester with AcpP as the cargo. All the resultant M-C7-ACP (or E-C7-ACP) protein was determined by the separation with the conformationally-sensitive gel of 0.5M urea/PAGE (pH9.5, 17.5%).

The prokaryotic expression system of BL21(DE3) carrying a pET28a-borne *bioH* (*H1* to *H3*) was developed, in aiming to prepare the three BioH3 isoforms we identified here (**S2** and **S3 Tables**). In general, the temperature was lowered to 18°C, once bacterial OD_600_ reached 0.6~0.8. Then, the cultures were induced with 0.2mM IPTG (isopropyl β-D-1-thiogalactopyranoside for 20h. Bacterial cells were collected by centrifugation, resuspended in the buffer A [20mM Tris-HCl (pH 8.0), 150mM NaCl, 25mM Imidazole), and lysed by ultrahigh-pressure homogenizer. Cellular debris was removed by centrifugation and the supernatant was routinely loaded onto the nickel column (GE Healthcare). Following the removal of the contaminated proteins, the target protein was eluted with the elution buffer B [20mM Tris-HCl (pH 8.0), 150mM NaCl, 150mM imidazole]. As a result, the BioH1 (MSMEG_2036) seemed weird in that it consistently forms inclusion body despite that we tried different expression strategies, even in the trials of *in situ M*. *smegmatis*. It was noted that the no active form can be recovered from the urea-dissolving BioH3 solution after the routine dialysis-aided refolding, which largely hampered the subsequent biochemical analysis. By contrast, the hexa-histidine-tagged protein of both BioH2 and BioH3 gave soluble forms. The resultant BioH2 and BioH3 proteins were dialyzed with the buffer C [20 mM Tris-HCl (pH 8.0), 150 mM NaCl], and concentrated to ~15mg/ml, prior to the analysis of size exclusion chromatography with a Superdex 200 Increase column (GE Healthcare). Unlike BioH2 forming oligomer/solute aggregate, BioH3 was eluted at the dimeric position in the gel filtration. Almost identical to that of wild-type BioH3, its three additional single mutants that are defective in catalytic triad (S103A, D232A, and H265A), were also overexpressed, and purified to homogeneity. As earlier described by Feng *et al*. [83] with appropriate adjustments, chemical cross-linking assay with the crosslinker of EGS [ethylene glycol bis (succinimidyl succinate)] were performed to evaluate the solution structure of BioH3.

Additionally, Seleno-methionine (Se-Met) substituted BioH3 protein was prepared for subsequent structural study. In brief, bacterial cells were cultured in 1 liter of M9 medium containing 30μg/ml L-selenomethionine, and induced overnight with 0.2mM IPTG at 18°C. The collected cells resuspended in the buffer C [20mM Tris-HCl (pH 8.0), 500mM NaCl, 25mM Imidazole] were lysed with ultrahigh-pressure homogenizer. The cellular debris-free supernatant was loaded onto a HisTrap HP column (GE Healthcare). The target protein was eluted with the buffer D [20mM Tris-HCl (pH 8.0), 500mM NaCl, 500mM imidazole], and further purified by Hiload 16/60 Superdex G75 column (GE Healthcare). Peak fractions containing the target proteins were pooled and concentrated. The purity of SeMet-BioH3 protein was analyzed using SDS-PAGE gel, the concentration was measured using a UV-spectrophotometer at 280nm.

### Enzymatic analysis of BioH2 and BioH3

Like BioH [23,31] and its distinct isoform BioJ [40,41], the two biochemically-amenable BioH isoforms (BioH2 and BioH3) we identified from Mycobacteria are presumably capable of demethylating the substrate of pimeloyl-ACP methyl ester. As performed with BioJ [40], the enzymatic reactions of BioH2 and BioH3 were established *in vitro*. Apart from 50mM HEPES buffer (pH7.0) containing 5% glycerol, the reaction system (20μl in total) included 150μM substrate of M-C7-ACP (or E-C7-ACP) mixed with 5nM BioH2 (or BioH3). Following the 0.5h of incubation at 37°C, the mixture of reaction (10μl) was separated with the conformationally-sensitive gel of 0.5M urea/17.5% PAGE (pH9.5, 130V/2.5h). The product of C7-ACP was supposed to be distinguishable from its reactant of M-C7-ACP (or E-C7-ACP), because of its slower migration rate than that of the substrate in this unique gel.

### MALDI-TOF and LC mass spectrometry

To confirm the identity of the product C7-ACP visualized in the aforementioned conformationally-sensitive gel, the BioH2 (and/or BioH3) reaction mixture was subjected to the analysis of Matrix-Assisted Laser Desorption Ionization Time of Flight (MALDI-TOF) [40]. Prior to MALDI-TOF, the samples were treated as follows: i) to precipitate with cold isopropyl alcohol; ii) to resuspend with 0.2 ml ammonium acetate (20mM); and iii) to dialyze in 200ml solution of 20mM ammonium acetate at 4°C. Then, the molecular mass of the freeze-dried sample was measured with the MALDI-TOF technique (Bruker, ultraflextreme). The theoretical masses of M-C7-ACP, and its product C7-ACP, are 9003.520 m/z, and 8989.3 m/z, respectively. Of note, BioJ, the BioH isoenzyme with known activity [40], was assayed here as the positive control.

As described with BioZ [37] with little change, Liquid Chromatography (LC) mass spectrometry was adopted to detect the specific residue of ACP cargo with pimeloyl moiety. The interested C7-ACP protein band cut from the urea/PAGE gel, was digested with pepsin (but not trypsin), giving the mixture of peptides. Prior to the entry into an analytical column (50μm×15cm, nanoviper, C18, 2μM,100Å), those peptides were loaded into the trap column (Thermo Scientific Easy nanoLC 1000). Data collection was mainly dependent on Thermo LTQ-Orbitrap Elite Ion Trap analyzer (Thermo Scientific, USA) as well as FTMS (Fourier transform ion cyclotron resonance mass). The reliable MS spectrums of candidate peptides were determined using the software of Proteome Discoverer 2.0. It was noted that the value of 484.1644 as the theoretical mass, is assigned to the phosphopantetheine (Ppan) moiety with pimeloyl modification.

### Bioassay for biotin biosynthesis

To visualize a role of certain BioH isoenzyme in biotin synthesis, the *in vitro* reconstituted system for *de novo* biotin biosynthesis was established as earlier reported by Lin and coauthors [23] with little change. The preparation of the cell-free crude extract of STL24, the *E*. *coli* Δ*bioH* mutant, could provide series of FAS II enzymes along with biotin synthesis enzymes except with BioH. To eliminate the contamination of biotin/DTB, bacterial crude extract was dialyzed with 1xPBS buffer. In addition to 1mg crude extract supplemented with 0.5μM BioH2 (or BioH3) enzyme, the *in vitro* reaction system (100μl in total) also contained 10 different components as follows: i) 10mM MgCl_2_; ii) 5mM dithiothreitol (DTT); iii) 0.1mM pyridoxal-5′-phosphate (PLP); iv) 1mM L-alanine; v) 1mM KHPO_4_; vi) 1mM NADPH; vii) 1mM ATP; viii) 1mM glucose 6-phosphate (G6P); ix) 1mM S-adenosy-L-methionine (SAM); and x) 60μM M-C7-ACP (or E-C7-ACP). The mixture of reaction was kept at 37°C for ~3h, quenched by the immersion in boiling water for 15min, and then spined at 13600rpm for 20min. The resultant pellet was discarded, and the supernatant was supposed to contain the biotin product.

Two alternative indicator bacteria used here, separately corresponded to i) Strain ER90 (Δ*bioFCD*) of *E*. *coli* [23] and ii) Strain FYJ283 (Δ*bioBFDA*) of *A*. *tumefaciens* [37,47]. Clearly, both of them were biotin auxotrophic. In general, the earlier log-phase cultures of the indicator strains were prepared, washed twice, and mixed appropriately into the melted M9 minimal agar medium (55~60°C), giving an array of quadruple-sectored M9 indicating agar plates. It was noted that 0.01% of 2,3,5-triphenyl tetrazolium chloride (TTC) added into the aforementioned M9 indictor plates is reduced to give insoluble red formazan upon the biotin-initiating growth of biotin auxotroph. In principle, an indicator strain cannot give any growth unless the supply of biotin arising from the *in vitro* enzyme system.

### Crystallization, data collection and structural determination

The Se-substituted form of BioH3 protein was concentrated to 18 mg/ml for crystallization screens. The initial crystallization conditions were identified at 16°C using the Gryphon crystallization robot system and commercial crystallization kits. The sitting-drop vapor diffusion method was utilized during both initial screening and optimization process. The crystallization condition is composed of 20mM Citric acid, 80mM Bis-Tris propane pH8.8 and 16% (w/v) PEG3350, the drop contains 0.2μl BioH3 protein sample and 0.2μl crystallization solution. The crystals appeared next day, and reached their full sizes within one week. All crystals were cryoprotected in reservoir solution supplemented with 25% (v/v) glycerol and snap-frozen in liquid nitrogen. The x-ray diffraction data were collected on beamlines BL17U and BL19U at the Shanghai Synchrotron Radiation Facility (SSRF). Data were automatically processed by the autoPROC_XDS program developed by the beamline staff. The data collection and processing statistics were listed in **Table 1**.

The BioH3 structure was solved by the single-wavelength anomalous diffraction (SAD) method with the Autosol program embedded in the Phenix suit [84]. The initial model was built using the Autobuilt program and then refined against the diffraction data using the Refmac5 program of the CCP4 suite [85]. The 2F_o_–F_c_ and F_o_–F_c_ electron density maps were regularly calculated and used as guide for the building of the missing amino acids using COOT [86]. Water and other molecules were all built manually using COOT. The structure was refined using the phenix.refine program of Phenix suit. The structural refinement statistics were available in **Table 1**.

### Bioinformatics

The three BioH isoforms (BioH1 to BioH3) we identified in this study, were aligned with the paradigm BioH of *E*. *coli*. The similarity of BioH1 (MSMEG_2036) to its counterparts in the TB-causing bacterium (GS11_3319 and Rv3171c) was evaluated. Also, the sequences of AcpM from three mycobacterial species (*M*. *tuberculosis*, *M*. *bovis*, and *M*. *smegmatis*) were compared with AcpP of *E*. *coli* MG1655. Multiple sequence alignments were conducted using ClustalOmega (https://www.ebi.ac.uk/Tools/msa/clustalo), and the resultant BLAST outputs were given after the process by the ESPript 3.0 program (https://espript.ibcp.fr/ESPript/cgi-bin/ESPript.cgi). The structures of BioH1 inclusion body and BioH2 oligomer were predicted with Alpha-Fold2 (https://alphafold.ebi.ac.uk) [57,58]. Ribbon presentations for all the protein structures were generated using PyMol (https://pymol.org/2), namely BioH3, BioH, BioG, BioJ, AcpP, and AcpM. Phylogeny of BioH isoforms (BioH1 to BioH3) was generated using the MEGA7 software (https://www.megasoftware.net). On the basis of Jones-Taylor-Thornton (JTT) model, a maximum-likelihood tree of BioH was given, in which the number of bootstrap replications is 1000.

## Supporting information

S1 Table BioC orthologs in *M*. *smegmatis* MC^2^ 155. (XLSX)

S2 Table A collection of putative α/β-hydrolases from *M*. *smegmatis* MC^2^ 155. The symbol “/” denotes the absence of homologs.
(XLSX)

S3 Table Bacteria and plasmids used in this study. (DOCX)

S4 Table A list of DNA oligos used in this study. (DOCX)

S1 Fig A scheme of expression cloning strategy used for discovery of BioH-like activity from *M*. *smegmatis* MC^2^ 155. (TIF)

S2 Fig Sequence alignment of the three BioH isoenzymes of *M*. *smegmatis* with the paradigmatic BioH, the *E*. *coli b3412* gene product. Inside table displays identity across different BioH members. The residues of catalytic triad are labeled with arrows. Namely, they included i) S82, D207 & H235 (EcBioH); ii) S110, D251 & H279 (BioH1); iii) S127, D255 & H283 (BioH2); and iv) S103, D232 & H265 (BioH3). Of note, crystal structure of BioH3 argued that the conserved D248 participates into catalytic triad. In contrast, it is replaced with D232. Clustal Omega (https://www.ebi.ac.uk/Tools/msa/clustalo/) was used to conduct sequence alignment. Identical residues are indicated with white letters in red background, similar sites are shown with dark letters in yellow background, different residues are indicated with black letters, and gaps are denoted with dots.
(TIF)

S3 Fig Functional identification of GS11_3319 of *M*. *bovis* BCG, equivalent to BioH1 (MSMEG_2036) of *M*. *smegmatis*. **A.** Genomic environment of *bioH* and other *bio*-related loci in *M*. *bovis* BCG strain 3281. **B.** Sequence alignment of GS11_3319 of *M*. *bovis* BCG with the counterpart of *M*. *smegmatis*. Compared with MSMEG_2036, the identity of GS11_3319 (and/or Rv3171c) is estimated to 57–58%. The three residues forming catalytic triad of GS11_3319 are labeled, namely S122, D263, and H291. **C.** The presence of MSMEG_2036 of *M*. *smegmatis* renders the Δ*bioH* biotin auxotrophic strain to appear on the non-permissive growth condition defective in biotin. **D.** Functional expression of GS11_3319 of *M*. *bovis* BCG allows growth of the Δ*bioH* biotin auxotrophic strain on the biotin-lacking condition. Designations: α, α-helices; β, β-sheet; T, T-turn; η, coils; vec, pBAD24 vector; ara, arabinose; bpl, biotin protein ligase.
(TIF)

S4 Fig Use of size exclusion chromatography to compare solution structures of BioH2 (MSMEG_1352) and BioH3 (MSMEG_6710). Unlike BioH3 (MSMEG_6710) forming a dimer, gel filtration analysis revealed that BioH2(MSMEG_1352) behaves as a soluble aggregate. The inside gels denote SDS-PAGE profiles of both BioH2 and BioH3. Gel filtration assays were performed with a Superdex 200 Increase column (of note, this is a 6-year-used column of which protein elution volume is little bit lagged when compared with the brand new one).
(TIF)

S5 Fig Preparation and identification of M-C7-ACP. **A.** Representative diagram for AasS-based synthesis of M(E)-C7-ACP. Designations: M-C7-ACP, Methyl pimeloyl-ACP; E-C7-ACP, Ethyl pimeloyl-ACP. **B.** AasS catalyzes the synthesis of M-C7-ACP *in vitro*. **C.** MS identification for an ACP-arising peptide with M-C7 modification.
(TIF)

S6 Fig Functional equivalence of mycobacterial AcpM to the paradigm *E*. *coli* AcpP. Genomic context of *E*.*coli* AcpP (**A**) and *M*. *smegmatis* AcpM (**B**). **C.** Sequence alignment of the three AcpM homologs of *Mycobacteria* with the paradigmatic AcpP of *E*. *coli* MG1655. **D.** Ribbon structure of the *E*. *coli* AcpP (PDB: 4IHH). **E.** Overall structure of mycobacterial AcpM (PDB: 7BVH). S36 (S40) colored hot pink, denotes the residue of AcpP attached with a methyl-pimeloyl moiety. Ribbon graphs were plotted with the software of PyMol (https://pymol.org/2). **F.** The attachment of M-C7 by AasS suggests functional equivalence between MsAcpM and EcAcpP. Similar to that of EcAcpP, MsAcpM is also ligated with M-C7 by AasS, giving M-C7-ACP. Conformationally-sensitive gel [0.5M urea/17.5% PAGE (pH9.5)] was applied to separate the M-C7-ACP product from its reactant holo-ACP. **G.** M-C7-AcpM is a functional substrate for BioH2(MSMEG_1352). Here, 2 pmol of *Pseudomonas* PA0502 protein, a known BioH esterase [87], was added as the positive control. The minus denotes no addition of PA0502 or BioH2(MSMEG_1352). The top triangle on right hand represents the enzyme BioH2 at various level ranging from 5, 10, 20, 60, to 80 pmol. Designations: AcpP, acyl carrier protein; AcpM, ACP of *Mycobacteria*; PlsX, Phosphate: acyl-ACP acyltransferase; FabH, β-ketoacyl-ACP synthase III; FabD, Malonyl-CoA: acyl trans-acylase; FabG, 3-oxoacyl-ACP reductase; FabF, 3-oxoacyl-ACP synthase; KasA1 & KasA2: two isoforms of FAS-II β-ketoacyl-ACP synthase with 65.06% identity; Ec, *E*. *coli*; Mt, *Mycobacterium tuberculosis*; Mb, *Mycobacterium tuberculosis* variant bovis BCG; Ms, *Mycobacterium smegmatis* MC2 155; N, N-terminus; C, C-terminus; α, α-helices; S36(40), Serine at the position of 36 (40); AasS, Acyl-ACP synthetase; M-C7, methyl-pimelic acid; M-C7-ACP, pimeloyl-ACP methyl ester.
(TIF)

S7 Fig The three BioH3 mutants of which catalytic triad is disrupted remain of dimeric structure. **A.** Size exclusion chromatography of BioH3 and its mutants with certain defection in the catalytic triad. **B.** SDS-PAGE profile for BioH3 and its three derivatives mutated in the catalytic triad. Namely, the three mutants included BioH3(S103A), BioH3(D248A), and BioH3(H265A). Designations: WT, Wild-type of BioH3; M, Protein marker; kDa, kilo-dalton.
(TIF)

S8 Fig Functional unification of catalytic triads amongst three BioH isoenzymes. **A.** Use of structure-guided, site-directed mutagenesis to assay the function of the catalytic triad (S110, D251 & H279) of BioH1 (MSMEG_2036). **B.** Functional analysis for the catalytic triad (S127, D255 & H283) of BioH2 (MSMEG_1352). **C.** Functional dissection of the catalytic triad (S103, D232 & H265) from BioH3 (MSMEG_6710). To address effect on *bioH* by numbers of single mutations in catalytic triad, bacterial viabilities of the recipient hosts (arising from the Δ*bioH* biotin auxotrophic strain) were tested on the non-permissive condition lacking biotin. A representative result was given.
(TIF)

S9 Fig Alanine substitution of catalytic triad impairs ability of GS11_3319 to confer viability of the Δ*bioH* biotin auxotrophic strain on the biotin-deficient, non-permissive growth condition. The catalytic triad of GS11_3319 is constituted by the following three residues, namely S122, D263, and H291.
(TIF)

S10 Fig The BioH3(D248A) mutant is not capable of augmenting bacterial growth of the Δ*bioH* biotin auxotrophic strain on the non-permissive, biotin-deficient condition. A representative result was shown.
(TIF)

S11 Fig Proposal for a PEG-binding cavity of BioH3. **A.** Structural superposition of the core domains from BioH3 and its three isoforms (BioH, BioG, and BioJ). They were separately colored blue for BioH3, green for BioH, yellow for BioG, and wheat for BioJ. **B.** Enlarged view of PEG-binding cavity of BioH3. Seven residues are proposed to surround PEG molecule. Namely, they are W18, F137, I141, L152, I154, F183, and L208. The 2F_o_-F_c_ electron density maps of PEG were contoured at 1.2δ level. **C.** Conformational comparison of the PEG-mimicking substrate cavity of BioH3 with that of BioH. It elucidated that four of the PEG-binding residues are conserved, but vary dramatically in their orientations. Namely, they denoted i) W18, F137, F183 & L235 for BioH3; and ii) W22, F111, F143 & L209 for BioH. Residues of BioH3 and BioH were colored in blue and green, respectively. PEG was shown with spheres in atomic color.
(TIF)

S12 Fig Structural analysis of binding of *Shigella* BioH to its cognate substrate M-C7-ACP. **A.** Surface structure of *Shigella* BioH demethylase. The M-C7-loading tunnel is highlighted with a dashed line rectangle. M-C7 is denoted with sticks and indicated with an arrow. Red denotes negative charge, and blue refers to positive charge. **B.** Ribbon structure of the complex of *Shigella* BioH and its substrate M-C7-ACP. The complex structure was generated using BioH/M-C7-ACP (PDB: 4ETW). As for ribbon structure, BioH is colored cyan, and M-C7-ACP is shown in orange. The ionic interaction between BioH and the α2-helices M-C7-ACP is underlined with a dashed line rectangle. M-C7 is denoted with purple sticks. **C.** Enlarged views of ionic interactions between BioH and the α2-helices of M-C7-ACP. Presumably, the first site of the ionic interaction engages two basic residues (R138 and R142) of BioH, pairing with three acidic amino acids (Q14, D35, and D38) from ACP α2-helices; the second site of salt bridge involves two positively-charged residues (R155 and R159) of BioH and three negatively-charged residues (E47, I54 & D56) of ACP α2-helices. Designations: α, α-helices; N, N-terminus; C, C-terminus; ACP, Acyl carrier protein; M-C7, methyl pimeloyl chain; M-C7-ACP, methyl pimeloyl-ACP ester.
(TIF)

S13 Fig Substrate cavity of BioH3 inferred from its comparison with the structure BioH/M-pim-ACP complex. **A.** Overall folding of BioH complexed with its M-pim-ACP substrate. The core and lid domains of BioH were separately shown as white surface and magenta cartoon. M-pimeloyl moiety was indicated with spheres in atomic color, and its ACP cargo was shown in green cartoon. **B.** Crystal structure of BioH3 enzyme. The lid domain was given as cartoon in blue, whereas the gate region was shown as sticks outlined with 2F_o_-F_c_ electron density maps (contour level, 1.2δ). Five residues are included, namely P144, V150, P153, F155, and P158. **C.** The M-C7 cavity gate of BioH3 inferred by its structural superposition with BioH. The core domains were displayed as white surface. The lid of BioH3 was given with blue cartoon, and the counterpart of BioH was shown as magenta cartoon. The M-C7 moiety indicated with an arrow, was denoted with a stick. The two α-helices (α4 and α5) of BioH that participate in its M-C7 moiety-loading cavity, are replaced by the α4-α5 linker in BioH3. **D.** Distribution of Arginine residues near the M-C7 cavity gate in the BioH3 structure. The PEG molecule mimicking the M-C7 fatty acyl chain, was indicated with spheres in atomic color. The substrate cavity gate is highlighted with dashed line. Five positively-charged basic residues around the cavity gate were presented with spheres in atomic color. Namely, they included R134, R136, R217, R218, and R249.
(TIF)

S14 Fig The removal of triple *bioH* (H1 to H3) renders *M*. *smegmatis* biotin auxotrophic. **A.** Bacterial viability on 7H10 minimal agar plates revealed that the triple *bioH1*/*2*/*3* mutant (Δ*bioH1*/*2*/*3*) of *M*. *smegmatis* MC^2^ 155 is biotin auxotroph, and can be rescued upon the addition of up to 16nM exogenous biotin. **B.** Use of growth curves enabled us to conclude that unlike the single/double mutant of *bioH* (H1 to H3) retaining robust viability, the triple mutant Δ*bioH1*/*2*/*3* exhibits serious defection in bacterial growth. In addition to WT, eight mutants were tested here, namely i) Δ*bioAFD*, ii) three single mutants (Δ*bioH1*, Δ*bioH2*, and Δ*bioH3*) iii) three double mutants (Δ*bioH1*/*2*, Δ*bioH1*/*3*, and Δ*bioH2*/3), and iv) a triple mutant (Δ*bioH1*/*2*/*3*). The positive control referred to the Δ*bioAFD* mutant, a biotin auxotrophic strain. Designations: WT, *M*. *smegmatis* MC^2^ 155.
(TIF)
