# Supplementary material for: Three enigmatic BioH isoenzymes are programmed in the early stage of mycobacterial biotin synthesis, an attractive anti-TB drug target
Source: PLoS Pathog. 2022 Jul 11;18(7):e1010615. doi: 10.1371/journal.ppat.1010615 (PMC9302846; doi:10.1371/journal.ppat.1010615)
Supplement: S3 Table — (DOCX) [file ppat.1010615.s003.docx]

**Table S3** Bacteria and plasmids used in this study

| Strains or plasmids | Relevant characteristics | Origins |
| --- | --- | --- |
| Strains | | |
| DH5α | A cloning host of *E. coli* | Lab stock |
| MG1655 | A wild-type strain of *E. coli* | Lab stock |
| BL21(DE3) | An expression host of *E. coli* | Lab stock |
| FYJ283 | A biotin auxotroph of *Agrobacterium*  *tumefaciens* NTL4 (Δ*bioBFDA*) | Lab stock ^1^ |
| ER90 | A biotin auxotrophic strain of MG1655  (Δ*bioFCD*) | Lab stock ^2^ |
| STL24 | MG1655, Δ*bioH* | Lab stock ^2^ |
| STL96 | MG1655, Δ*bioC* | Lab stock ^3^ |
| FYJ391 | ST24(MG1655, Δ*bioH*) carrying pBAD322 | Lab stock |
| FYJ415 | STL96(MG1655, Δ*bioC*) carrying pBAD322 | Lab stock |
| FYJ557 | DH5α harboring pMind | Lab stock ^4^ |
| FYJ558 | DH5α carrying pMV261 | Lab stock |
| FYJ559 | DH5α carrying pGoAL17 | Lab stock |
| FYJ563 | *Mycobacterium smegmatis* MC^2^ 155 | Lab stock |
| FYJ5200 | ST24 carrying pBAD322*::*MSMEG_2036 | This work |
| FYJ5201 | ST24 carrying pBAD322*::*MSMEG_1352 | This work |
| FYJ5202 | ST24 carrying pBAD322*::*MSMEG_6710 | This work |
| FYJ5203 | ST24 carrying pBAD322*::*MSMEG_0117 | This work |
| FYJ5204 | ST24 carrying pBAD322*::*MSMEG_0267 | This work |
| FYJ5205 | ST24 carrying pBAD322*::*MSMEG_0280 | This work |
| FYJ5206 | ST24 carrying pBAD322*::*MSMEG_0289 | This work |
| FYJ5207 | ST24 carrying pBAD322*::*MSMEG_0302 | This work |
| FYJ5208 | ST24 carrying pBAD322*::*MSMEG_0460 | This work |
| FYJ5209 | ST24 carrying pBAD322*::*MSMEG_0605 | This work |
| FYJ5210 | ST24 carrying pBAD322*::*MSMEG_1108 | This work |
| FYJ5212 | ST24 carrying pBAD322*::*MSMEG_1570 | This work |
| FYJ5213 | ST24 carrying pBAD322*::*MSMEG_1576 | This work |
| FYJ5214 | ST24 carrying pBAD322*::*MSMEG_1587 | This work |
| FYJ5215 | ST24 carrying pBAD322*::*MSMEG_1655 | This work |
| FYJ5216 | ST24 carrying pBAD322*::*MSMEG_1797 | This work |
| FYJ5217 | ST24 carrying pBAD322*::*MSMEG_1940 | This work |
| FYJ5218 | ST24 carrying pBAD322*::*MSMEG_1984 | This work |
| FYJ5219 | ST24 carrying pBAD322*::*MSMEG_1998 | This work |
| FYJ5220 | ST24 carrying pBAD322*::*MSMEG_2074 | This work |
| FYJ5221 | ST24 carrying pBAD322*::*MSMEG_2203 | This work |
| FYJ5222 | ST24 carrying pBAD322*::*MSMEG_2409 | This work |
| FYJ5223 | ST24 carrying pBAD322*::*MSMEG_2517 | This work |
| FYJ5224 | ST24 carrying pBAD322*::*MSMEG_2534 | This work |
| FYJ5225 | ST24 carrying pBAD322*::*MSMEG_2568 | This work |
| FYJ5226 | ST24 carrying pBAD322*::*MSMEG_2612 | This work |
| FYJ5227 | ST24 carrying pBAD322*::*MSMEG_2745 | This work |
| FYJ5228 | ST24 carrying pBAD322*::*MSMEG_2767 | This work |
| FYJ5229 | ST24 carrying pBAD322*::*MSMEG_2777 | This work |
| FYJ5230 | ST24 carrying pBAD322*::*MSMEG_2875 | This work |
| FYJ5231 | ST24 carrying pBAD322*::*MSMEG_2888 | This work |
| FYJ5232 | ST24 carrying pBAD322*::*MSMEG_2900 | This work |
| FYJ5233 | ST24 carrying pBAD322*::*MSMEG_2984 | This work |
| FYJ5234 | ST24 carrying pBAD322*::*MSMEG_3010 | This work |
| FYJ5235 | ST24 carrying pBAD322*::*MSMEG_3059 | This work |
| FYJ5236 | ST24 carrying pBAD322*::*MSMEG_3287 | This work |
| FYJ5237 | ST24 carrying pBAD322*::*MSMEG_3289 | This work |
| FYJ5238 | ST24 carrying pBAD322*::*MSMEG_3297 | This work |
| FYJ5239 | ST24 carrying pBAD322*::*MSMEG_3336 | This work |
| FYJ5240 | ST24 carrying pBAD322*::*MSMEG_3508 | This work |
| FYJ5241 | ST24 carrying pBAD322*::*MSMEG_3829 | This work |
| FYJ5242 | ST24 carrying pBAD322*::*MSMEG_3842 | This work |
| FYJ5243 | ST24 carrying pBAD322*::*MSMEG_3856 | This work |
| FYJ5244 | ST24 carrying pBAD322*::*MSMEG_3891 | This work |
| FYJ5245 | ST24 carrying pBAD322*::*MSMEG_3979 | This work |
| FYJ5246 | ST24 carrying pBAD322*::*MSMEG_4148 | This work |
| FYJ5247 | ST24 carrying pBAD322*::*MSMEG_4202 | This work |
| FYJ5248 | ST24 carrying pBAD322*::*MSMEG_4295 | This work |
| FYJ5249 | ST24 carrying pBAD322*::*MSMEG_4450 | This work |
| FYJ5250 | ST24 carrying pBAD322*::*MSMEG_4707 | This work |
| FYJ5251 | ST24 carrying pBAD322*::*MSMEG_4810 | This work |
| FYJ5252 | ST24 carrying pBAD322*::*MSMEG_4815 | This work |
| FYJ5253 | ST24 carrying pBAD322*::*MSMEG_4860 | This work |
| FYJ5254 | ST24 carrying pBAD322*::*MSMEG_4867 | This work |
| FYJ5255 | ST24 carrying pBAD322*::*MSMEG_4998 | This work |
| FYJ5256 | ST24 carrying pBAD322*::*MSMEG_5107 | This work |
| FYJ5257 | ST24 carrying pBAD322*::*MSMEG_5171 | This work |
| FYJ5258 | ST24 carrying pBAD322*::*MSMEG_5209 | This work |
| FYJ5259 | ST24 carrying pBAD322*::*MSMEG_5210 | This work |
| FYJ5260 | ST24 carrying pBAD322*::*MSMEG_5220 | This work |
| FYJ5261 | ST24 carrying pBAD322*::*MSMEG_5271 | This work |
| FYJ5262 | ST24 carrying pBAD322*::*MSMEG_5278 | This work |
| FYJ5263 | ST24 carrying pBAD322*::*MSMEG_5341 | This work |
| FYJ5264 | ST24 carrying pBAD322*::*MSMEG_5350 | This work |
| FYJ5265 | ST24 carrying pBAD322*::*MSMEG_5652 | This work |
| FYJ5266 | ST24 carrying pBAD322*::*MSMEG_5763 | This work |
| FYJ5267 | ST24 carrying pBAD322*::*MSMEG_5823 | This work |
| FYJ5268 | ST24 carrying pBAD322*::*MSMEG_5878 | This work |
| FYJ5269 | ST24 carrying pBAD322*::*MSMEG_6029 | This work |
| FYJ5270 | ST24 carrying pBAD322*::*MSMEG_6037 | This work |
| FYJ5271 | ST24 carrying pBAD322*::*MSMEG_6085 | This work |
| FYJ5272 | ST24 carrying pBAD322*::*MSMEG_6106 | This work |
| FYJ5273 | ST24 carrying pBAD322*::*MSMEG_6151 | This work |
| FYJ5274 | ST24 carrying pBAD322*::*MSMEG_6184 | This work |
| FYJ5275 | ST24 carrying pBAD322*::*MSMEG_6381 | This work |
| FYJ5276 | ST24 carrying pBAD322*::*MSMEG_6533 | This work |
| FYJ5277 | ST24 carrying pBAD322*::*MSMEG_6586 | This work |
| FYJ5278 | ST24 carrying pBAD322*::*MSMEG_6597 | This work |
| FYJ5279 | ST24 carrying pBAD322*::*MSMEG_6658 | This work |
| FYJ5280 | ST24 carrying pBAD322*::*MSMEG_6708 | This work |
| FYJ5281 | ST24 carrying pBAD322*::*MSMEG_6719 | This work |
| FYJ5282 | ST24 carrying pBAD322*::*MSMEG_6720 | This work |
| FYJ5283 | ST24 carrying pBAD322*::*MSMEG_6763 | This work |
| FYJ5284 | ST24 carrying pBAD322*::*MSMEG_6772 | This work |
| FYJ5285 | ST24 carrying pBAD322*::*MSMEG_6831 | This work |
| FYJ5286 | ST24 carrying pBAD322*::*MSMEG_6838 | This work |
| FYJ5287 | ST24 carrying pBAD322*::*MSMEG_6850 | This work |
| FYJ5288 | ST24 carrying pBAD322*::*MSMEG_6852 | This work |
| FYJ5289 | ST24 carrying pBAD322*::*MSMEG_6906 | This work |
| FYJ5290 | ST24 carrying pBAD322*::*BCG_3195c | This work |
| FYJ5291 | ST24 carrying pBAD322*::*BCG_3195c (S110A) | This work |
| FYJ5292 | ST24 carrying pBAD322*::*BCG_3195c (D263A) | This work |
| FYJ5293 | ST24 carrying pBAD322*::*BCG_3195c (H291A) | This work |
| FYJ5294 | ST24 carrying pBAD322*::*BCG_0695c | This work |
| FYJ5295 | ST24 carrying pBAD322*::*BCG_2728 | This work |
| FYJ5296 | ST24 carrying pBAD322*::*MSMEG_2036 (S110A) | This work |
| FYJ5297 | ST24 carrying pBAD322*::*MSMEG_2036 (D251A) | This work |
| FYJ5298 | ST24 carrying pBAD322*::*MSMEG_2036 (H279A) | This work |
| FYJ5299 | ST24 carrying pBAD322*::*MSMEG_1352 (S127A) | This work |
| FYJ5300 | ST24 carrying pBAD322*::*MSMEG_1352 (D255A) | This work |
| FYJ5301 | ST24 carrying pBAD322*::*MSMEG_1352 (H283A) | This work |
| FYJ5302 | ST24 carrying pBAD322*::*MSMEG_6710 (S103A) | This work |
| FYJ5303 | ST24 carrying pBAD322*::*MSMEG_6710 (D232A) | This work |
| FYJ5304 | ST24 carrying pBAD322*::*MSMEG_6710 (H265A) | This work |
| FYJ5305 | ST24 carrying pBAD322*::*MSMEG_6710 (R134A) | This work |
| FYJ5306 | ST24 carrying pBAD322*::*MSMEG_6710 (R136A) | This work |
| FYJ5307 | ST24 carrying pBAD322*::*MSMEG_6710 (R180A) | This work |
| FYJ5308 | ST24 carrying pBAD322*::*MSMEG_6710 (R181A) | This work |
| FYJ5309 | ST24 carrying pBAD322*::*MSMEG_6710 (R196A) | This work |
| FYJ5310 | ST24 carrying pBAD322*::*MSMEG_6710 (R217A) | This work |
| FYJ5311 | ST24 carrying pBAD322*::*MSMEG_6710 (R218A) | This work |
| FYJ5312 | ST24 carrying pBAD322*::*MSMEG_6710 (R221A) | This work |
| FYJ5313 | ST24 carrying pBAD322*::*MSMEG_6710 (R233A) | This work |
| FYJ5314 | ST24 carrying pBAD322*::*MSMEG_6710 (R249A) | This work |
| FYJ5315 | ST24 carrying pBAD322*::*MSMEG_6710 (R217A/R218A) | This work |
| FYJ5316 | ST24 carrying pBAD322*::*MSMEG_6710 (R217A/R218A/R221A) | This work |
| FYJ5317 | ST24 carrying pBAD322*::*MSMEG_6710 (R217A/R218A/R221A/R249A) | This work |
| FYJ5318 | BL21 carrying pET28a*::*MSMEG_2036 | This work |
| FYJ5319 | BL21 carrying pET28a*::*MSMEG_1352 | This work |
| FYJ5320 | BL21 carrying pET28a*::*MSMEG_6710 | This work |
| FYJ5321 | BL21 carrying pET28a*::*MSMEG_2036 (S110A) | This work |
| FYJ5322 | BL21 carrying pET28a*::*MSMEG_2036 (D251A) | This work |
| FYJ5323 | BL21 carrying pET28a*::*MSMEG_2036 (H279A) | This work |
| FYJ5324 | BL21 carrying pET28a*::*MSMEG_1352 (S127A) | This work |
| FYJ5325 | BL21 carrying pET28a*::*MSMEG_1352 (D255A) | This work |
| FYJ5326 | BL21 carrying pET28a*::*MSMEG_1352 (H283A) | This work |
| FYJ5327 | BL21 carrying pET28a*::*MSMEG_6710 (S103A) | This work |
| FYJ5328 | BL21 carrying pET28a*::*MSMEG_6710 (D232A) | This work |
| FYJ5329 | BL21 carrying pET28a*::*MSMEG_6710 (H265A) | This work |
| FYJ5330 | BL21 carrying pET28a*::*BCG_3195c | This work |
| FYJ5331 | BL21 carrying pET28a*::*BCG_0695c | This work |
| FYJ5332 | BL21 carrying pET28a*::*BCG_2728 | This work |
| FYJ5333 | A biotin auxotroph of *M. smegmatis* MC^2^ 155 of which *the bioAFD* operon is in frame deleted (Δ*bioAFD*) | This work |
| FYJ5334 | The *bioH1* (MSMEG_2036) deletion mutant of *M. smegmatis* (ΔMSMEG_2036, Δ*bioH1*) | This work |
| FYJ5335 | The *bioH2* (MSMEG_1352) deletion mutant of *M. smegmatis* (ΔMSMEG_1352, Δ*bioH2*) | This work |
| FYJ5336 | The *bioH3* (MSMEG_6710) deletion mutant of *M. smegmatis* (ΔMSMEG_6710, Δ*bioH3*) | This work |
| FYJ5337 | The double mutant of *M. smegmatis* (Δ*bioH1* /2, ΔMSMEG_2036/ΔMSMEG_1352) | This work |
| FYJ5338 | The double mutant of *M. smegmatis* (Δ*bioH2* /3, ΔMSMEG_1352/ΔMSMEG_6710) | This work |
| FYJ5339 | The double mutant of *M. smegmatis* (Δ*bioH1* /3, ΔMSMEG_2036/ΔMSMEG_6710) | This work |
| FYJ5340 | The triple mutant of *M. smegmatis* (Δ*bioH1*/*2*/3, ΔMSMEG_2036/ΔMSMEG_1352/ΔMSMEG_6710) | This work |
| FYJ5341 | FYJ5340, the Δ*bioH1*/*2*/3 triple mutant carrying pMV261-*bioH1*(MSMEG_2036) | This work |
| FYJ5342 | FYJ5340, the Δ*bioH1*/*2*/3 triple mutant carrying pMV261-*bioH2*(MSMEG_1352) | This work |
| FYJ5343 | FYJ5340, the Δ*bioH1*/*2*/3 triple mutant carrying pMV261-*bioH3*(MSMEG_6710) | This work |
| FYJ5344 | FYJ5340, the Δ*bioH1*/*2*/3 triple mutant carrying pMV261-BCG_3195c(*bioH1*) | This work |
| FYJ5345 | FYJ5340, the Δ*bioH1*/*2*/3 triple mutant carrying pMV261-BCG_0695c(*bioH2*) | This work |
| FYJ5346 | FYJ5340, the Δ*bioH1*/*2*/3 triple mutant carrying pMV261-BCG_2728 | This work |
| Plasmids |  |  |
| pBAD322 | An expression vector with the arabinose-inducible promoter; Amp^R^ | Lab stock |
| pET28a | A T7 promoter expression vector; Km^R^ | Lab stock |
| pMind | A tetracycline-inducible plasmid for gene silencing; Km^R^ | Lab stock ^4^ |
| pGoAL17 | pBR322 replicon, used for gene-knockout, Amp^R^ | This work |
| pMind-*bioAFD*UD-*sacB*-*lacZ* | The suicide plasmid used for *bioAFD*-knockout | This work |
| pMind-*bioH1*UD-*sacB*-*lacZ* | The suicide plasmid used for *bioH1*-knockout | This work |
| pMind-*bioH2*UD-*sacB*-*lacZ* | The suicide plasmid used for *bioH2*-knockout | This work |
| pMind-*bioH3*UD-*sacB*-*lacZ* | The suicide plasmid used for *bioH3*-knockout | This work |
| pMV261 | A shuttle vector between *E. coli* and *Mycobacteria* | Lab stock ^5,6^ |
| pMV261-*bioH1*(MSMEG_2036) | pMV261 carrying *bioH1*(MSMEG_2036) | This work |
| pMV261-*bioH2*(MSMEG_1352) | pMV261 encoding *bioH2*(MSMEG_1352) | This work |
| pMV261-*bioH3*(MSMEG_6710) | pMV261 harboring *bioH3*(MSMEG_6710) | This work |
| pMV261-BCG_3195c(*bioH1*) | pMV261 bearing BCG_3195c(*bioH1*) | This work |
| pMV261-BCG_0695c(*bioH2*) | pMV261 carrying BCG_0695c(*bioH2*) | This work |
| pMV261-BCG_2728 | pMV261 containing BCG_2728 | This work |
| pBAD322*::*MSMEG_2036 | A pBAD322 carrying *bioH1*(MSMEG_2036) at SmaI and SphI cuts; Amp^R^ | This work |
| pBAD322*::*MSMEG_1352 | A pBAD322 carrying *bioH2*(MSMEG_1352) at SmaI and SphI cuts; Amp^R^ | This work |
| pBAD322*::*MSMEG_6710 | A pBAD322 carrying *bioH3*(MSMEG_6710) at SmaI and SphI cuts; Amp^R^ | This work |
| pBAD322*::*MSMEG_0117 | A pBAD322 carrying MSMEG_0117 at SmaI and SphI cuts; Amp^R^ | This work |
| pBAD322*::*MSMEG_0267 | A pBAD322 carrying MSMEG_0267 at SmaI and SphI cuts; Amp^R^ | This work |
| pBAD322*::*MSMEG_0280 | A pBAD322 carrying MSMEG_0280 at SmaI and SphI cuts; Amp^R^ | This work |
| pBAD322*::*MSMEG_0289 | A pBAD322 carrying MSMEG_0289 at SmaI and SphI cuts; Amp^R^ | This work |
| pBAD322*::*MSMEG_0302 | A pBAD322 carrying MSMEG_0302 at SmaI and SphI cuts; Amp^R^ | This work |
| pBAD322*::*MSMEG_0460 | A pBAD322 carrying MSMEG_0460 at SmaI and SphI cuts; Amp^R^ | This work |
| pBAD322*::*MSMEG_0605 | A pBAD322 carrying MSMEG_0605 at SmaI and SphI cuts; Amp^R^ | This work |
| pBAD322*::*MSMEG_1108 | A pBAD322 carrying MSMEG_1108 at SmaI and SphI cuts; Amp^R^ | This work |
| pBAD322*::*MSMEG_1570 | A pBAD322 carrying MSMEG_1570 at SmaI and SphI cuts; Amp^R^ | This work |
| pBAD322*::*MSMEG_1576 | A pBAD322 carrying MSMEG_1576 at SmaI and SphI cuts; Amp^R^ | This work |
| pBAD322*::*MSMEG_1587 | A pBAD322 carrying MSMEG_1587 at SmaI and SphI cuts; Amp^R^ | This work |
| pBAD322*::*MSMEG_1655 | A pBAD322 carrying MSMEG_1655 at SmaI and SphI cuts; Amp^R^ | This work |
| pBAD322*::*MSMEG_1797 | A pBAD322 carrying MSMEG_1797 at SmaI and SphI cuts; Amp^R^ | This work |
| pBAD322*::*MSMEG_1940 | A pBAD322 carrying MSMEG_1940 at SmaI and SphI cuts; Amp^R^ | This work |
| pBAD322*::*MSMEG_1984 | A pBAD322 carrying MSMEG_1984 at SmaI and SphI cuts; Amp^R^ | This work |
| pBAD322*::*MSMEG_1998 | A pBAD322 carrying MSMEG_1998 at SmaI and SphI cuts; Amp^R^ | This work |
| pBAD322*::*MSMEG_2074 | A pBAD322 carrying MSMEG_2074 at SmaI and SphI cuts; Amp^R^ | This work |
| pBAD322*::*MSMEG_2203 | A pBAD322 carrying MSMEG_2203 at SmaI and SphI cuts; Amp^R^ | This work |
| pBAD322*::*MSMEG_2409 | A pBAD322 carrying MSMEG_2409 at SmaI and SphI cuts; Amp^R^ | This work |
| pBAD322*::*MSMEG_2517 | A pBAD322 carrying MSMEG_2517 at SmaI and SphI cuts; Amp^R^ | This work |
| pBAD322*::*MSMEG_2534 | A pBAD322 carrying MSMEG_2534 at SmaI and SphI cuts; Amp^R^ | Lab stock |
| pBAD322*::*MSMEG_2568 | A pBAD322 carrying MSMEG_2568 at SmaI and SphI cuts; Amp^R^ | Lab stock |
| pBAD322*::*MSMEG_2612 | A pBAD322 carrying MSMEG_2612 at SmaI and SphI cuts; Amp^R^ | Lab stock |
| pBAD322*::*MSMEG_2745 | A pBAD322 carrying MSMEG_2745 at SmaI and SphI cuts; Amp^R^ | This work |
| pBAD322*::*MSMEG_2767 | A pBAD322 carrying MSMEG_2767 at SmaI and SphI cuts; Amp^R^ | This work |
| pBAD322*::*MSMEG_2777 | A pBAD322 carrying MSMEG_2777 at SmaI and SphI cuts; Amp^R^ | This work |
| pBAD322*::*MSMEG_2875 | A pBAD322 carrying MSMEG_2875 at SmaI and SphI cuts; Amp^R^ | This work |
| pBAD322*::*MSMEG_2888 | A pBAD322 carrying MSMEG_2888 at SmaI and SphI cuts; Amp^R^ | This work |
| pBAD322*::*MSMEG_2900 | A pBAD322 carrying MSMEG_2900 at SmaI and SphI cuts; Amp^R^ | This work |
| pBAD322*::*MSMEG_2984 | A pBAD322 carrying MSMEG_2984 at SmaI and SphI cuts; Amp^R^ | This work |
| pBAD322*::*MSMEG_3010 | A pBAD322 carrying MSMEG_3010 at SmaI and SphI cuts; Amp^R^ | This work |
| pBAD322*::*MSMEG_3059 | A pBAD322 carrying MSMEG_3059 at SmaI and SphI cuts; Amp^R^ | This work |
| pBAD322*::*MSMEG_3287 | A pBAD322 carrying MSMEG_3287 at SmaI and SphI cuts; Amp^R^ | This work |
| pBAD322*::*MSMEG_3289 | A pBAD322 carrying MSMEG_3289 at SmaI and SphI cuts; Amp^R^ | This work |
| pBAD322*::*MSMEG_3297 | A pBAD322 carrying MSMEG_3297 at SmaI and SphI cuts; Amp^R^ | This work |
| pBAD322*::*MSMEG_3336 | A pBAD322 carrying MSMEG_3336 at SmaI and SphI cuts; Amp^R^ | This work |
| pBAD322*::*MSMEG_3508 | A pBAD322 carrying MSMEG_3508 at SmaI and SphI cuts; Amp^R^ | This work |
| pBAD322*::*MSMEG_3829 | A pBAD322 carrying MSMEG_3829 at SmaI and SphI cuts; Amp^R^ | This work |
| pBAD322*::*MSMEG_3842 | A pBAD322 carrying MSMEG_3842 at SmaI and SphI cuts; Amp^R^ | This work |
| pBAD322*::*MSMEG_3856 | A pBAD322 carrying MSMEG_3856 at SmaI and SphI cuts; Amp^R^ | This work |
| pBAD322*::*MSMEG_3891 | A pBAD322 carrying MSMEG_3891 at SmaI and SphI cuts; Amp^R^ | This work |
| pBAD322*::*MSMEG_3979 | A pBAD322 carrying MSMEG_3979 at SmaI and SphI cuts; Amp^R^ | This work |
| pBAD322*::*MSMEG_4148 | A pBAD322 carrying MSMEG_4148 at SmaI and SphI cuts; Amp^R^ | This work |
| pBAD322*::*MSMEG_4202 | A pBAD322 carrying MSMEG_4202 at SmaI and SphI cuts; Amp^R^ | This work |
| pBAD322*::*MSMEG_4295 | A pBAD322 carrying MSMEG_4295 at SmaI and SphI cuts; Amp^R^ | This work |
| pBAD322*::*MSMEG_4450 | A pBAD322 carrying MSMEG_4450 at SmaI and SphI cuts; Amp^R^ | This work |
| pBAD322*::*MSMEG_4707 | A pBAD322 carrying MSMEG_4707 at SmaI and SphI cuts; Amp^R^ | This work |
| pBAD322*::*MSMEG_4810 | A pBAD322 carrying MSMEG_4810 at SmaI and SphI cuts; Amp^R^ | This work |
| pBAD322*::*MSMEG_4815 | A pBAD322 carrying MSMEG_4815 at SmaI and SphI cuts; Amp^R^ | This work |
| pBAD322*::*MSMEG_4860 | A pBAD322 carrying MSMEG_4860 at SmaI and SphI cuts; Amp^R^ | This work |
| pBAD322*::*MSMEG_4867 | A pBAD322 carrying MSMEG_4867 at SmaI and SphI cuts; Amp^R^ | This work |
| pBAD322*::*MSMEG_4998 | A pBAD322 carrying MSMEG_4998 at SmaI and SphI cuts; Amp^R^ | This work |
| pBAD322*::*MSMEG_5107 | A pBAD322 carrying MSMEG_5107 at SmaI and SphI cuts; Amp^R^ | This work |
| pBAD322*::*MSMEG_5171 | A pBAD322 carrying MSMEG_5171 at SmaI and SphI cuts; Amp^R^ | This work |
| pBAD322*::*MSMEG_5209 | A pBAD322 carrying MSMEG_5209 at SmaI and SphI cuts; Amp^R^ | This work |
| pBAD322*::*MSMEG_5210 | A pBAD322 carrying MSMEG_5210 at SmaI and SphI cuts; Amp^R^ | This work |
| pBAD322*::*MSMEG_5220 | A pBAD322 carrying MSMEG_5220 at SmaI and SphI cuts; Amp^R^ | This work |
| pBAD322*::*MSMEG_5271 | A pBAD322 carrying MSMEG_5271 at SmaI and SphI cuts; Amp^R^ | This work |
| pBAD322*::*MSMEG_5278 | A pBAD322 carrying MSMEG_5278 at SmaI and SphI cuts; Amp^R^ | This work |
| pBAD322*::*MSMEG_5341 | A pBAD322 carrying MSMEG_5341 at SmaI and SphI cuts; Amp^R^ | This work |
| pBAD322*::*MSMEG_5350 | A pBAD322 carrying MSMEG_5350 at SmaI and SphI cuts; Amp^R^ | This work |
| pBAD322*::*MSMEG_5652 | A pBAD322 carrying MSMEG_5652 at SmaI and SphI cuts; Amp^R^ | This work |
| pBAD322*::*MSMEG_5763 | A pBAD322 carrying MSMEG_5763 at SmaI and SphI cuts; Amp^R^ | This work |
| pBAD322*::*MSMEG_5823 | A pBAD322 carrying MSMEG_5823 at SmaI and SphI cuts; Amp^R^ | This work |
| pBAD322*::*MSMEG_5878 | A pBAD322 carrying MSMEG_5878 at SmaI and SphI cuts; Amp^R^ | This work |
| pBAD322*::*MSMEG_6029 | A pBAD322 carrying MSMEG_6029 at SmaI and SphI cuts; Amp^R^ | This work |
| pBAD322*::*MSMEG_6037 | A pBAD322 carrying MSMEG_6037 at SmaI and SphI cuts; Amp^R^ | This work |
| pBAD322*::*MSMEG_6085 | A pBAD322 carrying MSMEG_6085 at SmaI and SphI cuts; Amp^R^ | This work |
| pBAD322*::*MSMEG_6106 | A pBAD322 carrying MSMEG_6106 at SmaI and SphI cuts; Amp^R^ | This work |
| pBAD322*::*MSMEG_6151 | A pBAD322 carrying MSMEG_6151 at SmaI and SphI cuts; Amp^R^ | This work |
| pBAD322*::*MSMEG_6184 | A pBAD322 carrying MSMEG_6184 at SmaI and SphI cuts; Amp^R^ | This work |
| pBAD322*::*MSMEG_6381 | A pBAD322 carrying MSMEG_6381 at SmaI and SphI cuts; Amp^R^ | This work |
| pBAD322*::*MSMEG_6533 | A pBAD322 carrying MSMEG_6533 at SmaI and SphI cuts; Amp^R^ | This work |
| pBAD322*::*MSMEG_6586 | A pBAD322 carrying MSMEG_6586 at SmaI and SphI cuts; Amp^R^ | This work |
| pBAD322*::*MSMEG_6597 | A pBAD322 carrying MSMEG_6597 at SmaI and SphI cuts; Amp^R^ | This work |
| pBAD322*::*MSMEG_6658 | A pBAD322 carrying MSMEG_6658 at SmaI and SphI cuts; Amp^R^ | This work |
| pBAD322*::*MSMEG_6708 | A pBAD322 carrying MSMEG_6708 at SmaI and SphI cuts; Amp^R^ | This work |
| pBAD322*::*MSMEG_6719 | A pBAD322 carrying MSMEG_6719 at SmaI and SphI cuts; Amp^R^ | This work |
| pBAD322*::*MSMEG_6720 | A pBAD322 carrying MSMEG_6720 at SmaI and SphI cuts; Amp^R^ | This work |
| pBAD322*::*MSMEG_6763 | A pBAD322 carrying MSMEG_6763 at SmaI and SphI cuts; Amp^R^ | This work |
| pBAD322*::*MSMEG_6772 | A pBAD322 carrying MSMEG_6772 at SmaI and SphI cuts; Amp^R^ | This work |
| pBAD322*::*MSMEG_6831 | A pBAD322 carrying MSMEG_6831 at SmaI and SphI cuts; Amp^R^ | This work |
| pBAD322*::*MSMEG_6838 | A pBAD322 carrying MSMEG_6838 at SmaI and SphI cuts; Amp^R^ | This work |
| pBAD322*::*MSMEG_6850 | A pBAD322 carrying MSMEG_6850 at SmaI and SphI cuts; Amp^R^ | This work |
| pBAD322*::*MSMEG_6852 | A pBAD322 carrying MSMEG_6852 at SmaI and SphI cuts; Amp^R^ | This work |
| pBAD322*::*MSMEG_6906 | A pBAD322 carrying MSMEG_6906 at SmaI and SphI cuts; Amp^R^ | This work |
| pBAD322*::*BCG_3195c | A pBAD322 carrying BCG_3195c at SmaI and SphI cuts; Amp^R^ | This work |
| pBAD322*::*BCG_3195c (S110A) | A pBAD322 carrying BCG_3195c(S110A) at SmaI and SphI cuts; Amp^R^ | This work |
| pBAD322*::*BCG_3195c (D263A) | A pBAD322 carrying BCG_3195c(D263A) at SmaI and SphI cuts; Amp^R^ | This work |
| pBAD322*::*BCG_3195c (H291A) | A pBAD322 carrying BCG_3195c(H291A) at SmaI and SphI cuts; Amp^R^ | This work |
| pBAD322*::*BCG_0695c | A pBAD322 carrying BCG_0695c at SmaI and SphI cuts; Amp^R^ | This work |
| pBAD322*::*BCG_2728 | A pBAD322 carrying BCG_2728 at SmaI and SphI cuts; Amp^R^ | This work |
| pBAD322*::*MSMEG_2036 (S110A) | pBAD322 encoding MSMEG_2036 (S110A); Amp^R^ | This work |
| pBAD322*::*MSMEG_2036 (D251A) | pBAD322 encoding MSMEG_2036 (D251A); Amp^R^ | This work |
| pBAD322*::*MSMEG_2036 (H279A) | pBAD322 encoding MSMEG_2036 (H279A); Amp^R^ | This work |
| pBAD322*::*MSMEG_1352 (S127A) | pBAD322 encoding MSMEG_1352 (S127A); Amp^R^ | This work |
| pBAD322*::*MSMEG_1352 (D255A) | pBAD322 encoding MSMEG_1352 (D255A); Amp^R^ | This work |
| pBAD322*::*MSMEG_1352 (H283A) | pBAD322 encoding MSMEG_1352 (H283A); Amp^R^ | This work |
| pBAD322*::*MSMEG_6710 (S103A) | pBAD322 encoding MSMEG_6710 (S103A); Amp^R^ | This work |
| pBAD322*::*MSMEG_6710 (D232A) | pBAD322 encoding MSMEG_6710 (D232A); Amp^R^ | This work |
| pBAD322*::*MSMEG_6710 (H265A) | pBAD322 encoding MSMEG_6710 (H265A); Amp^R^ | This work |
| pBAD322*::*MSMEG_6710 (R134A) | pBAD322 encoding MSMEG_6710 (R134A); Amp^R^ | This work |
| pBAD322*::*MSMEG_6710 (R136A) | pBAD322 encoding MSMEG_6710 (R136A); Amp^R^ | This work |
| pBAD322*::*MSMEG_6710 (R180A) | pBAD322 encoding MSMEG_6710 (R180A); Amp^R^ | This work |
| pBAD322*::*MSMEG_6710 (R181A) | pBAD322 encoding MSMEG_6710 (R181A); Amp^R^ | This work |
| pBAD322*::*MSMEG_6710 (R196A) | pBAD322 encoding MSMEG_6710 (R196A); Amp^R^ | This work |
| pBAD322*::*MSMEG_6710 (R217A) | pBAD322 encoding MSMEG_6710 (R217A); Amp^R^ | This work |
| pBAD322*::*MSMEG_6710 (R218A) | pBAD322 encoding MSMEG_6710 (R218A); Amp^R^ | This work |
| pBAD322*::*MSMEG_6710 (R221A) | pBAD322 encoding MSMEG_6710 (R221A); Amp^R^ | This work |
| pBAD322*::*MSMEG_6710 (R233A) | pBAD322 encoding MSMEG_6710 (R233A); Amp^R^ | This work |
| pBAD322*::*MSMEG_6710 (R249A) | pBAD322 encoding MSMEG_6710 (R249A); Amp^R^ | This work |
| pBAD322*::*MSMEG_6710 (R217A/R218A) | pBAD322 encoding MSMEG_6710 (R217A/R218A); Amp^R^ | This work |
| pBAD322*::*MSMEG_6710 (R217A/R218A/R221A) | pBAD322 encoding MSMEG_6710 (R217A/R218A/R221A); Amp^R^ | This work |
| pBAD322*::*MSMEG_6710 (R217A/R218A/R221A/R249A) | pBAD322 encoding MSMEG_6710 (R217A/R218A/R221A/R249A); Amp^R^ | This work |
| pET28a*::*MSMEG_2036 | pET28a encoding MSMEG_2036; Km^R^ | This work |
| pET28a*::*MSMEG_1352 | pET28a encoding MSMEG_1352; Km^R^ | This work |
| pET28a*::*MSMEG_6710 | pET28a encoding MSMEG_6710; Km^R^ | This work |
| pET28a*::*MSMEG_2036 (S110A) | pET28a encoding MSMEG_2036(S110A); Km^R^ | This work |
| pET28a*::*MSMEG_2036 (D251A) | pET28a encoding MSMEG_2036(D251A); Km^R^ | This work |
| pET28a*::*MSMEG_2036 (H279A) | pET28a encoding MSMEG_2036(H279A); Km^R^ | This work |
| pET28a*::*MSMEG_1352 (S127A) | pET28a encoding MSMEG_1352 (S127A); Km^R^ | This work |
| pET28a*::*MSMEG_1352 (D255A) | pET28a encoding MSMEG_1352 (D255A); Km^R^ | This work |
| pET28a*::*MSMEG_1352 (H283A) | pET28a encoding MSMEG_1352 (H283A); Km^R^ | This work |
| pET28a*::*MSMEG_6710 (S103A) | pET28a encoding MSMEG_6710 (S103A); Km^R^ | This work |
| pET28a*::*MSMEG_6710 (D232A) | pET28a encoding MSMEG_6710 (D232A); Km^R^ | This work |
| pET28a*::*MSMEG_6710 (H265A) | pET28a encoding MSMEG_6710 (H265A); Km^R^ | This work |
| pET28a*::*BCG_3195c | pET28a encoding BCG_3195c; Km^R^ | This work |
| pET28a*::*BCG_0695c | pET28a encoding BCG_0695c; Km^R^ | This work |
| pET28a*::*BCG_2728 | pET28a encoding BCG_2728; Km^R^ | This work |

**Supplementary references**

1. Feng, Y., Zhang, H. & Cronan, J.E. Profligate biotin synthesis in alpha-proteobacteria - a developing or degenerating regulatory system? *Mol Microbiol* **88**, 77-92 (2013).

2. Lin, S., Hanson, R.E. & Cronan, J.E. Biotin synthesis begins by hijacking the fatty acid synthetic pathway. *Nat Chem Biol* **6**, 682-8 (2010).

3. Lin, S. & Cronan, J.E. The BioC O-methyltransferase catalyzes methyl esterification of malonyl-acyl carrier protein, an essential step in biotin synthesis. *J Biol Chem* **287**, 37010-20 (2012).

4. Blokpoel, M.C. et al. Tetracycline-inducible gene regulation in mycobacteria. *Nucleic Acids Res* **33**, e22 (2005).

5. Stover, C.K. et al. New use of BCG for recombinant vaccines. *Nature* **351**, 456-60 (1991).

6. Cooksey, R.C., Crawford, J.T., Jacobs, W.R., Jr. & Shinnick, T.M. A rapid method for screening antimicrobial agents for activities against a strain of *Mycobacterium tuberculosis* expressing firefly luciferase. *Antimicrob Agents Chemother* **37**, 1348-52 (1993).
