# Supplementary material for: Three enigmatic BioH isoenzymes are programmed in the early stage of mycobacterial biotin synthesis, an attractive anti-TB drug target
Source: PLoS Pathog. 2022 Jul 11;18(7):e1010615. doi: 10.1371/journal.ppat.1010615 (PMC9302846; doi:10.1371/journal.ppat.1010615)
Supplement: S4 Table — (DOCX) [file ppat.1010615.s004.docx]

**Table S4** A list of DNA oligos used in this study

| Primers | Primer sequences (5’-3’) |
| --- | --- |
| RT-PCR | |
| *16S* rDNA*-*F1 | GGA ATA TTG CAC AAT GGG CG |
| *16S* rDNA*-*R1 | GCC CAC AGT TAA GCT GTG AG |
| MSMEG_2036-F1 | CAA ACT TTC GGA CCC GAG GAC |
| MSMEG_2036-R1 | TTG TGC AGC GGA ATG TGC TT |
| MSMEG_1352-F1 | ATC TTC TCC AGC AAC AAC CAG G |
| MSMEG_1352-R1 | GGA AGT TCG TGA CCC ATC CC |
| MSMEG_6710-F1 | AGC ATT GCC GTC GAT CTT CC |
| MSMEG_6710-R1 | CGG ATT GTC GGG CGT GAG T |
| Real-time qPCR |  |
| *sigA-*F | CGA GGA AGA AGA AGC TGA TG |
| *sigA-*R | CGT CTT TGC GTG CCT GTC |
| MSMEG_2036-F2 | CGC AAA CTT TCG GAC CCG AGG AC |
| MSMEG_2036-R2 | CCG CGA GGT GGT TGA GGC TGT A |
| MSMEG_1352-F2 | GAT GCT GAT GTG GCG TAC CGA GTT |
| MSMEG_1352-R2 | GGG CTG CGC TTA CCG AGG AA |
| MSMEG_6710-F2 | CGT GGC GCT GCC CAT CTT CT |
| MSMEG_6710-R2 | GCT GAG GCT CGC ATA CGG TTC G |
| Gene knockout | |
| pMind-MSMEG_2036-U-F(PacI) | CCTTAATTAACA GGC CGC ATC GGG GAA CGT GTG |
| MSMEG_2036-U-R | TTC AGC CGC TGC GGG CTG ATT AAC TGG GGA AGG GTC ATA CTG CT |
| MSMEG_2036-D-F | AGC AGT ATG ACC CTT CCC CAG TTA ATC AGC CCG CAG CGG CTG AA |
| pMind-MSMEG_2036-D-R(NheI) | CTAGCTAGC GAT GTG TCG CGG ATC CAG TG |
| pMind-*bioAFD*-U-F(PacI) | CCTTAATTAACA GTG CGC GGG TCG CTC ATC C |
| *bioAFD*-U-F | GCT CCT CCC GCA GAC GCC TCA CAC AGA CGG GGA GCC TAG CCC T |
| *bioAFD*-D-F | AGG GCT AGG CTC CCC GTC TGT GTG AGG CGT CTG CGG GAG GAG C |
| pMind-*bioAFD*-D-R(NheI) | CTAGCTAGC TCG AAG TGC GAA CCC TCC GGC T |
| PMind-MSMEG_1352-U-F(PacI) | CCTTAATTA AGC GCA ACC CGT GAG GTA GTG |
| MSMEG_1352-U-R | GAC AAG GCC TGA TGC AGA CCT GAT CAC CGG CCC GAT TTC |
| MSMEG-1352-D-F | CGA AA TCG GGC CGG TGA TCA GGT CTG CAT CAG GCC TTG T |
| pMind-MSMEG-1352-D-R(NheI) | CTAGCTAGC CGC TGC ACA GCG GCG AGG A |
| pMind-MSMEG_6710-U-F(PacI) | AGT CCA TAT GGA TAT CCTTAATTA AGC GGT TGC AGT GTC CGC TGC TG |
| MSMEG_6710-U-R | GGG CGG CTT TCT CGT GAG CGG TTG TAG GTC CTT TCG TTG TTC AGA C |
| MSMEG_6710-D-F | GTC TGA ACA ACG AAA GGA CCT ACA ACC GCT CAC GAG AAA GCC GCC C |
| pMind-MSMEG_6710-D-R(NheI) | GTG CTT GCG GCA GCG TGA AGC TAG CGC GGC GCC GCA GCG CGA TGA T |
| Gene expression and protein production | |
| pBAD322_MSMEG_2036-F | AGG AGG AAT TCA CCA TGG TAC CCG GGA TGA CCC TTC CCC AGT TAC |
| pBAD322_MSMEG_2036-R | AAG CTT GCA TGC CTG CAG GTC GAC TCA AGA GTT CAG CCG CTG |
| pBAD322_MSMEG_1352-F | AGG AGG AAT TCA CCA TGG TAC CCG GGA TGC AGA CCC GCA CCG GGC TC |
| pBAD322_MSMEG_1352-R | AAG CTT GCA TGC CTG CAG GTC GAC CTA ACC CAC CAC GGC AAA GTT TGT |
| pBAD322_MSMEG_6710-F | AGG AGG AAT TCA CCA TGG TAC CCG GGA TGC TGC ACG AGG AGA TCC GC |
| pBAD322_MSMEG_6710-R | CCG CCA AAA CAG CCA AGC TTG CAT GCC TAG CCG CAT GGG TTC GGC T |
| pBAD322_MSMEG_0117-F | TTT TGG GCT AGC AGG AGG AAT TCA TGA ACA TCC ATC ACC GCT AC |
| pBAD322_MSMEG_0117-R | AAG CTT GCA TGC CTG CAG GTC GAC TCA TGA AAC CTT CCT GTC CAG |
| pBAD322_MSMEG_0267-F | AGG AGG AAT TCA CCA TGG TAC CCG GGA TGC ATC GCC GCC GCA GCG CG |
| pBAD322_MSMEG_0267-R | AAG CTT GCA TGC CTG CAG GTC GAC TCA GGA GAC GTT CGC CAG C |
| pBAD322_MSMEG_0280-F | TTT TGG GCT AGC AGG AGG AAT TCA TGA CGG CCA CCA CCC CG |
| pBAD322_MSMEG_0280-R | AAG CTT GCA TGC CTG CAG GTC GAC TCA GGT GAG CGC ACG CTT G |
| pBAD322_MSMEG_0289-F | AGG AGG AAT TCA CCA TGG TAC CCG GGG TGA CGG CCA CCA CCC CGA CC |
| pBAD322_MSMEG_0289-R | AAG CTT GCA TGC CTG CAG GTC GAC TCA GGT GAG CGC ACG CTT GAG G |
| pBAD322_MSMEG_0302-F | AGG AGG AAT TCA CCA TGG TAC CCG GGA TGG CTA AAC CGC TGC TCA CC |
| pBAD322_MSMEG_0302-R | AAG CTT GCA TGC CTG CAG GTC GAC CTA GCG GTT CTG TGC CAC ACC GCG |
| pBAD322_MSMEG_0406-F | TTT TGG GCT AGC AGG AGG AAT TCA TGA GGC ATG CTG ACG AGG |
| pBAD322_MSMEG_0406-R | AAG CTT GCA TGC CTG CAG GTC GAC TCA TGT GAT CGA CCA GAA CTC |
| pBAD322_MSMEG_0605-F | TTT TGG GCT AGC AGG AGG AAT TCA TGA TCC GCG AGT TCG TCG |
| pBAD322_MSMEG_0605-R | AAG CTT GCA TGC CTG CAG GTC GAC TCA CCG CCA CCG CTT CGC |
| pBAD322_MSMEG_1108-F | AGG AGG AAT TCA CCA TGG TAC CCG GGG TGC GGC ACG TCG TAC CGC GTG |
| pBAD322_MSMEG_1108-R | CCG CCA AAA CAG CCA AGC TTG CAT GCC TAC AGC TTA TTC GCC AGC AGG TTG |
| pBAD322_MSMEG_1570-F | AGG AGG AAT TCA CCA TGG TAC CCG GGA TGA ACA TCG CAT CGC GAT GG |
| pBAD322_MSMEG_1570-R | AAG CTT GCA TGC CTG CAG GTC GAC CTA CCC GCG GCG AAC GCC GTC GAC |
| pBAD322_MSMEG_1576-F | TTT TGG GCT AGC AGG AGG AAT TCA TGG CCG GGC TGG CCG CTG T |
| pBAD322_MSMEG_1576-R | AAG CTT GCA TGC CTG CAG GTC GAC TCA CCC ATG GGG CCG TAC CC |
| pBAD322_MSMEG_1587-F | AGG AGG AAT TCA CCA TGG TAC CCG GGA TGA GCC GTC ACC ACC GAC G |
| pBAD322_MSMEG_1587-R | CCG CCA AAA CAG CCA AGC TTG CAT GCT CAA CCG TGG ACG CGA GTC AC |
| pBAD322_MSMEG_1655-F | TTT TGG GCT AGC AGG AGG AAT TCA TGG TTG AAC GTG CAC CCA T |
| pBAD322_MSMEG_1655-R | AAG CTT GCA TGC CTG CAG GTC GAC TCT TTG CAA TCT CGG CGT CC |
| pBAD322_MSMEG_1797-F | AGG AGG AAT TCA CCA TGG TAC CCG GGA TGG CTG ACT TCG TGA TCG TC |
| pBAD322_MSMEG_1797-R | AAG CTT GCA TGC CTG CAG GTC GAC CTA TGA CAC TCC CGC CTC GAC GAT |
| pBAD322_MSMEG_1940-F | AGG AGG AAT TCA CCA TGG TAC CCG GGA TGG CGA CAG ACG TGC TCC AT |
| pBAD322_MSMEG_1940-R | AAG CTT GCA TGC CTG CAG GTC GAC CTA GCC CAG CTG CCG GCG GAT CAA |
| pBAD322_MSMEG_1984-F | TTT TGG GCT AGC AGG AGG AAT TCA TGT TCT TTG GCT TCG ACG AAC |
| pBAD322_MSMEG_1984-R | AAG CTT GCA TGC CTG CAG GTC GAC CTA GGC GCA ATC ACC GAA GA |
| pBAD322_MSMEG_1998-F | AGG AGG AAT TCA CCA TGG TAC CCG GGA TGA CCG CCC CTG AGC GGC TC |
| pBAD322_MSMEG_1998-R | AAG CTT GCA TGC CTG CAG GTC GAC CTA CAA GTT CGC GTA ATC CGC GAT |
| pBAD322_MSMEG_2074-F | TTT TGG GCT AGC AGG AGG AAT TCA TGA CGA CGA CAA CCG AAG C |
| pBAD322_MSMEG_2074-R | AAG CTT GCA TGC CTG CAG GTC GAC TCA CTG CGC CGC ACC CTC |
| pBAD322_MSMEG_2203-F | AGG AGG AAT TCA CCA TGG TAC CCG GGA TGA CCT ACA CCG AGT TCA CGT TC |
| pBAD322_MSMEG_2203-R | CCG CCA AAA CAG CCA AGC TTG CAT GCT CAG CGC AGG GAC AGC GTG CG |
| pBAD322_MSMEG_2409-F | AGG AGG AAT TCA CCA TGG TAC CCG GGA TGC TGC TCG CGC ACG CGA CG |
| pBAD322_MSMEG_2409-R | AAG CTT GCA TGC CTG CAG GTC GAC CTA GAC GTG CCT GAG ATG TGC CC |
| pBAD322_MSMEG_2517-F | AGG AGG AAT TCA CCA TGG TAC CCG GGA TGA TCC GTT CAG AAG TCG ACC TGT |
| pBAD322_MSMEG_2517-R | CCG CCA AAA CAG CCA AGC TTG CAT GCT CAA GCT GTT GCG CTG ATC CG |
| pBAD322_MSMEG_2534-F | AGG AGG AAT TCA CCA TGG TAC CCG GGA TGC CCA ACC GGA TGT ACA TC |
| pBAD322_MSMEG_2534-R | AAG CTT GCA TGC CTG CAG GTC GAC CTA CGC GAT TCC GTT TCC CGC CCT |
| pBAD322_MSMEG_2568-F | TTT TGG GCT AGC AGG AGG AAT TCA TGG CTC ACA GGT CCG GAT |
| pBAD322_MSMEG_2568-R | AAG CTT GCA TGC CTG CAG GTC GAC TCA GCG CAG CGC CCG GC |
| pBAD322_MSMEG_2612-F | AGG AGG AAT TCA CCA TGG TAC CCG GGA TGA GTG CGA TCG GTC ACT TC |
| pBAD322_MSMEG_2612-R | AAG CTT GCA TGC CTG CAG GTC GAC CTA CCT ATC CCG AAC GAG TCG TTG |
| pBAD322_MSMEG_2745-F | AGG AGG AAT TCA CCA TGG TAC CCG GGA TGA TCC ACG GGA TCG GCG AC |
| pBAD322_MSMEG_2745-R | AAG CTT GCA TGC CTG CAG GTC GAC CTA GGT TGC GCT GCG CTC GTC GA |
| pBAD322_MSMEG_2767-F | AGG AGG AAT TCA CCA TGG TAC CCG GGG TGA TGA GCG TCA TCC TGC GCG |
| pBAD322_MSMEG_2767-R | CCG CCA AAA CAG CCA AGC TTG CAT GCT CAG ACG TCA CCC AGC GCG GCC |
| pBAD322_MSMEG_2777-F | TTT TGG GCT AGC AGG AGG AAT TC A TGC ACG ATG AAC TGC AGC G |
| pBAD322_MSMEG_2777-R | AAG CTT GCA TGC CTG CAG GTC GAC TCA GAG CGC GAC GAC GCG GT |
| pBAD322_MSMEG_2875-F | TTT TGG GCT AGC AGG AGG AAT TCA TGA CCA CCT CAC CAC CGA |
| pBAD322_MSMEG_2875-R | AAG CTT GCA TGC CTG CAG GTC GAC TCA GCC CGC GAA GGG CAG |
| pBAD322_MSMEG_2888-F | TTT TGG GCT AGC AGG AGG AAT TCA TGA GTG GGC TCG ATC CGC |
| pBAD322_MSMEG_2888-R | AAG CTT GCA TGC CTG CAG GTC GAC TTA CGG CGC GGC GAT TCC |
| pBAD322_MSMEG_2900-F | AGG AGG AAT TCA CCA TGG TAC CCG GGA TGC AGG CGA TCG ACT ACC AG |
| pBAD322_MSMEG_2900-R | AAG CTT GCA TGC CTG CAG GTC GAC CTA GTT GTC CTG CGT CAG GAA CGA |
| pBAD322_MSMEG_2913-F | AGG AGG AAT TCA CCA TGG TAC CCG GGA TGC GCC CCG ATG TGG ACT GG |
| pBAD322_MSMEG_2913-R | CCG CCA AAA CAG CCA AGC TTG CAT GCT CAT CCA ACT GAA CGG TTG ACG C |
| pBAD322_MSMEG_2984-F | AGG AGG AAT TCA CCA TGG TAC CCG GGA TGA TTC TCA CGC ACG CAG AG |
| pBAD322_MSMEG_2984-R | AAG CTT GCA TGC CTG CAG GTC GAC CTA GGT CCC TTC GGC GTG CCG GAC |
| pBAD322_MSMEG_3010-F | AGG AGG AAT TCA CCA TGG TAC CCG GGA TGA TAC AGG CGT GCG TCA TT |
| pBAD322_MSMEG_3010-R | AAG CTT GCA TGC CTG CAG GTC GAC CTA GAC GGC AAG GGG TTT GGC GGA |
| pBAD322_MSMEG_3059-F | AGG AGG AAT TCA CCA TGG TAC CCG GGA TGT CTG TTG CTG ACG AGA AAC |
| pBAD322_MSMEG_3059-R | AAG CTT GCA TGC CTG CAG GTC GAC CTA GCT GTG CAG CGC CTC GCG AAG T |
| pBAD322_MSMEG_3087-F | TTT TGG GCT AGC AGG AGG AAT TCA TGG ACT CCT ACC GCC GCG G |
| pBAD322_MSMEG_3087-R | AAG CTT GCA TGC CTG CAG GTC GAC TCA CAC CGG GTG CGC CGC GA |
| pBAD322_MSMEG_3089-F | AGG AGG AAT TCA CCA TGG TAC CCG GGA TGA GCA GGG TTG CGG GGC TGG |
| pBAD322_MSMEG_3089-R | AAG CTT GCA TGC CTG CAG GTC GAC CTA GGC CGT GGC GCG CTC GGG TGT C |
| pBAD322_MSMEG_3097-F | AGG AGG AAT TCA CCA TGG TAC CCG GGA TGC CGC CGC TGC GCC AGC AC |
| pBAD322_MSMEG_3097-R | AAG CTT GCA TGC CTG CAG GTC GAC CTA GGG CTC GGC CAG GAA CGC CTC |
| pBAD322_MSMEG_3336-F | AGG AGG AAT TCA CCA TGG TAC CCG GGA TGA CTG AAG GAT TGC GAG CTG |
| pBAD322_MSMEG_3336-R | AAG CTT GCA TGC CTG CAG GTC GAC CTA GAC GCT CAC CAC CGA CGT CAG C |
| pBAD322_MSMEG_3508-F | TTT TGG GCT AGC AGG AGG AAT TCA TGG CCA TGG TCA TCC CGA T |
| pBAD322_MSMEG_3508-R | AAG CTT GCA TGC CTG CAG GTC GAC CTA CGT CAA CGC GGA CGT CT |
| pBAD322_MSMEG_3829-F | TTT TGG GCT AGC AGG AGG AAT TCA TGC CGA TCG ATC CCA TTG C |
| pBAD322_MSMEG_3829-R | AAG CTT GCA TGC CTG CAG GTC GAC TCA TCG GGT CGA CTG CCC GG |
| pBAD322_MSMEG_3842-F | TTT TGG GCT AGC AGG AGG AAT TCA TGA CCA CCT TCG TCC TTG TCC C |
| pBAD322_MSMEG_3842-R | AAG CTT GCA TGC CTG CAG GTC GAC CTA TCG GGA GGC GAC GTC GAC |
| pBAD322_MSMEG_3856-F | TTT TGG GCT AGC AGG AGG AAT TCA TGT GGC GTT TCG ATG ATT TCG T |
| pBAD322_MSMEG_3856-R | AAG CTT GCA TGC CTG CAG GTC GAC TCA GGA CCC GTC GAG GGC GAG |
| pBAD322_MSMEG_3891-F | TTT TGG GCT AGC AGG AGG AAT TCA TGT CGT TGC GCA GCG CAC |
| pBAD322_MSMEG_3891-R | AAG CTT GCA TGC CTG CAG GTC GAC TTA ATT TGG CGC CCG GCC G |
| pBAD322_MSMEG_3979-F | AGG AGG AAT TCA CCA TGG TAC CCG GGA TGC TAC TGC CCC ATG ACG AAT CAG |
| pBAD322_MSMEG_3979-R | CCG CCA AAA CAG CCA AGC TTG CAT GCA TGC TAC TGC CCC ATG ACG AAT CAG |
| pBAD322_MSMEG_4148-F | TTT TGG GCT AGC AGG AGG AAT TCA TGA CGT TGG ATG AAC CGA CGA |
| pBAD322_MSMEG_4148-R | AAG CTT GCA TGC CTG CAG GTC GAC TCA CCA GCA GTT GGC CAG GGC |
| pBAD322_MSMEG_4202-F | TTT TGG GCT AGC AGG AGG AAT TCA TGA CTG CCC AGA TCG CCC CT |
| pBAD322_MSMEG_4202-R | AAG CTT GCA TGC CTG CAG GTC GAC TCA GTT GAG GCC ACC GGC AGT |
| pBAD322_MSMEG_4295-F | TTT TGG GCT AGC AGG AGG AAT TCA TGC TGC CCG CGC TGA TGA CT |
| pBAD322_MSMEG_4295-R | AAG CTT GCA TGC CTG CAG GTC GAC CTA GCA GGC AAG GTT GCC CGG |
| pBAD322_MSMEG_4450-F | TTT TGG GCT AGC AGG AGG AAT TCA TGG CTC AAC CGC TCA GCT ACA |
| pBAD322_MSMEG_4450-R | AAG CTT GCA TGC CTG CAG GTC GAC TCA TCG GGT GCC AGC TCC GAC |
| pBAD322_MSMEG_4707-F | AGG AGG AAT TCA CCA TGG TAC CCG GGG TGG CTG CCA TCA CGG CCT ACC |
| pBAD322_MSMEG_4707-R | CCG CCA AAA CAG CCA AGC TTG CAT GCT CAG CGC GCC GCA GCA AGA T |
| pBAD322_MSMEG_4810-F | TTT TGG GCT AGC AGG AGG AAT TCA TGA CGG CAA CAG GTA GCA CGG |
| pBAD322_MSMEG_4810-R | AAG CTT GCA TGC CTG CAG GTC GAC TCA CTT GAG GAA CTC CAG GAT GGC |
| pBAD322_MSMEG_4815-F | TTT TGG GCT AGC AGG AGG AAT TCA TGG CGA GTT CGG ACG GGA CCG |
| pBAD322_MSMEG_4815-R | AAG CTT GCA TGC CTG CAG GTC GAC TCA ACC TGC CTC CAC TTC CAC A |
| pBAD322_MSMEG_4860-F | AGG AGG AAT TCA CCA TGG TAC CCG GGG TGG ACT TCG AAC GTA AGA CCA TCG |
| pBAD322_MSMEG_4860-R | CCG CCA AAA CAG CCA AGC TTG CAT GCT CAT GAC ACC TCT TTC AGG AAG TCC |
| pBAD322_MSMEG_4867-F | AGG AGG AAT TCA CCA TGG TAC CCG GGA TGA CAA GCA CTG CCG CGG CAG |
| pBAD322_MSMEG_4867-R | CCG CCA AAA CAG CCA AGC TTG CAT GCT CAC CGC CCG AGC AGG AAG TCG AGG |
| pBAD322_MSMEG_4998-F | AGG AGG AAT TCA CCA TGG TAC CCG GGA TGG CAA ACC ACG GTC GCA AGC |
| pBAD322_MSMEG_4998-R | CCG CCA AAA CAG CCA AGC TTG CAT GCT CAT GAA GTA GCG GGC AGC ACG |
| pBAD322_MSMEG_5107-F | AGG AGG AAT TCA CCA TGG TAC CCG GGG TGA ACA TCG TCC TGG TCC AC |
| pBAD322_MSMEG_5107-R | CCG CCA AAA CAG CCA AGC TTG CAT GCT CAG ACG TGA ACG GCC TGC CCG |
| pBAD322_MSMEG_5171-F | AGG AGG AAT TCA CCA TGG TAC CCG GGA TGG CGA CGA TCG ACG GCA AC |
| pBAD322_MSMEG_5171-R | CCG CCA AAA CAG CCA AGC TTG CAT GCT CAC TCG GTC AGC AGG TCG TC |
| pBAD322_MSMEG_5209-F | AGG AGG AAT TCA CCA TGG TAC CCG GGG TGA GCA CTG CAC AAA CCG AAT |
| pBAD322_MSMEG_5209-R | CCG CCA AAA CAG CCA AGC TTG CAT GCT CAC CGG TTC ACG AAC TCC ACG |
| pBAD322_MSMEG_5210-F | AGG AGG AAT TCA CCA TGG TAC CCG GGA TGA CTG TGG TTT TGG TGC ACG |
| pBAD322_MSMEG_5210-R | CCG CCA AAA CAG CCA AGC TTG CAT GCT CAC CGA GGG CTG GTA GCC CAG |
| pBAD322_MSMEG_5220-F | AGG AGG AAT TCA CCA TGG TAC CCG GGA TGA GAT TTG AGC TGT CGG CAT T |
| pBAD322_MSMEG_5220-R | CCG CCA AAA CAG CCA AGC TTG CAT GCC TAC GAC CAT ATG CCG ACG CTC |
| pBAD322_MSMEG_5271-F | AGG AGG AAT TCA CCA TGG TAC CCG GGG TGG TGG CGA TGA CCG CAC CG |
| pBAD322_MSMEG_5271-R | CCG CCA AAA CAG CCA AGC TTG CAT GCT CAC CAC GTC GCC TCA CGT ATG T |
| pBAD322_MSMEG_5278-F | AGG AGG AAT TCA CCA TGG TAC CCG GGG TGA CGG GCA CCA AGA CCA CC |
| pBAD322_MSMEG_5278-R | CCG CCA AAA CAG CCA AGC TTG CAT GCT CAG GCG CTC TCG TTG TTG GTG |
| pBAD322_MSMEG_5341-F | TTT TGG GCT AGC AGG AGG AAT TCA TGA AGT TCC TGT TCG ACG AC |
| pBAD322_MSMEG_5341-R | AAG CTT GCA TGC CTG CAG GTC GAC CCC GCC GAC GAT TTC GTC GA |
| pBAD322_MSMEG_5350-F | TTT TGG GCT AGC AGG AGG AAT TCA TGA TTA GCT GGC GCA AAC AAC |
| pBAD322_MSMEG_5350-R | AAG CTT GCA TGC CTG CAG GTC GAC CTC GGA GTC GGA CTC GGA CG |
| pBAD322_MSMEG_5652-F | TTT TGG GCT AGC AGG AGG AAT TCA TGG GCA GTG CAC CCG GCG T |
| pBAD322_MSMEG_5652-R | AAG CTT GCA TGC CTG CAG GTC GAC AGG CAG GAA ATC CAG CAG CG |
| pBAD322_MSMEG_5652-F | AGG AGG AAT TCA CCA TGG TAC CCG GGA TGA TGC GGC CGT CCC TGC TCG |
| pBAD322_MSMEG_5652-R | CCG CCA AAA CAG CCA AGC TTG CAT GCT CAG GTG AGG TAC CCG GCG AC |
| pBAD322_MSMEG_5763-F | AGG AGG AAT TCA CCA TGG TAC CCG GGA TGA TGC GGC CGT CCC TGC TCG |
| pBAD322_MSMEG_5763-R | CCG CCA AAA CAG CCA AGC TTG CAT GCT CAG GTG AGG TAC CCG GCG AC |
| pBAD322_MSMEG_5823-F | AGG AGG AAT TCA CCA TGG TAC CCG GGG TGA TCG AGG AGC ACT CAC ACA CC |
| pBAD322_MSMEG_5823-R | CCG CCA AAA CAG CCA AGC TTG CAT GCT CAG TAT TCG GGT CCG TCG TTG |
| pBAD322_MSMEG_5878-F | AGG AGG AAT TCA CCA TGG TAC CCG GGA TGA ACG TTC TCA AAT TGT TGG G |
| pBAD322_MSMEG_5878-R | AGG AGG AAT TCA CCA TGG TAC CCG GGA TGA TAC AGG CGT GCG TCA TT |
| pBAD322_MSMEG_6029-F | AAG CTT GCA TGC CTG CAG GTC GAC CTA GAC GGC AAG GGG TTT GGC GGA |
| pBAD322_MSMEG_6029-R | AGG AGG AAT TCA CCA TGG TAC CCG GGA TGT CTG TTG CTG ACG AGA AAC |
| pBAD322_MSMEG_6037-F | AAG CTT GCA TGC CTG CAG GTC GAC CTA GCT GTG CAG CGC CTC GCG AAG T |
| pBAD322_MSMEG_6037-R | TTT TGG GCT AGC AGG AGG AAT TCA TGG ACT CCT ACC GCC GCG G |
| pBAD322_MSMEG_6085-F | AAG CTT GCA TGC CTG CAG GTC GAC TCA CAC CGG GTG CGC CGC GA |
| pBAD322_MSMEG_6085-R | AGG AGG AAT TCA CCA TGG TAC CCG GGA TGA GCA GGG TTG CGG GGC TGG |
| pBAD322_MSMEG_6106-F | AAG CTT GCA TGC CTG CAG GTC GAC CTA GGC CGT GGC GCG CTC GGG TGT C |
| pBAD322_MSMEG_6106-R | AGG AGG AAT TCA CCA TGG TAC CCG GGA TGC CGC CGC TGC GCC AGC AC |
| pBAD322_MSMEG_6151-F | AAG CTT GCA TGC CTG CAG GTC GAC CTA GGG CTC GGC CAG GAA CGC CTC |
| pBAD322_MSMEG_6151-R | AGG AGG AAT TCA CCA TGG TAC CCG GGA TGA TAC AGG CGT GCG TCA TT |
| pBAD322_MSMEG_6184-F | AAG CTT GCA TGC CTG CAG GTC GAC CTA GAC GGC AAG GGG TTT GGC GGA |
| pBAD322_MSMEG_6184-R | AGG AGG AAT TCA CCA TGG TAC CCG GGA TGT CTG TTG CTG ACG AGA AAC |
| pBAD322_MSMEG_6381-F | AAG CTT GCA TGC CTG CAG GTC GAC CTA GCT GTG CAG CGC CTC GCG AAG T |
| pBAD322_MSMEG_6381-R | TTT TGG GCT AGC AGG AGG AAT TCA TGG ACT CCT ACC GCC GCG G |
| pBAD322_MSMEG_6533-F | AAG CTT GCA TGC CTG CAG GTC GAC TCA CAC CGG GTG CGC CGC GA |
| pBAD322_MSMEG_6533-R | AGG AGG AAT TCA CCA TGG TAC CCG GGA TGA GCA GGG TTG CGG GGC TGG |
| pBAD322_MSMEG_6586-F | AAG CTT GCA TGC CTG CAG GTC GAC CTA GGC CGT GGC GCG CTC GGG TGT C |
| pBAD322_MSMEG_6586-R | AGG AGG AAT TCA CCA TGG TAC CCG GGA TGC CGC CGC TGC GCC AGC AC |
| pBAD322_MSMEG_6597-F | AAG CTT GCA TGC CTG CAG GTC GAC CTA GGG CTC GGC CAG GAA CGC CTC |
| pBAD322_MSMEG_6597-R | AGG AGG AAT TCA CCA TGG TAC CCG GGA TGA CTG AAG GAT TGC GAG CTG |
| pBAD322_MSMEG_6658-F | AAG CTT GCA TGC CTG CAG GTC GAC CTA GAC GCT CAC CAC CGA CGT CAG C |
| pBAD322_MSMEG_6658-R | TTT TGG GCT AGC AGG AGG AAT TCA TGG CCA TGG TCA TCC CGA T |
| pBAD322_MSMEG_6708-F | AAG CTT GCA TGC CTG CAG GTC GAC CTA CGT CAA CGC GGA CGT CT |
| pBAD322_MSMEG_6708-R | TTT TGG GCT AGC AGG AGG AAT TCA TGC CGA TCG ATC CCA TTG C |
| pBAD322_MSMEG_6719-F | AAG CTT GCA TGC CTG CAG GTC GAC TCA TCG GGT CGA CTG CCC GG |
| pBAD322_MSMEG_6719-R | TTT TGG GCT AGC AGG AGG AAT TCA TGA CCA CCT TCG TCC TTG TCC C |
| pBAD322_MSMEG_6720-F | AAG CTT GCA TGC CTG CAG GTC GAC CTA TCG GGA GGC GAC GTC GAC |
| pBAD322_MSMEG_6720-R | TTT TGG GCT AGC AGG AGG AAT TCA TGT GGC GTT TCG ATG ATT TCG T |
| pBAD322_MSMEG_6763-F | AAG CTT GCA TGC CTG CAG GTC GAC TCA GGA CCC GTC GAG GGC GAG |
| pBAD322_MSMEG_6763-R | TTT TGG GCT AGC AGG AGG AAT TCA TGT CGT TGC GCA GCG CAC |
| pBAD322_MSMEG_6763-F | AAG CTT GCA TGC CTG CAG GTC GAC TTA ATT TGG CGC CCG GCC G |
| pBAD322_MSMEG_6772-R | AGG AGG AAT TCA CCA TGG TAC CCG GGA TGC TAC TGC CCC ATG ACG AAT CAG |
| pBAD322_MSMEG_6831-F | CCG CCA AAA CAG CCA AGC TTG CAT GCA TGC TAC TGC CCC ATG ACG AAT CAG |
| pBAD322_MSMEG_6831-R | TTT TGG GCT AGC AGG AGG AAT TCA TGA CGT TGG ATG AAC CGA CGA |
| pBAD322_MSMEG_6838-F | AAG CTT GCA TGC CTG CAG GTC GAC TCA CCA GCA GTT GGC CAG GGC |
| pBAD322_MSMEG_6838-R | TTT TGG GCT AGC AGG AGG AAT TCA TGA CTG CCC AGA TCG CCC CT |
| pBAD322_MSMEG_6850-F | AAG CTT GCA TGC CTG CAG GTC GAC TCA GTT GAG GCC ACC GGC AGT |
| pBAD322_MSMEG_6850-R | TTT TGG GCT AGC AGG AGG AAT TCA TGC TGC CCG CGC TGA TGA CT |
| pBAD322_MSMEG_6852-F | AAG CTT GCA TGC CTG CAG GTC GAC CTA GCA GGC AAG GTT GCC CGG |
| pBAD322_MSMEG_6852-R | TTT TGG GCT AGC AGG AGG AAT TCA TGG CTC AAC CGC TCA GCT ACA |
| pBAD322_MSMEG_6906-F | AAG CTT GCA TGC CTG CAG GTC GAC TCA TCG GGT GCC AGC TCC GAC |
| pBAD322_MSMEG_6906-R | AGG AGG AAT TCA CCA TGG TAC CCG GGG TGG CTG CCA TCA CGG CCT ACC |
| MEMEG_2036 (S110A)-F | CCG CCA AAA CAG CCA AGC TTG CAT GCT CAG CGC GCC GCA GCA AGA T |
| MEMEG_2036 (S110A)-R | TTT TGG GCT AGC AGG AGG AAT TCA TGA CGG CAA CAG GTA GCA CGG |
| MEMEG_2036 (D251A)-F | AAG CTT GCA TGC CTG CAG GTC GAC TCA CTT GAG GAA CTC CAG GAT GGC |
| MEMEG_2036 (D251A)-R | TTT TGG GCT AGC AGG AGG AAT TCA TGG CGA GTT CGG ACG GGA CCG |
| MEMEG_2036 (H279A)-F | AAG CTT GCA TGC CTG CAG GTC GAC TCA ACC TGC CTC CAC TTC CAC A |
| MEMEG_2036 (H279A)-R | AGG AGG AAT TCA CCA TGG TAC CCG GGG TGG ACT TCG AAC GTA AGA CCA TCG |
| MEMEG_1352 (S127A)-F | CCG CCA AAA CAG CCA AGC TTG CAT GCT CAT GAC ACC TCT TTC AGG AAG TCC |
| MEMEG_1352 (S127A)-R | AGG AGG AAT TCA CCA TGG TAC CCG GGA TGA CAA GCA CTG CCG CGG CAG |
| MEMEG_1352 (D255A)-F | CCG CCA AAA CAG CCA AGC TTG CAT GCT CAC CGC CCG AGC AGG AAG TCG AGG |
| MEMEG_1352 (D255A)-R | AGG AGG AAT TCA CCA TGG TAC CCG GGA TGG CAA ACC ACG GTC GCA AGC |
| MEMEG_1352 (H283A)-F | CCG CCA AAA CAG CCA AGC TTG CAT GCT CAT GAA GTA GCG GGC AGC ACG |
| MEMEG_1352 (H283A)-R | AGG AGG AAT TCA CCA TGG TAC CCG GGG TGA ACA TCG TCC TGG TCC AC |
| MEMEG_6710 (S103A)-F | AGG AGG AAT TCA CCA TGG TAC CCG GGA TGA TAC AGG CGT GCG TCA TT |
| MEMEG_6710 (S103A)-R | AAG CTT GCA TGC CTG CAG GTC GAC CTA GAC GGC AAG GGG TTT GGC GGA |
| MEMEG_6710 (D248A)-F | AGG AGG AAT TCA CCA TGG TAC CCG GGA TGT CTG TTG CTG ACG AGA AAC |
| MEMEG_6710 (D248A)-R | AAG CTT GCA TGC CTG CAG GTC GAC CTA GCT GTG CAG CGC CTC GCG AAG T |
| MEMEG_6710 (H265A)-F | TTT TGG GCT AGC AGG AGG AAT TCA TGG ACT CCT ACC GCC GCG G |
| MEMEG_6710 (H265A)-R | AAG CTT GCA TGC CTG CAG GTC GAC TCA CAC CGG GTG CGC CGC GA |
| MEMEG_6710 (R180A)-F | AGG AGG AAT TCA CCA TGG TAC CCG GGA TGA GCA GGG TTG CGG GGC TGG |
| MEMEG_6710 (R180A)-R | AGG AGG AAT TCA CCA TGG TAC CCG GGA TGA TAC AGG CGT GCG TCA TT |
| MEMEG_6710 (R181A)-F | AAG CTT GCA TGC CTG CAG GTC GAC CTA GAC GGC AAG GGG TTT GGC GGA |
| MEMEG_6710 (R181A)-R | AGG AGG AAT TCA CCA TGG TAC CCG GGA TGT CTG TTG CTG ACG AGA AAC |
| MEMEG_6710 (R196A)-F | AAG CTT GCA TGC CTG CAG GTC GAC CTA GCT GTG CAG CGC CTC GCG AAG T |
| MEMEG_6710 (R196A)-R | TTT TGG GCT AGC AGG AGG AAT TCA TGG ACT CCT ACC GCC GCG G |
| MEMEG_6710 (R233A)-F | AAG CTT GCA TGC CTG CAG GTC GAC TCA CAC CGG GTG CGC CGC GA |
| MEMEG_6710 (R233A)-R | AGG AGG AAT TCA CCA TGG TAC CCG GGA TGA GCA GGG TTG CGG GGC TGG |
| MEMEG_6710 (R134A)-F | AAG CTT GCA TGC CTG CAG GTC GAC CTA GGC CGT GGC GCG CTC GGG TGT C |
| MEMEG_6710 (R134A)-R | AGG AGG AAT TCA CCA TGG TAC CCG GGA TGA TAC AGG CGT GCG TCA TT |
| MEMEG_6710 (R136A)-F | AAG CTT GCA TGC CTG CAG GTC GAC CTA GAC GGC AAG GGG TTT GGC GGA |
| MEMEG_6710 (R136A)-R | AGG AGG AAT TCA CCA TGG TAC CCG GGA TGT CTG TTG CTG ACG AGA AAC |
| MEMEG_6710 (R217A)-F | AAG CTT GCA TGC CTG CAG GTC GAC CTA GCT GTG CAG CGC CTC GCG AAG T |
| MEMEG_6710 (R217A)-R | TTT TGG GCT AGC AGG AGG AAT TCA TGG ACT CCT ACC GCC GCG G |
| MEMEG_6710 (R218A)-F | AAG CTT GCA TGC CTG CAG GTC GAC TCA CAC CGG GTG CGC CGC GA |
| MEMEG_6710 (R218A)-R | AGG AGG AAT TCA CCA TGG TAC CCG GGA TGA GCA GGG TTG CGG GGC TGG |
| MEMEG_6710 (R221A)-F | AAG CTT GCA TGC CTG CAG GTC GAC CTA GGC CGT GGC GCG CTC GGG TGT C |
| MEMEG_6710 (R221A)-R | AGG AGG AAT TCA CCA TGG TAC CCG GGA TGA TAC AGG CGT GCG TCA TT |
| MEMEG_6710 (R249A)-F | AAG CTT GCA TGC CTG CAG GTC GAC CTA GAC GGC AAG GGG TTT GGC GGA |
| MEMEG_6710 (R249A)-R | AGG AGG AAT TCA CCA TGG TAC CCG GGA TGT CTG TTG CTG ACG AGA AAC |
| pET28a-MSMEG_2036-F | AAG CTT GCA TGC CTG CAG GTC GAC CTA GCT GTG CAG CGC CTC GCG AAG T |
| pET28a-MSMEG_2036-R | TTT TGG GCT AGC AGG AGG AAT TCA TGG ACT CCT ACC GCC GCG G |
| pET21a-MSMEG_1352-F | AAG CTT GCA TGC CTG CAG GTC GAC TCA CAC CGG GTG CGC CGC GA |
| pET21a-MSMEG_1352-R | AGG AGG AAT TCA CCA TGG TAC CCG GGA TGA GCA GGG TTG CGG GGC TGG |
| pET28a-MSMEG_6710-F | AAG CTT GCA TGC CTG CAG GTC GAC CTA GGC CGT GGC GCG CTC GGG TGT C |
| pET28a-MSMEG_6710-R | AGG AGG AAT TCA CCA TGG TAC CCG GGA TGC CGC CGC TGC GCC AGC AC |
| Genetic complementation | |
| pMV261-MSMEG_2036-F(BamHI) | GCA ATG GCC AAG ACA ATT GCG GAT CCA ATG ACC CTT CCC CAG TTA CCT CCG |
| pMV261-MSMEG_2036-R(SalI) | ATC GTA CGC TAG TTA ACT ACG TCG AC T CAA GAG TTC AGC CGC TGC GGG |
| pMV261-MSMEG_1352-F(BamHI) | GCA ATG GCC AAG ACA ATT GCG GAT CCA ATG CAG ACC CGC ACC GGG CTC G |
| pMV261-MSMEG_1352-R(SalI) | ATC GTA CGC TAG TTA ACT ACG TCG AC T CAA CCC ACC ACG GCA AAG TTT GTC |
| pMV261-MSMEG_6710-F(BamHI) | GCA ATG GCC AAG ACA ATT GCG GAT CCA GTG AAC GAT TCC CGG AAT TCC G |
| pMV261-MSMEG_6710-R(SalI) | ATC GTA CGC TAG TTA ACT ACG TCG AC TCA GAT GCC GGC CAG CAG GTC |
| pMV261-BCG_3195c-F(BamHI) | GCA ATG GCC AAG ACA ATT GCG GAT CCA ATG TTG ACC GTC CGT GCG GCG GAC |
| pMV261-BCG_3195c-R(SalI) | ATC GTA CGC TAG TTA ACT ACG TCG ACC TAT GAG CTG ATC CGC CGG TCC C |
| pMV261-BCG_0695c-F(BamHI) | GCA ATG GCC AAG ACA ATT GCG GAT CCA ATG GAC ATC CGT AGC GGC ACC G |
| pMV261-BCG_0695c-R(SalI) | ATC GTA CGC TAG TTA ACT ACG TCG ACT CAG CCG GCC TCA GAG AAG TTG C |
| pMV261-BCG_2728-F(BamHI) | GCA ATG GCC AAG ACA ATT GCG GAT CCA ATG ACC GAG CGG AAG CGA AAT CT |
| pMV261_BCG_2728-R(SalI) | ATC GTA CGC TAG TTA ACT ACG TCG ACT CAG GTA GCG CTG CGT TCG TTG |

*The letters underlined denote restriction cuts.
