# Supplementary figures and images for: Three enigmatic BioH isoenzymes are programmed in the early stage of mycobacterial biotin synthesis, an attractive anti-TB drug target
Source: PLoS Pathog. 2022 Jul 11;18(7):e1010615. doi: 10.1371/journal.ppat.1010615 (PMC9302846; doi:10.1371/journal.ppat.1010615)

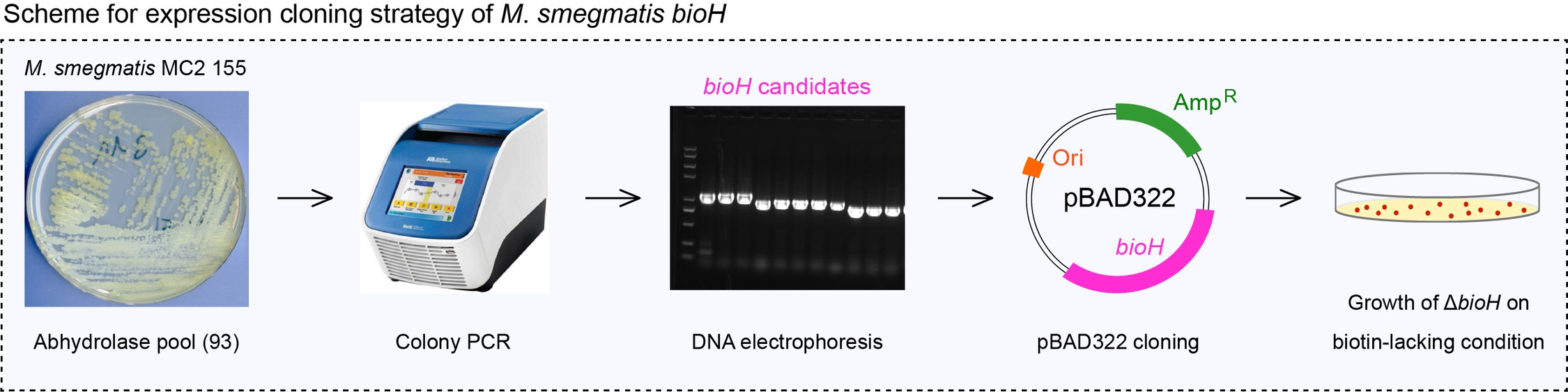

Supplement: S1 Fig — (TIF) [file ppat.1010615.s005.tif]

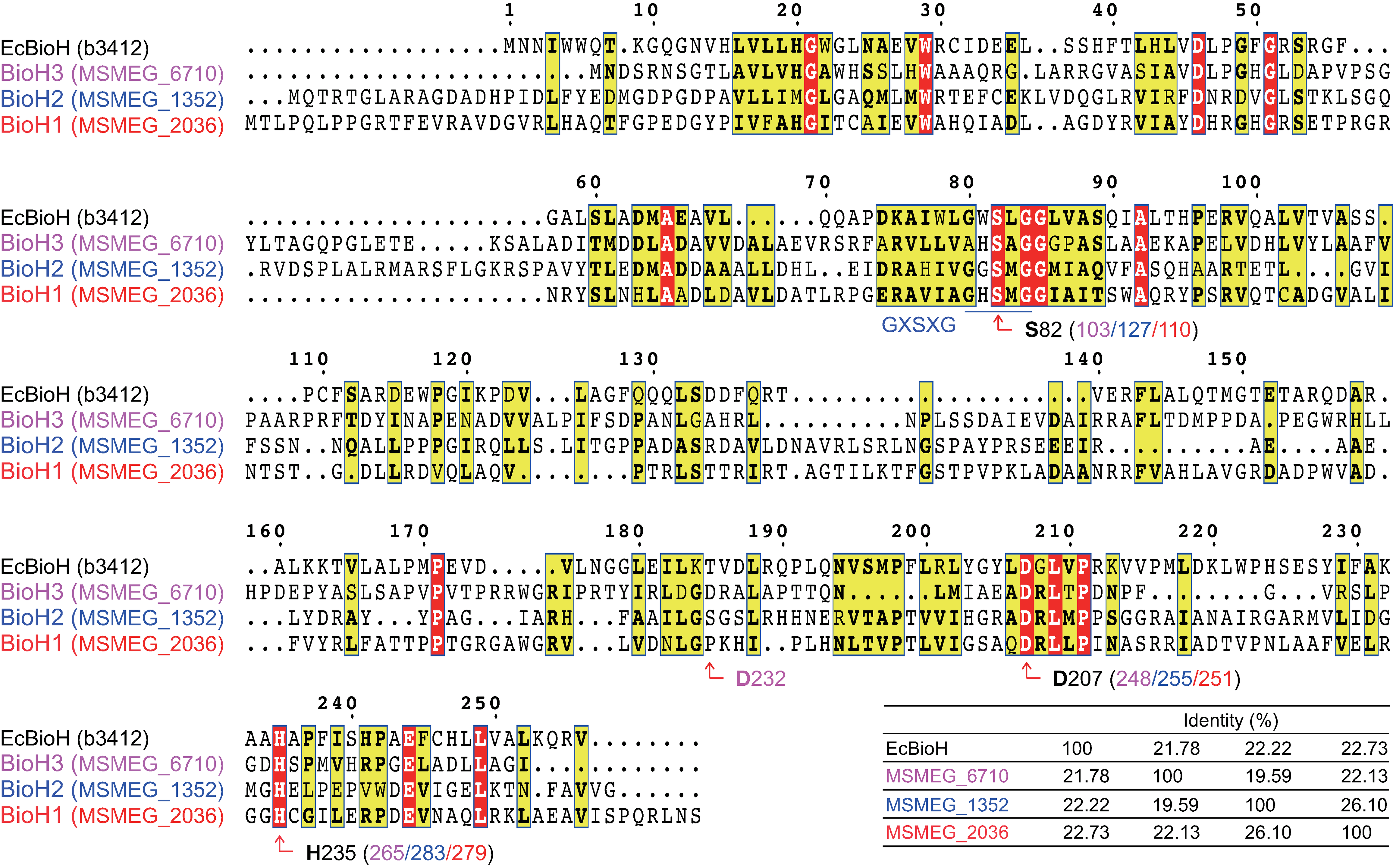

Supplement: S2 Fig — Inside table displays identity across different BioH members. The residues of catalytic triad are labeled with arrows. Namely, they included i) S82, D207 & H235 (EcBioH); ii) S110, D251 & H279 (BioH1); iii) S127, D255 & H283 (BioH2); and iv) S103, D232 & H265 (BioH3). Of note, crystal structure of BioH3 argued that the conserved D248 participates into catalytic triad. In contrast, it is replaced with D232. Clustal Omega (https://www.ebi.ac.uk/Tools/msa/clustalo/) was used to conduct sequence alignment. Identical residues are indicated with white letters in red background, similar sites are shown with dark letters in yellow background, different residues are indicated with black letters, and gaps are denoted with dots. (TIF) [file ppat.1010615.s006.tif]

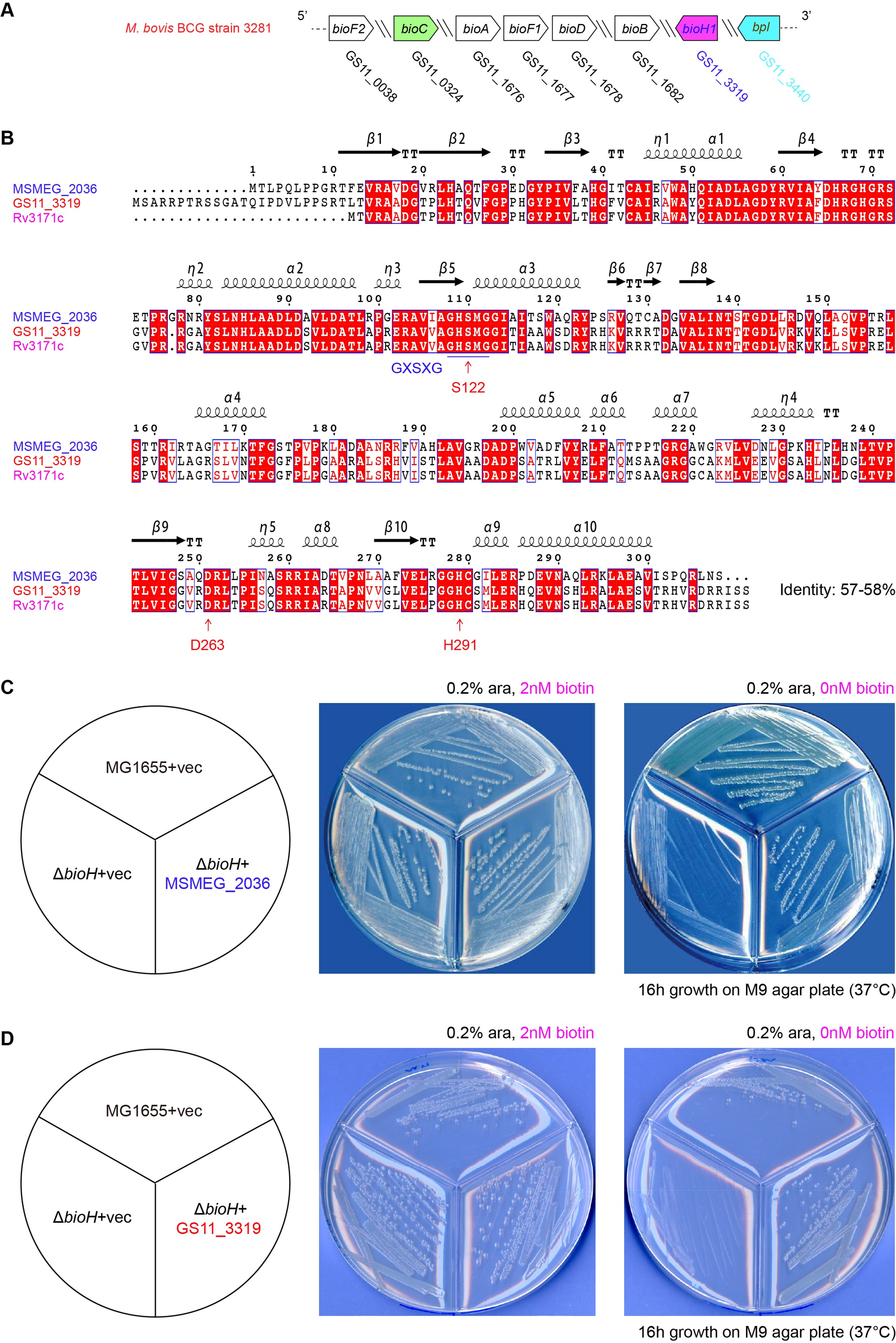

Supplement: S3 Fig — A. Genomic environment of bioH and other bio-related loci in M. bovis BCG strain 3281. B. Sequence alignment of GS11_3319 of M. bovis BCG with the counterpart of M. smegmatis. Compared with MSMEG_2036, the identity of GS11_3319 (and/or Rv3171c) is estimated to 57–58%. The three residues forming catalytic triad of GS11_3319 are labeled, namely S122, D263, and H291. C. The presence of MSMEG_2036 of M. smegmatis renders the ΔbioH biotin auxotrophic strain to appear on the non-permissive growth condition defective in biotin. D. Functional expression of GS11_3319 of M. bovis BCG allows growth of the ΔbioH biotin auxotrophic strain on the biotin-lacking condition. Designations: α, α-helices; β, β-sheet; T, T-turn; η, coils; vec, pBAD24 vector; ara, arabinose; bpl, biotin protein ligase. (TIF) [file ppat.1010615.s007.tif]

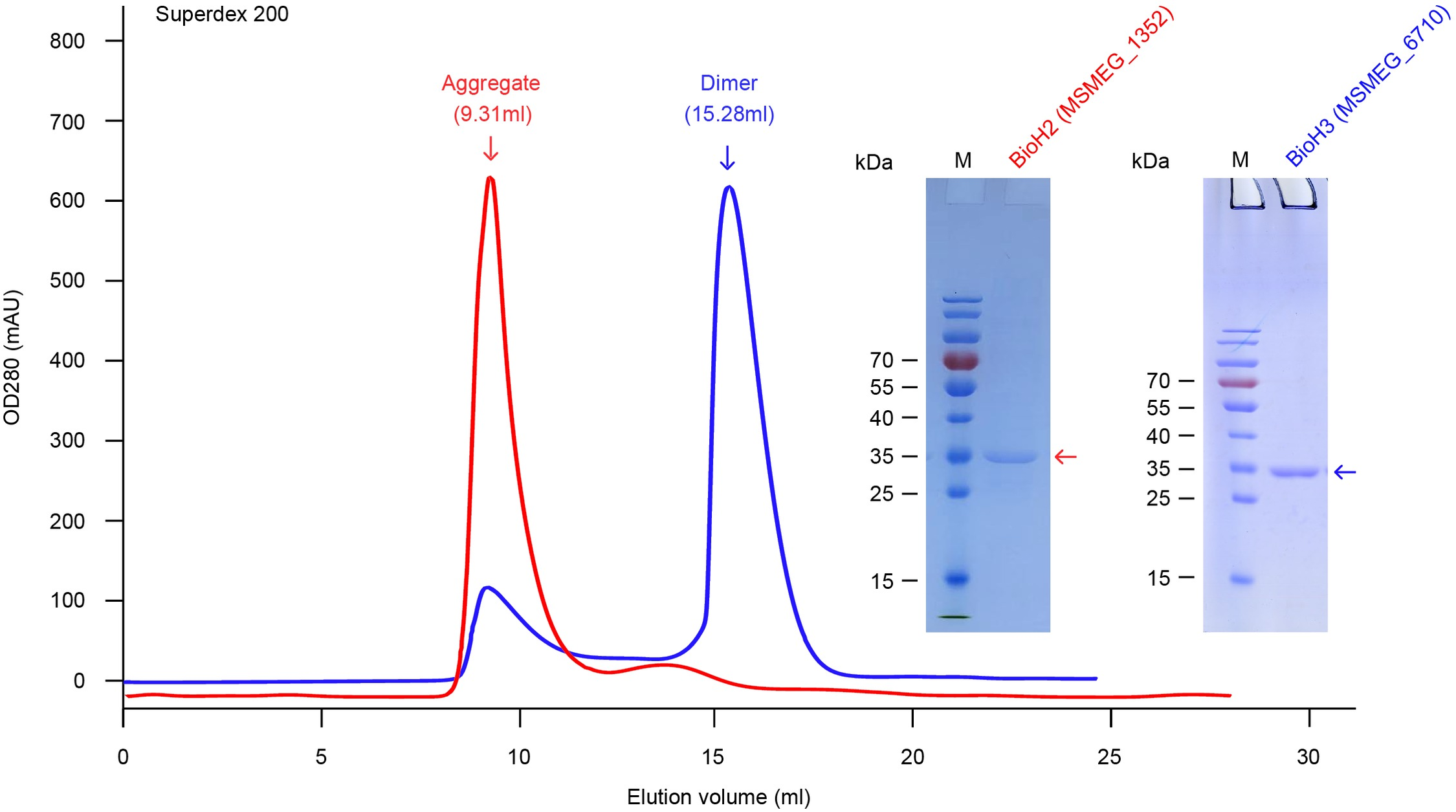

Supplement: S4 Fig — Unlike BioH3 (MSMEG_6710) forming a dimer, gel filtration analysis revealed that BioH2(MSMEG_1352) behaves as a soluble aggregate. The inside gels denote SDS-PAGE profiles of both BioH2 and BioH3. Gel filtration assays were performed with a Superdex 200 Increase column (of note, this is a 6-year-used column of which protein elution volume is little bit lagged when compared with the brand new one). (TIF) [file ppat.1010615.s008.tif]

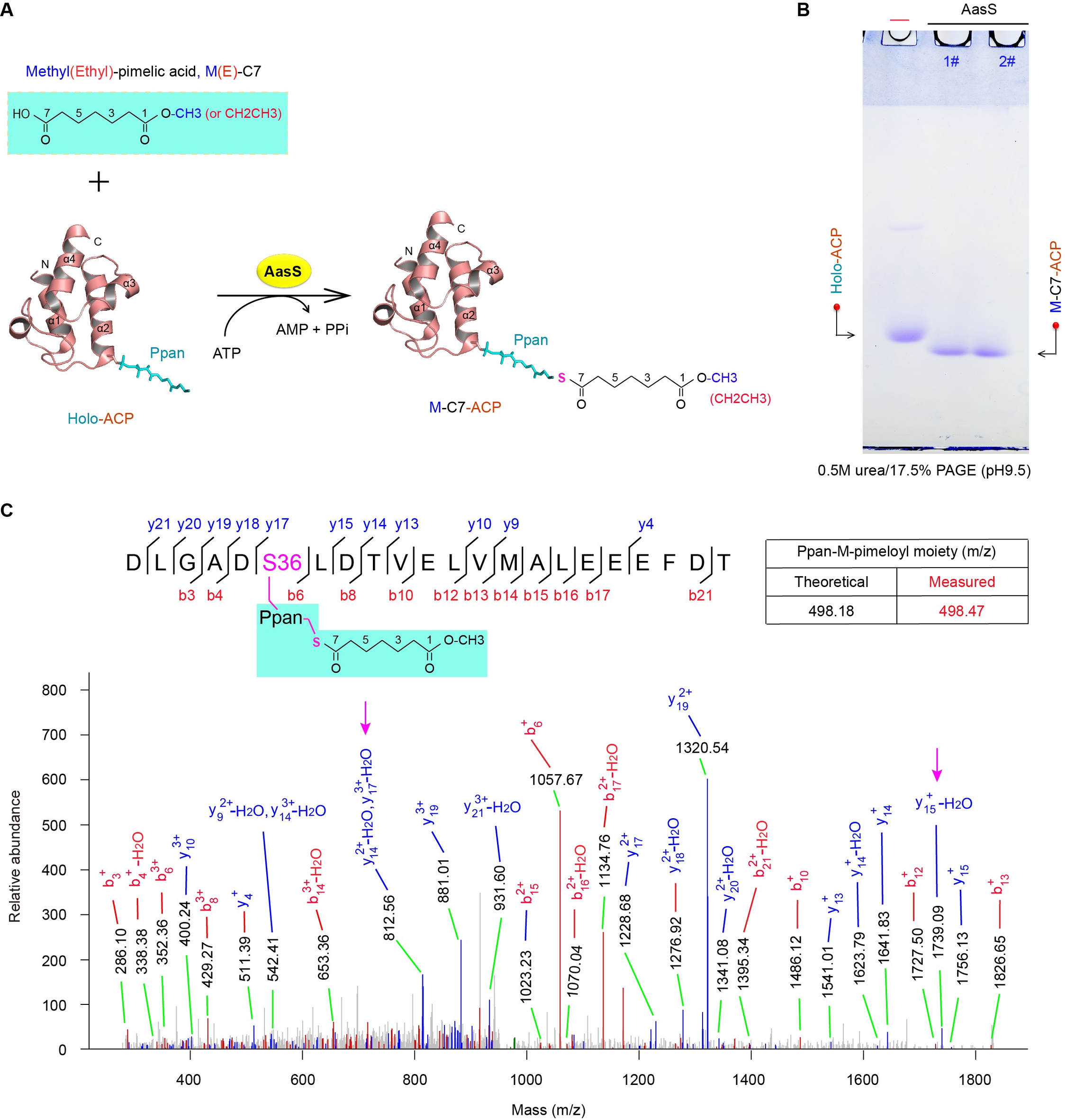

Supplement: S5 Fig — A. Representative diagram for AasS-based synthesis of M(E)-C7-ACP. Designations: M-C7-ACP, Methyl pimeloyl-ACP; E-C7-ACP, Ethyl pimeloyl-ACP. B. AasS catalyzes the synthesis of M-C7-ACP in vitro. C. MS identification for an ACP-arising peptide with M-C7 modification. (TIF) [file ppat.1010615.s009.tif]

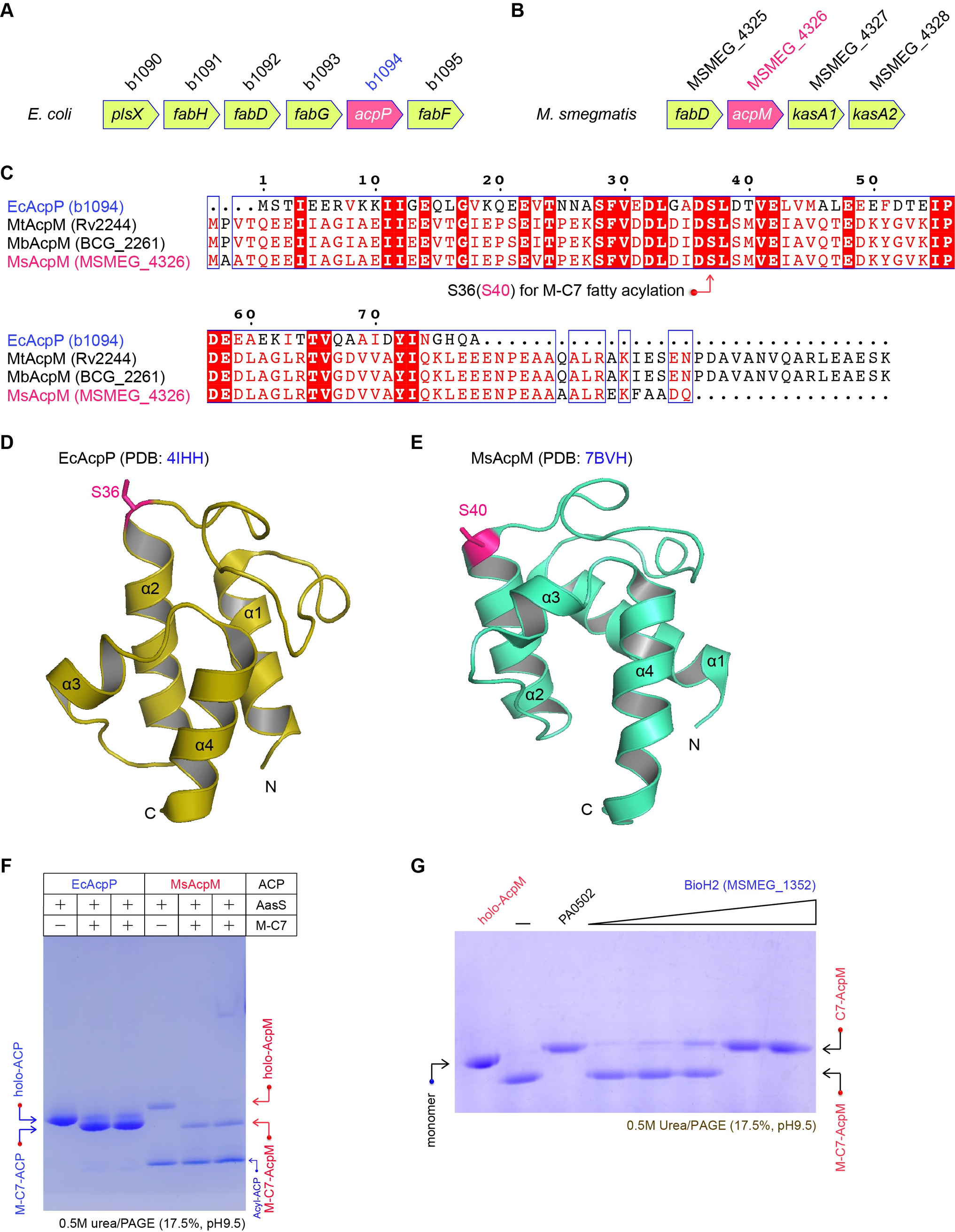

Supplement: S6 Fig — Genomic context of E.coli AcpP (A) and M. smegmatis AcpM (B). C. Sequence alignment of the three AcpM homologs of Mycobacteria with the paradigmatic AcpP of E. coli MG1655. D. Ribbon structure of the E. coli AcpP (PDB: 4IHH). E. Overall structure of mycobacterial AcpM (PDB: 7BVH). S36 (S40) colored hot pink, denotes the residue of AcpP attached with a methyl-pimeloyl moiety. Ribbon graphs were plotted with the software of PyMol (https://pymol.org/2). F. The attachment of M-C7 by AasS suggests functional equivalence between MsAcpM and EcAcpP. Similar to that of EcAcpP, MsAcpM is also ligated with M-C7 by AasS, giving M-C7-ACP. Conformationally-sensitive gel [0.5M urea/17.5% PAGE (pH9.5)] was applied to separate the M-C7-ACP product from its reactant holo-ACP. G. M-C7-AcpM is a functional substrate for BioH2(MSMEG_1352). Here, 2 pmol of Pseudomonas PA0502 protein, a known BioH esterase [87], was added as the positive control. The minus denotes no addition of PA0502 or BioH2(MSMEG_1352). The top triangle on right hand represents the enzyme BioH2 at various level ranging from 5, 10, 20, 60, to 80 pmol. Designations: AcpP, acyl carrier protein; AcpM, ACP of Mycobacteria; PlsX, Phosphate: acyl-ACP acyltransferase; FabH, β-ketoacyl-ACP synthase III; FabD, Malonyl-CoA: acyl trans-acylase; FabG, 3-oxoacyl-ACP reductase; FabF, 3-oxoacyl-ACP synthase; KasA1 & KasA2: two isoforms of FAS-II β-ketoacyl-ACP synthase with 65.06% identity; Ec, E. coli; Mt, Mycobacterium tuberculosis; Mb, Mycobacterium tuberculosis variant bovis BCG; Ms, Mycobacterium smegmatis MC2 155; N, N-terminus; C, C-terminus; α, α-helices; S36(40), Serine at the position of 36 (40); AasS, Acyl-ACP synthetase; M-C7, methyl-pimelic acid; M-C7-ACP, pimeloyl-ACP methyl ester. (TIF) [file ppat.1010615.s010.tif]

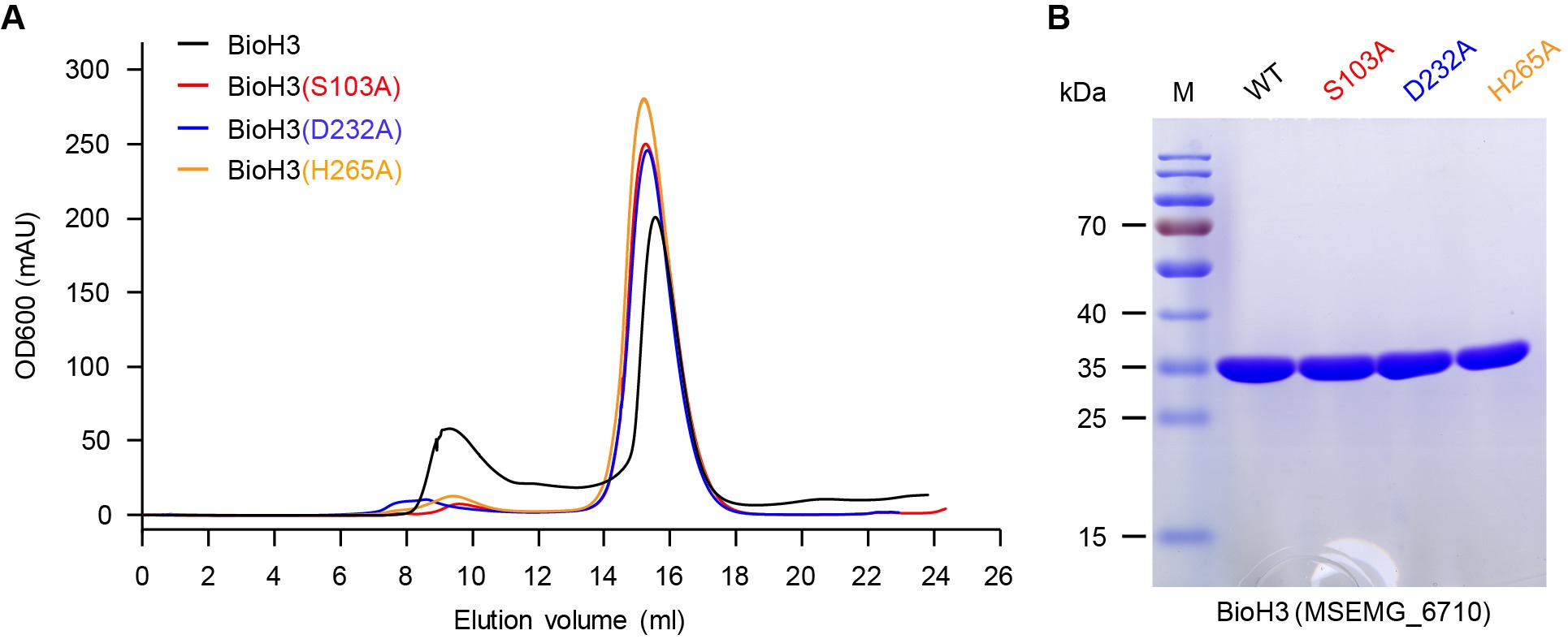

Supplement: S7 Fig — A. Size exclusion chromatography of BioH3 and its mutants with certain defection in the catalytic triad. B. SDS-PAGE profile for BioH3 and its three derivatives mutated in the catalytic triad. Namely, the three mutants included BioH3(S103A), BioH3(D248A), and BioH3(H265A). Designations: WT, Wild-type of BioH3; M, Protein marker; kDa, kilo-dalton. (TIF) [file ppat.1010615.s011.tif]

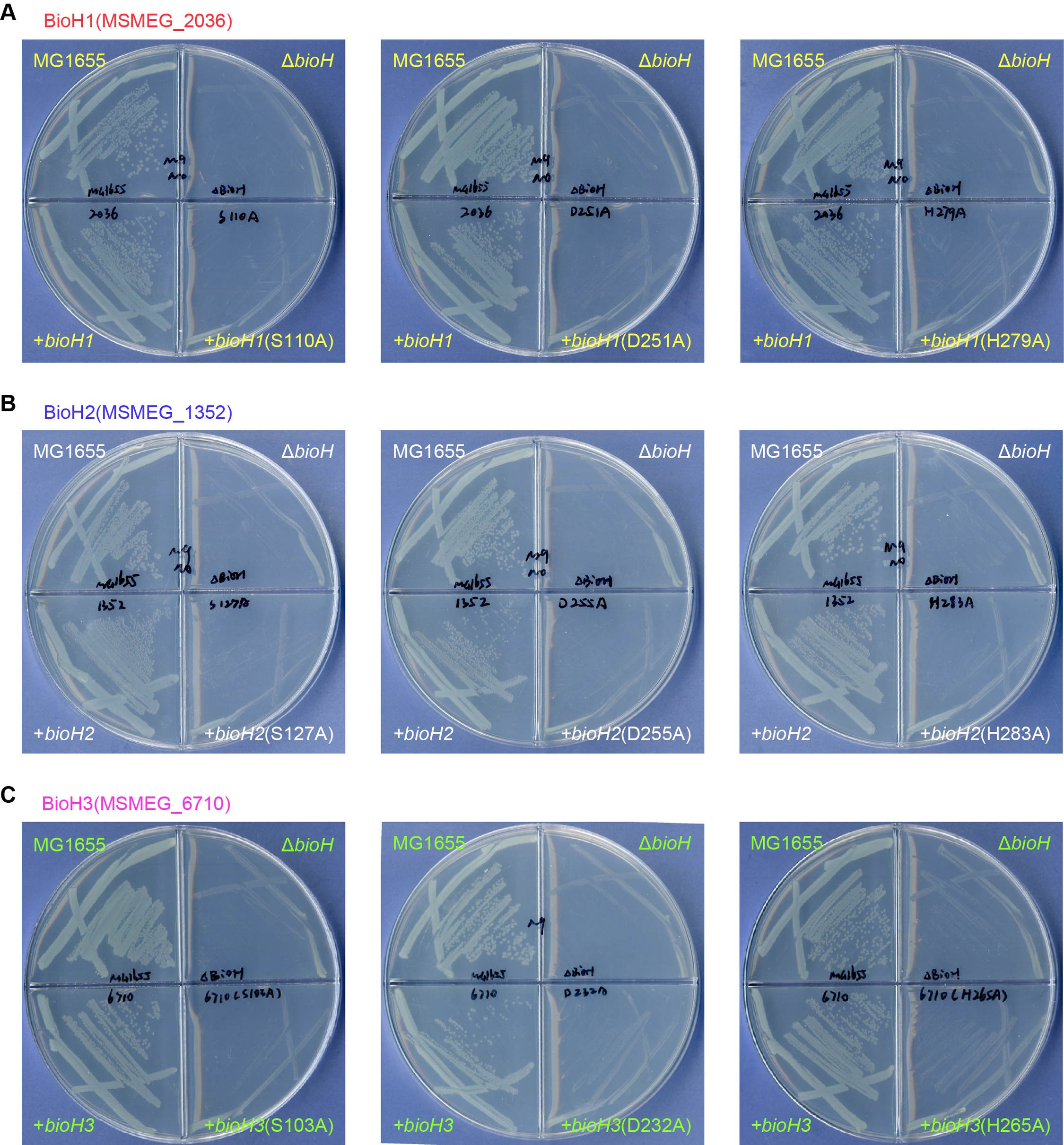

Supplement: S8 Fig — A. Use of structure-guided, site-directed mutagenesis to assay the function of the catalytic triad (S110, D251 & H279) of BioH1 (MSMEG_2036). B. Functional analysis for the catalytic triad (S127, D255 & H283) of BioH2 (MSMEG_1352). C. Functional dissection of the catalytic triad (S103, D232 & H265) from BioH3 (MSMEG_6710). To address effect on bioH by numbers of single mutations in catalytic triad, bacterial viabilities of the recipient hosts (arising from the ΔbioH biotin auxotrophic strain) were tested on the non-permissive condition lacking biotin. A representative result was given. (TIF) [file ppat.1010615.s012.tif]

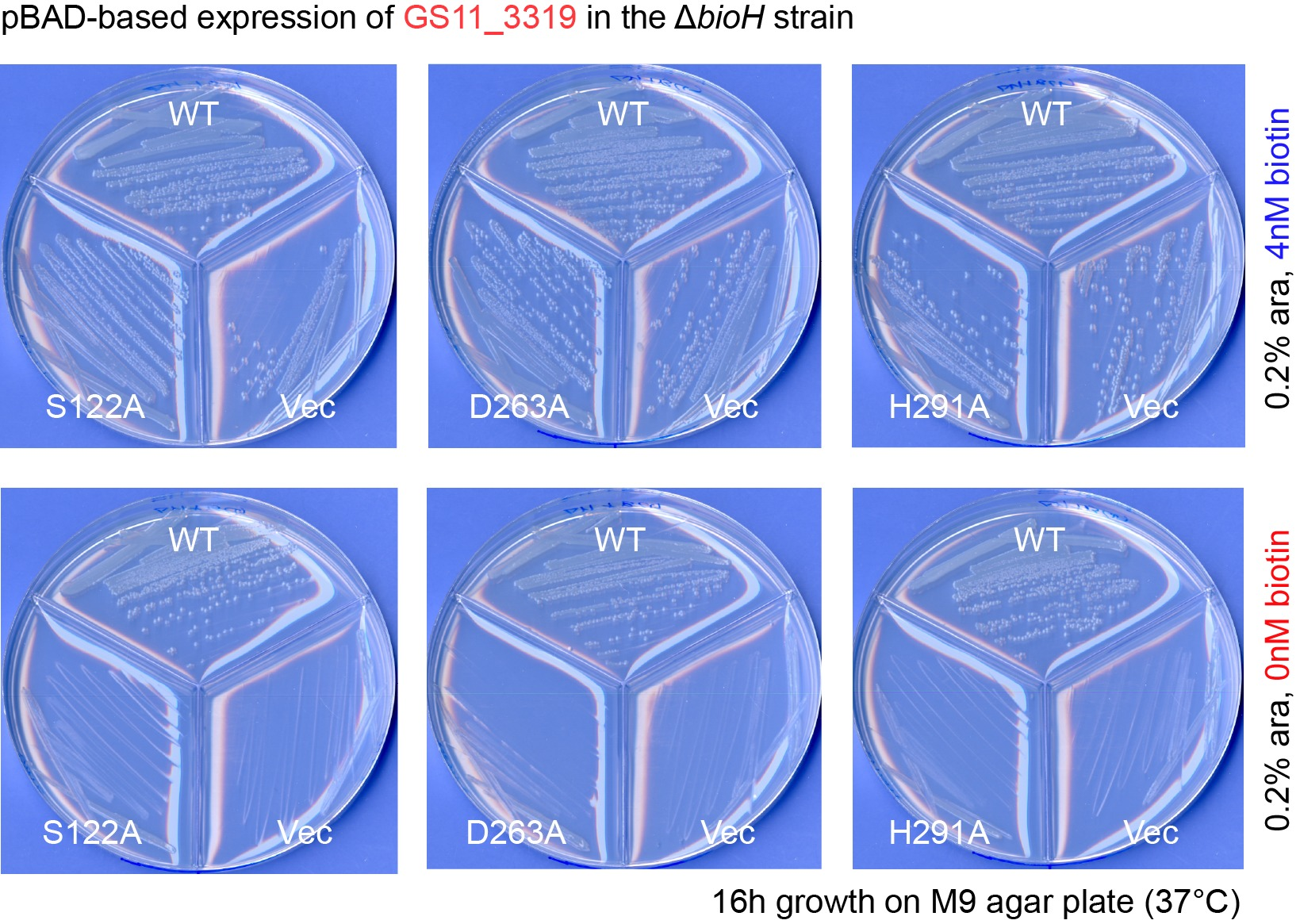

Supplement: S9 Fig — The catalytic triad of GS11_3319 is constituted by the following three residues, namely S122, D263, and H291. (TIF) [file ppat.1010615.s013.tif]

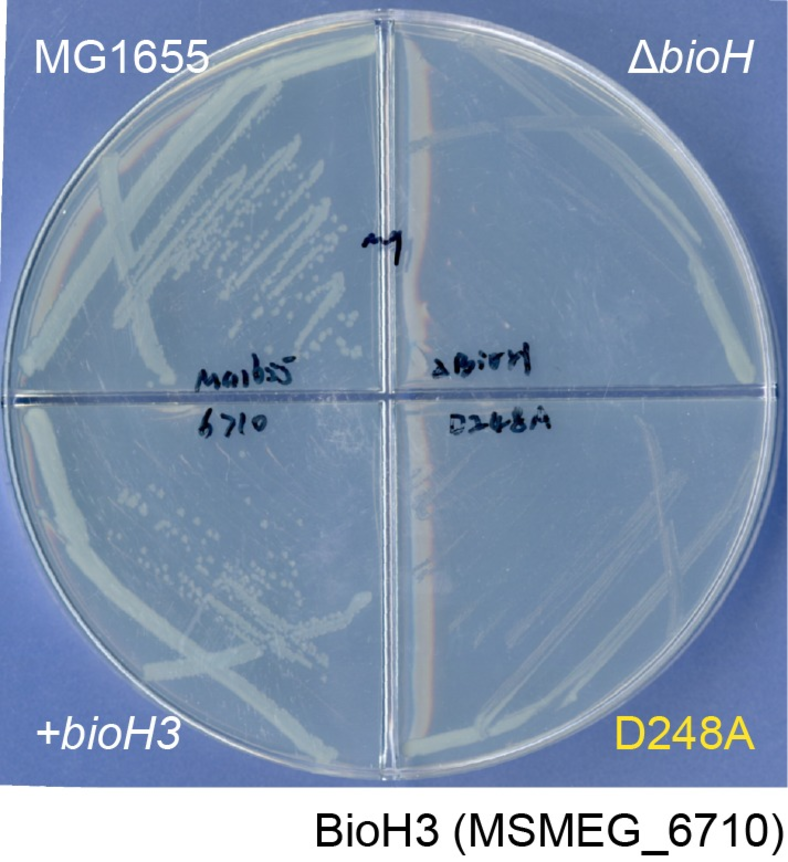

Supplement: S10 Fig — A representative result was shown. (TIF) [file ppat.1010615.s014.tif]

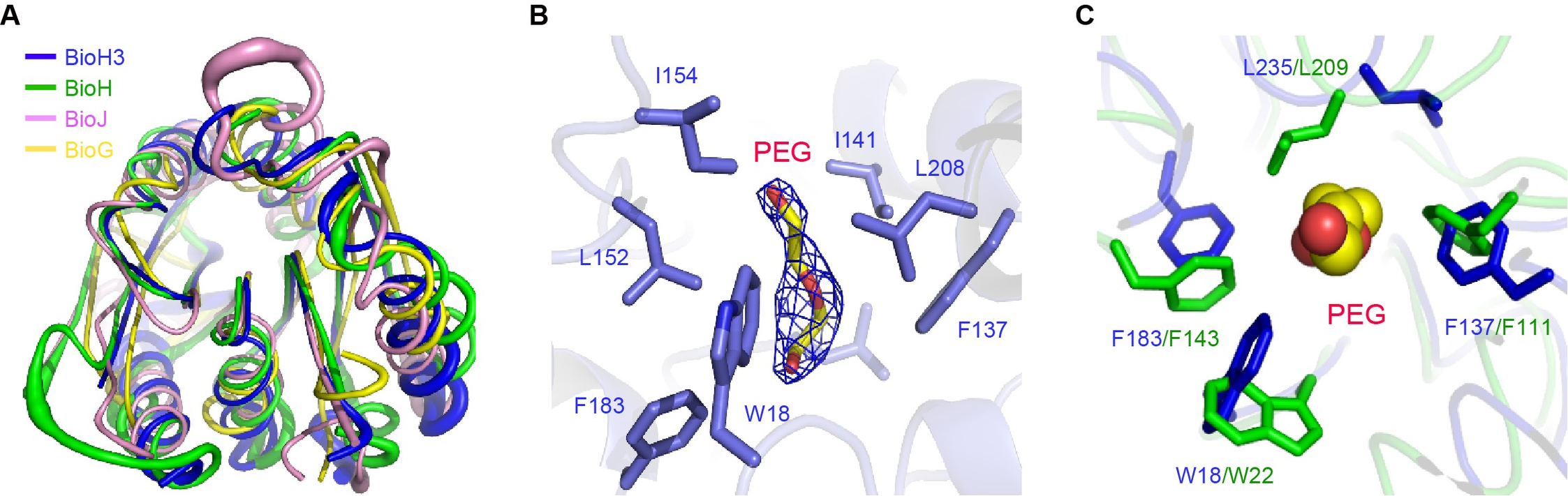

Supplement: S11 Fig — A. Structural superposition of the core domains from BioH3 and its three isoforms (BioH, BioG, and BioJ). They were separately colored blue for BioH3, green for BioH, yellow for BioG, and wheat for BioJ. B. Enlarged view of PEG-binding cavity of BioH3. Seven residues are proposed to surround PEG molecule. Namely, they are W18, F137, I141, L152, I154, F183, and L208. The 2Fo-Fc electron density maps of PEG were contoured at 1.2δ level. C. Conformational comparison of the PEG-mimicking substrate cavity of BioH3 with that of BioH. It elucidated that four of the PEG-binding residues are conserved, but vary dramatically in their orientations. Namely, they denoted i) W18, F137, F183 & L235 for BioH3; and ii) W22, F111, F143 & L209 for BioH. Residues of BioH3 and BioH were colored in blue and green, respectively. PEG was shown with spheres in atomic color. (TIF) [file ppat.1010615.s015.tif]

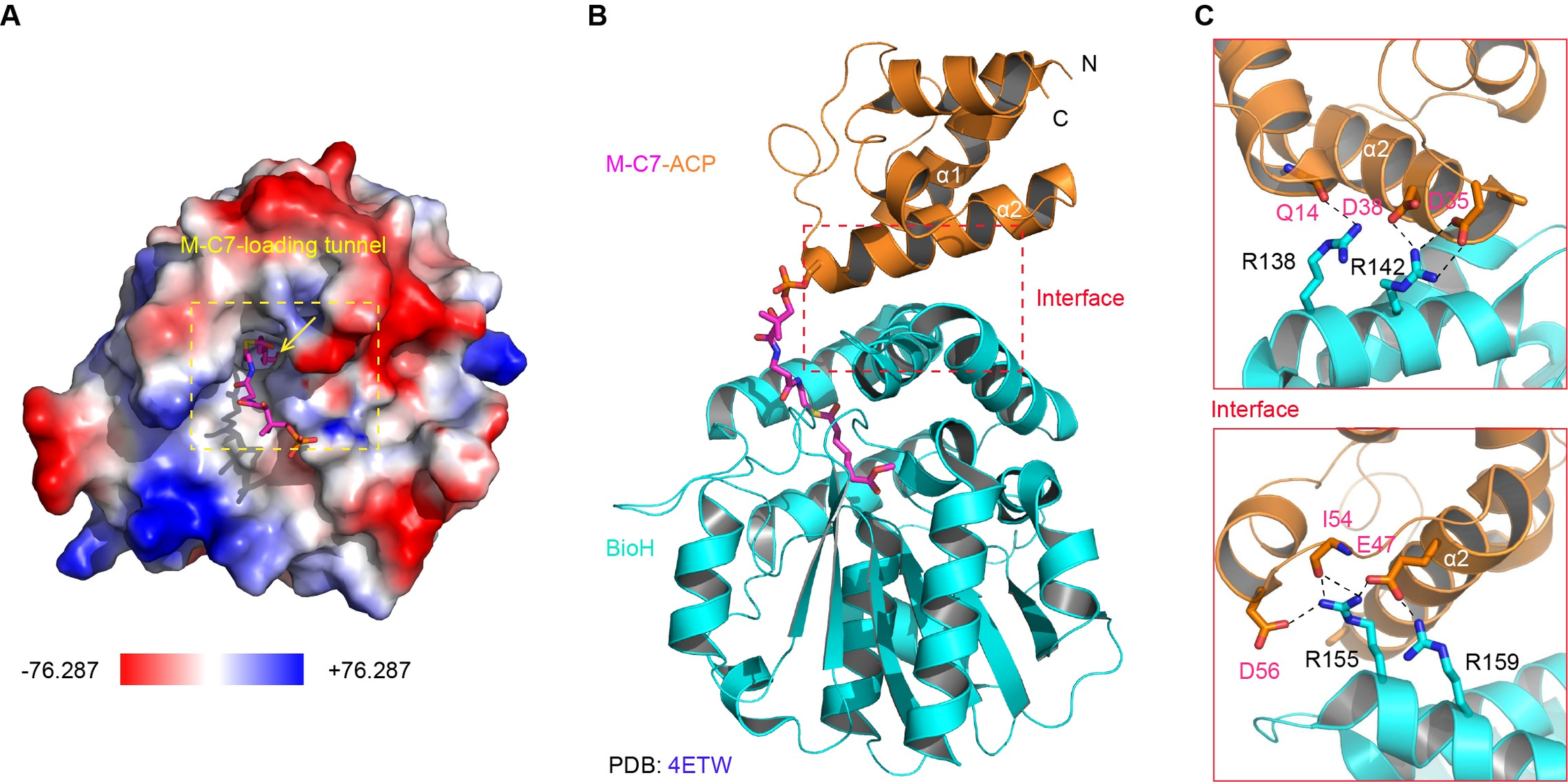

Supplement: S12 Fig — A. Surface structure of Shigella BioH demethylase. The M-C7-loading tunnel is highlighted with a dashed line rectangle. M-C7 is denoted with sticks and indicated with an arrow. Red denotes negative charge, and blue refers to positive charge. B. Ribbon structure of the complex of Shigella BioH and its substrate M-C7-ACP. The complex structure was generated using BioH/M-C7-ACP (PDB: 4ETW). As for ribbon structure, BioH is colored cyan, and M-C7-ACP is shown in orange. The ionic interaction between BioH and the α2-helices M-C7-ACP is underlined with a dashed line rectangle. M-C7 is denoted with purple sticks. C. Enlarged views of ionic interactions between BioH and the α2-helices of M-C7-ACP. Presumably, the first site of the ionic interaction engages two basic residues (R138 and R142) of BioH, pairing with three acidic amino acids (Q14, D35, and D38) from ACP α2-helices; the second site of salt bridge involves two positively-charged residues (R155 and R159) of BioH and three negatively-charged residues (E47, I54 & D56) of ACP α2-helices. Designations: α, α-helices; N, N-terminus; C, C-terminus; ACP, Acyl carrier protein; M-C7, methyl pimeloyl chain; M-C7-ACP, methyl pimeloyl-ACP ester. (TIF) [file ppat.1010615.s016.tif]

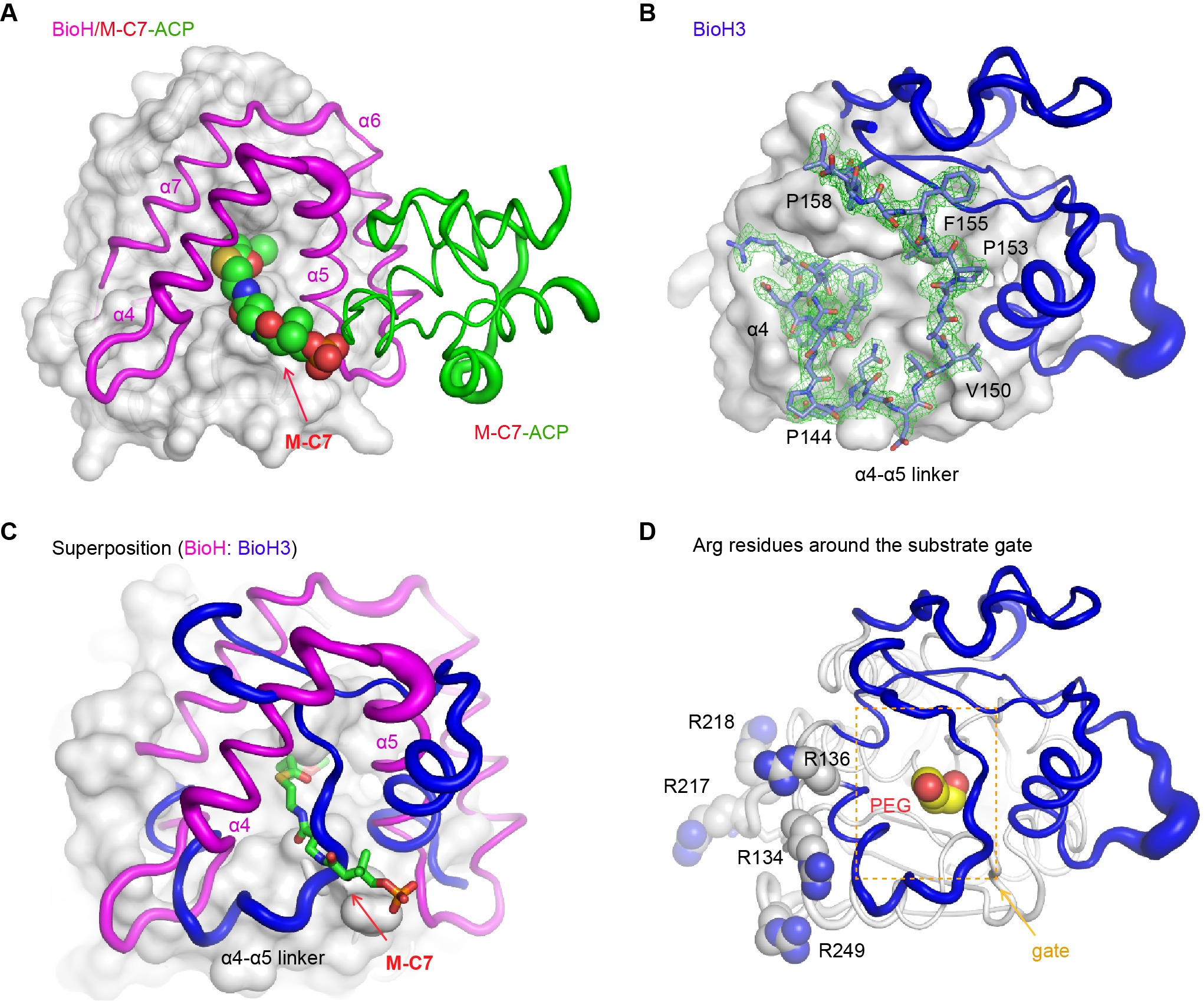

Supplement: S13 Fig — A. Overall folding of BioH complexed with its M-pim-ACP substrate. The core and lid domains of BioH were separately shown as white surface and magenta cartoon. M-pimeloyl moiety was indicated with spheres in atomic color, and its ACP cargo was shown in green cartoon. B. Crystal structure of BioH3 enzyme. The lid domain was given as cartoon in blue, whereas the gate region was shown as sticks outlined with 2Fo-Fc electron density maps (contour level, 1.2δ). Five residues are included, namely P144, V150, P153, F155, and P158. C. The M-C7 cavity gate of BioH3 inferred by its structural superposition with BioH. The core domains were displayed as white surface. The lid of BioH3 was given with blue cartoon, and the counterpart of BioH was shown as magenta cartoon. The M-C7 moiety indicated with an arrow, was denoted with a stick. The two α-helices (α4 and α5) of BioH that participate in its M-C7 moiety-loading cavity, are replaced by the α4-α5 linker in BioH3. D. Distribution of Arginine residues near the M-C7 cavity gate in the BioH3 structure. The PEG molecule mimicking the M-C7 fatty acyl chain, was indicated with spheres in atomic color. The substrate cavity gate is highlighted with dashed line. Five positively-charged basic residues around the cavity gate were presented with spheres in atomic color. Namely, they included R134, R136, R217, R218, and R249. (TIF) [file ppat.1010615.s017.tif]

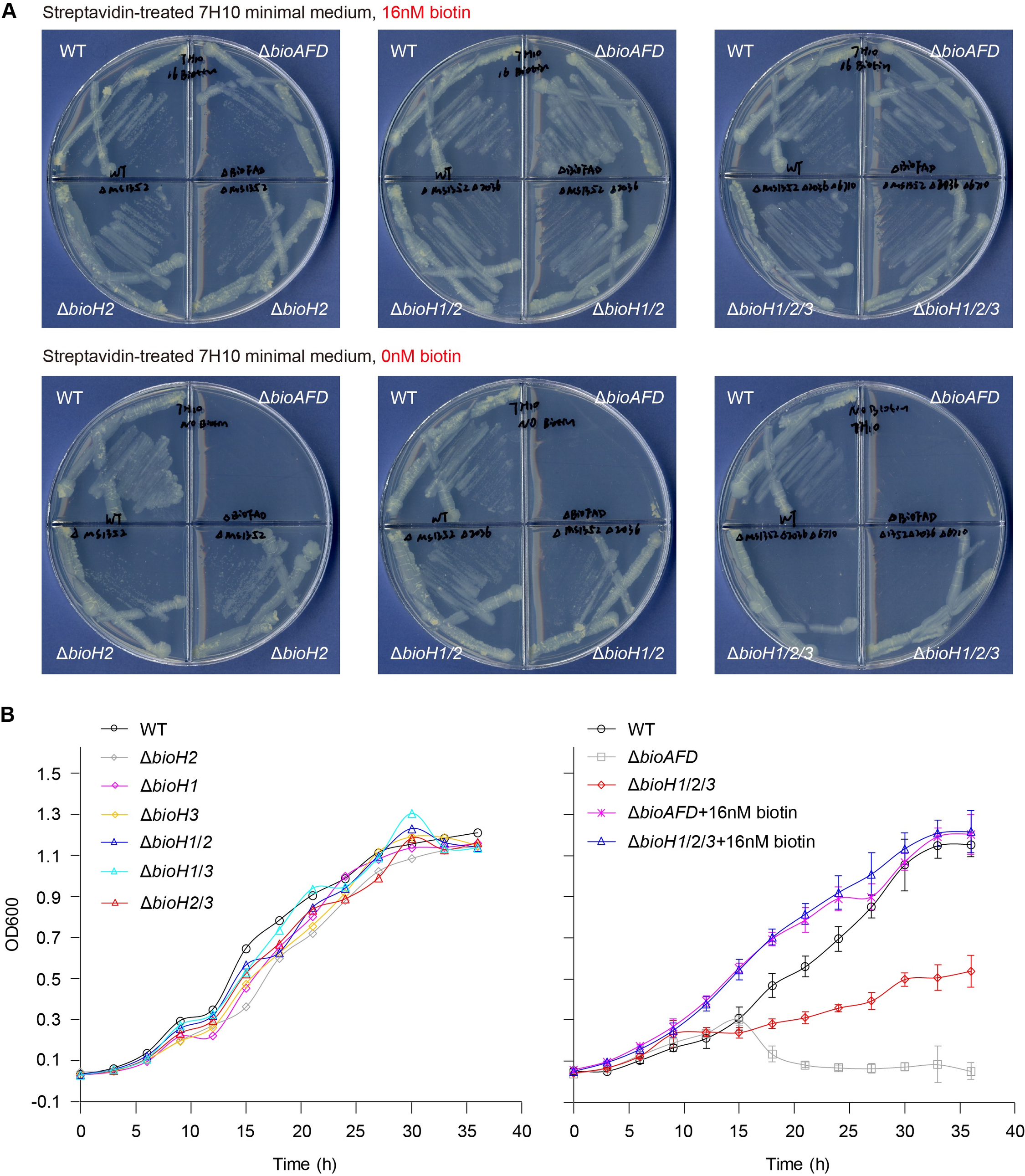

Supplement: S14 Fig — A. Bacterial viability on 7H10 minimal agar plates revealed that the triple bioH1/2/3 mutant (ΔbioH1/2/3) of M. smegmatis MC2 155 is biotin auxotroph, and can be rescued upon the addition of up to 16nM exogenous biotin. B. Use of growth curves enabled us to conclude that unlike the single/double mutant of bioH (H1 to H3) retaining robust viability, the triple mutant ΔbioH1/2/3 exhibits serious defection in bacterial growth. In addition to WT, eight mutants were tested here, namely i) ΔbioAFD, ii) three single mutants (ΔbioH1, ΔbioH2, and ΔbioH3) iii) three double mutants (ΔbioH1/2, ΔbioH1/3, and ΔbioH2/3), and iv) a triple mutant (ΔbioH1/2/3). The positive control referred to the ΔbioAFD mutant, a biotin auxotrophic strain. Designations: WT, M. smegmatis MC2 155. (TIF) [file ppat.1010615.s018.tif]
